# Supplementary material for: A δ-cell subpopulation with a pro-β-cell identity contributes to efficient age-independent recovery in a zebrafish model of diabetes
Source: eLife. 2022 Jan 21;11:e67576. doi: 10.7554/eLife.67576 (PMC8820734; doi:10.7554/eLife.67576)
Supplement: Figure 1—source data 1. [file elife-67576-fig1-data1.pdf]

Figure 1-Source Data 1

Blood glucose (mg/dl) in adult fish

CTL	3 dpt	20 dpt
61	426	149
53	600	190
51	283	104
61	600	88
62	600	102
51	600	82
49	600	177
55	600	101
64	493	121
62	505	111
60	517	132
50	568	127
58	426	123
61	600	67
96	283	139
61	600	127
62	600	87
95	600	103
81	600	103
50	600	149
58	493	104
61	505	88
96	517	102
61	568	82
62	308	177
51	232	101
49	232	121
86	285	111
64	580	132
73	600	127
90	600	123
81	600	67
104	600	139
89		127
69		87
61		103
62		103
95		141
55		161
64		
62		
60		
61		
53		
51		
