# Supplementary material for: A δ-cell subpopulation with a pro-β-cell identity contributes to efficient age-independent recovery in a zebrafish model of diabetes
Source: eLife. 2022 Jan 21;11:e67576. doi: 10.7554/eLife.67576 (PMC8820734; doi:10.7554/eLife.67576)
Supplement: Figure 1—source data 2. [file elife-67576-fig1-data2.pdf]

Figure 1-Source Data 2

Total islet cell number

CTL	3dpt	20dpt
13532	10740	12075
12419	14771	9827
14712	6795	9149
10016	8941	6833
15137	12361	10043
10646	12701	14604
18386	6317	10789
11648	8122	10684
8907		
16418		
18033		

ins:mCherry+ (GFP-) cells

CTL	3dpt	20dpt
2784	64	49
2929	203	8
2750	218	8
2609	122	10
5034	62	14
1512	88	12
5215	47	5
3011	38	5
1837		
4008		
4354		

sst1.1:GFP+ (mCherry-) cells

CTL	3dpt	20dpt
2030	2208	741
2558	3614	2014
4002	1984	1373
2499	2561	1638
3299	1838	2378
2342	2981	2018
3147	1954	957
2594	1875	1432
2110		
3181		
3403		
