# Supplementary material for: A δ-cell subpopulation with a pro-β-cell identity contributes to efficient age-independent recovery in a zebrafish model of diabetes
Source: eLife. 2022 Jan 21;11:e67576. doi: 10.7554/eLife.67576 (PMC8820734; doi:10.7554/eLife.67576)
Supplement: Figure 1—source data 3. — mCherry+ cells from the main islet were sorted by FACS from Tg(ins:NTR-P2A-mCherry) adult zebrafish 2months after β-cell ablation and gene expression levels were determined by RNA sequencing (expressed as normalized read counts). The ins gene is the highest expressed gene just above sst1.1. This is the result of one single exploratory replicate. [file elife-67576-fig1-data3.docx]

| **Gene_Name** | **Expression in regenerated β-cells** |
| --- | --- |
| ins | 14801223 |
| sst1.1 | 7912534 |
| mt-co1 | 435554 |
| ppdpfb | 267739 |
| rgs5a | 256637 |
| dkk3b | 213588 |
| mt-co2 | 210445 |
| RPL41 | 206138 |
| scg3 | 146678 |
| calca | 143619 |
| pcsk1 | 137921 |
| eef1a1l2 | 133915 |
| fosab | 130543 |
| tmsb1 | 130150 |
| cst3 | 125586 |
| mt-co3 | 107003 |
| rpl19 | 95153 |
| pcsk2 | 94936 |
| rpsa | 91191 |
| mt-cyb | 89586 |

**Figure 1- source data 3**
