# Supplementary material for: A δ-cell subpopulation with a pro-β-cell identity contributes to efficient age-independent recovery in a zebrafish model of diabetes
Source: eLife. 2022 Jan 21;11:e67576. doi: 10.7554/eLife.67576 (PMC8820734; doi:10.7554/eLife.67576)
Supplement: Figure 3—source data 1. [file elife-67576-fig3-data1.pdf]

Figure 3-Source Data 1. Significantly differentially expressed genes between β-cells (CTL_Beta) and bihormonal cells (20dpt_Bihormonal) from DESeq (Padj<0.05)

Ensembl_ID	Gene_Name	Mean_ CTL_Beta	Mean_ 20dpt_Bihormonal	Log2FC	Padj
ENSDARG00000061140	colec12	0	265	8,19	4,32E-12
ENSDARG00000039067	srd5a2b	11	3602	7,97	7,35E-20
ENSDARG00000098231	HHEX	2	491	7,75	8,43E-19
ENSDARG00000018817	bdnf	7	2437	7,73	2,37E-16
ENSDARG00000040799	sst1.1	12837	6638811	7,71	1,46E-13
ENSDARG00000044691	ppp1r3b	1	568	7,67	2,32E-11
ENSDARG00000102687	CABZ01085139.1	14	5046	7,54	5,06E-14
ENSDARG00000099732	gfra1a	8	2869	7,37	2,39E-13
ENSDARG00000103295	cyp3a65	21	5050	7,26	2,63E-15
ENSDARG00000101095	cpne4a	15	3272	7,16	2,56E-15
ENSDARG00000007682	ppdpfa	75	16778	7,04	1,46E-13
ENSDARG00000017790	chrnb2b	0	110	6,93	6,33E-11
ENSDARG00000055154	slc8a4a	1	231	6,86	2,31E-11
ENSDARG00000099446	slit1b	0	490	6,81	1,02E-07
ENSDARG00000105201	adcyap1r1a	9	1216	6,75	2,57E-17
ENSDARG00000101508	nr6a1a	1	294	6,73	2,11E-12
ENSDARG00000032639	cd36	2	362	6,72	3,60E-14
ENSDARG00000000943	dzip1	1	132	6,71	8,86E-13
ENSDARG00000069254	chrm4a	9	1415	6,63	1,46E-13
ENSDARG00000052012	rtn4rl2a	3	453	6,61	6,29E-13
ENSDARG00000001912	drd4-rs	2	365	6,53	7,88E-12
ENSDARG00000045750	syt10	5	591	6,50	2,63E-15
ENSDARG00000043589	ca4a	58	9131	6,48	5,03E-12
ENSDARG00000055565	cacnb2b	2	252	6,46	1,71E-14
ENSDARG00000069028	rxfp3.3a1	3	424	6,45	2,30E-13
ENSDARG00000069698	lrguk	7	808	6,43	1,68E-14
ENSDARG00000076351	brinp3a.1	0	90	6,30	8,08E-09
ENSDARG00000093714	artnb	1	102	6,30	1,13E-10
ENSDARG00000079907	ptger4c	0	239	6,27	1,33E-08
ENSDARG00000111920	cplx1	1	128	6,24	1,55E-11
ENSDARG00000038489	b3gnt7	3	302	6,23	2,30E-13
ENSDARG00000002533	adora2b	1	136	6,20	2,48E-09
ENSDARG00000098289	vstm2l	1	181	6,20	1,01E-11
ENSDARG00000020289	pif1	0	121	6,18	3,03E-08
ENSDARG00000005780	npy8br	2	255	6,16	2,63E-15
ENSDARG00000035198	gcnt4a	0	130	6,16	2,58E-08
ENSDARG00000036036	mdka	2	235	6,16	1,34E-11
ENSDARG00000104005	slc4a3	6	535	6,15	8,43E-19
ENSDARG00000099708	cacna1hb	0	62	6,08	7,20E-08
ENSDARG00000055026	ptch2	0	41	5,97	2,64E-08
ENSDARG00000054253	sobpa	1	96	5,91	2,69E-11
ENSDARG00000069294	insl5b	2	1242	5,86	2,48E-09
ENSDARG00000114760	asip2b	5	967	5,81	9,78E-08
ENSDARG00000074317	fam20ca	1	80	5,77	9,44E-10
ENSDARG00000079978	samd10a	1	68	5,74	2,87E-09
ENSDARG00000062165	tub	2	179	5,73	8,31E-13
ENSDARG00000016793	crybb1l2	1	174	5,66	1,58E-07
ENSDARG00000032743	si:dkey-11p23.7	0	35	5,60	1,11E-07
ENSDARG00000114192	BX548011.5	3	400	5,56	2,79E-06
ENSDARG00000004378	yrk	7	468	5,53	3,23E-12
ENSDARG00000075858	fam155a	1	64	5,50	2,94E-10
ENSDARG00000091073	alkal2a	0	57	5,47	3,26E-07
ENSDARG00000045880	gas2l3	7	360	5,38	1,11E-13
ENSDARG00000087241	ucn3l	69	4267	5,37	2,29E-10
ENSDARG00000091277	adra2da	12	607	5,34	2,76E-12
ENSDARG00000011672	sema3b	3	169	5,32	7,38E-14
ENSDARG00000018643	igf2a	0	62	5,32	2,53E-06
ENSDARG00000096739	si:dkey-219e21.2	0	43	5,31	2,07E-08
ENSDARG00000101481	rbp5	1	76	5,29	8,57E-11

ENSDARG00000044954	slit1a	7	304	5,28	1,12E-15
ENSDARG00000009418	mef2cb	1	81	5,27	6,33E-11
ENSDARG00000040610	si:ch73-352p4.8	1	58	5,27	8,86E-07
ENSDARG00000068247	luzp2	19	798	5,24	1,02E-16
ENSDARG00000028845	calcr	0	31	5,21	7,25E-05
ENSDARG00000068726	masp1	0	47	5,21	6,49E-07
ENSDARG00000079542	stard13a	5	220	5,20	4,26E-12
ENSDARG00000055539	epdl2	0	88	5,19	4,51E-06
ENSDARG00000101234	nsmfb	1	88	5,18	3,59E-08
ENSDARG00000042816	mmp9	7	362	5,18	3,41E-11
ENSDARG00000073963	si:ch211-258f14.2	3	176	5,15	5,51E-08
ENSDARG00000036965	rnf24	0	22	5,14	2,85E-08
ENSDARG00000031751	npr1a	27	1049	5,14	7,20E-17
ENSDARG00000100739	CABZ01052573.1	0	33	5,10	5,12E-06
ENSDARG00000094132	igf1	1	43	5,10	9,20E-07
ENSDARG00000058460	cacng3a	0	63	5,09	8,04E-06
ENSDARG00000055284	afap1	1	72	5,09	1,75E-08
ENSDARG00000078322	col12a1a	1	78	5,08	1,18E-07
ENSDARG00000073957	tox3	11	386	5,06	3,98E-18
ENSDARG00000113674	CABZ01058222.1	7	307	4,99	1,62E-10
ENSDARG00000011797	fam46bb	2	82	4,95	4,19E-08
ENSDARG00000056888	dnah5l	1	67	4,95	8,11E-10
ENSDARG00000040338	hvcn1	1	71	4,91	4,57E-09
ENSDARG00000102636	trmt9b	17	618	4,89	4,26E-12
ENSDARG00000055843	cdh10a	5	288	4,89	1,11E-07
ENSDARG00000013312	cadps2	0	38	4,86	8,43E-06
ENSDARG00000098295	efna3b	0	26	4,85	1,50E-06
ENSDARG00000007950	itga11b	0	35	4,81	1,07E-06
ENSDARG00000061819	wscd2	4	143	4,80	1,46E-13
ENSDARG00000033161	sst1.2	841	37226	4,77	6,79E-09
ENSDARG00000069134	vwc2l	1	52	4,77	6,88E-08
ENSDARG00000060457	pmp22b	5	170	4,77	7,74E-11
ENSDARG00000055123	kcnk2a	0	29	4,76	6,99E-06
ENSDARG00000067781	urp2	7	349	4,76	1,12E-07
ENSDARG00000088072	usp43b	1	34	4,76	1,35E-08
ENSDARG00000043323	lnx1	4	138	4,74	1,48E-10
ENSDARG00000035899	lingo1b	0	57	4,73	2,24E-05
ENSDARG00000035966	pgr	41	1345	4,72	2,31E-11
ENSDARG00000020031	cldn11a	1	41	4,70	5,38E-07
ENSDARG00000095788	ghrhrl	0	24	4,68	6,80E-06
ENSDARG00000003293	sox9a	4	157	4,67	9,54E-09
ENSDARG00000023316	sim1a	36	1133	4,67	3,51E-11
ENSDARG00000037455	ugt8	4	145	4,66	7,74E-11
ENSDARG00000070522	cacna1i	10	293	4,65	2,92E-12
ENSDARG00000019772	si:ch1073-280e3.1	2	75	4,65	4,95E-08
ENSDARG00000024167	kcnh1a	1	50	4,65	2,99E-07
ENSDARG00000090624	ADGRL3	9	310	4,63	7,38E-10
ENSDARG00000003991	fhl2b	0	37	4,61	1,48E-05
ENSDARG00000002336	dlc	70	2389	4,61	1,21E-08
ENSDARG00000073713	vav3b	24	773	4,60	2,48E-09
ENSDARG00000010816	slc7a14a	42	1083	4,59	9,91E-18
ENSDARG00000054055	ostn	2	320	4,58	1,12E-05
ENSDARG00000042646	zgc:77784	2	61	4,55	2,41E-08
ENSDARG00000058348	scinlb	17	462	4,54	2,30E-13
ENSDARG00000073872	tpst1	11	316	4,53	2,32E-11
ENSDARG00000025017	prdm8	0	98	4,53	3,36E-05
ENSDARG00000100267	FO904977.1	2	50	4,53	2,84E-10
ENSDARG00000099979	tgfbr3	1	42	4,53	2,83E-07
ENSDARG00000044541	ppp1r14ba	1	31	4,52	2,44E-08
ENSDARG00000099211	si:dkey-220o5.5	0	42	4,51	3,76E-05
ENSDARG00000062420	nfia	0	24	4,50	6,05E-06
ENSDARG00000027065	slc38a3a	1	43	4,49	3,67E-08
ENSDARG00000077298	gas1a	11	293	4,48	6,05E-13
ENSDARG00000079462	si:ch211-131k2.3	1	39	4,48	9,71E-07
ENSDARG00000100053	CABZ01044099.1	1	26	4,48	2,22E-06

ENSDARG00000089221	si:ch211-195o20.7	1	40	4,47	2,43E-07
ENSDARG00000063097	scube1	3	93	4,45	1,92E-09
ENSDARG00000015495	klf3	11	303	4,45	8,03E-10
ENSDARG00000055833	si:dkey-216e24.3	1	41	4,42	5,76E-06
ENSDARG00000102702	lrrn3b	8	205	4,42	6,09E-14
ENSDARG00000078560	slc9a5	12	298	4,40	1,04E-11
ENSDARG00000043664	si:dkey-63b1.1	2	78	4,39	9,14E-06
ENSDARG00000058464	rasd3	5	141	4,39	8,52E-08
ENSDARG00000069160	fam19a5a	3	74	4,37	7,12E-12
ENSDARG00000004074	bach2b	2	47	4,36	4,96E-08
ENSDARG00000105113	galr2b	1	66	4,36	1,90E-04
ENSDARG00000111585	CABZ01048666.1	1	26	4,36	1,27E-05
ENSDARG00000054321	ngs	0	27	4,36	1,24E-05
ENSDARG00000070000	txnipb	0	111	4,35	5,53E-05
ENSDARG00000068892	sostdc1a	4	151	4,32	1,03E-05
ENSDARG00000070683	dkk3b	7150	167305	4,32	4,01E-11
ENSDARG00000040649	prickle1a	3	86	4,31	4,06E-08
ENSDARG00000100128	CU861477.1	4	119	4,31	7,91E-08
ENSDARG00000005894	slc7a9	6	154	4,30	1,02E-07
ENSDARG00000087601	gpr153	7	163	4,30	1,01E-09
ENSDARG00000090190	bcam	2	53	4,30	1,06E-09
ENSDARG00000015515	mc4r	15	341	4,29	6,33E-11
ENSDARG00000056909	LO018309.1	2	74	4,27	3,14E-07
ENSDARG00000058179	ackr3b	0	26	4,27	2,64E-06
ENSDARG00000067848	nmrk2	4	119	4,26	1,33E-07
ENSDARG00000045016	npffl	2	52	4,25	8,60E-07
ENSDARG00000092057	si:dkey-90l23.1	154	4570	4,24	5,03E-06
ENSDARG00000059798	kcnq1	0	23	4,23	5,16E-04
ENSDARG00000098221	st8sia1	1	43	4,23	1,05E-06
ENSDARG00000061940	htr4	0	65	4,22	1,13E-04
ENSDARG00000004576	plk4	5	141	4,18	4,99E-07
ENSDARG00000028899	tekt4	1	38	4,17	7,51E-07
ENSDARG00000012306	syt13	1	40	4,15	2,55E-05
ENSDARG00000090686	slc35f2l	1	33	4,14	2,50E-06
ENSDARG00000019096	myl7	6	152	4,12	3,87E-07
ENSDARG00000007245	rundc3aa	1	24	4,12	4,12E-07
ENSDARG00000062447	hecw2a	10	210	4,12	7,18E-09
ENSDARG00000099903	si:ch211-193l2.10	20	401	4,11	2,01E-11
ENSDARG00000035009	trim35-27	1	37	4,10	2,40E-07
ENSDARG00000063475	abcg1	2	55	4,08	8,51E-09
ENSDARG00000102336	FQ377903.2	1	58	4,06	3,45E-04
ENSDARG00000055226	slc7a7	27	521	4,06	6,32E-11
ENSDARG00000021909	VSTM2A	6	114	4,03	2,94E-10
ENSDARG00000099133	si:ch73-72b7.1	1	22	4,01	2,77E-06
ENSDARG00000060927	si:ch211-136a13.1	2	83	3,99	5,26E-05
ENSDARG00000060025	nsmfa	2	55	3,99	1,43E-06
ENSDARG00000086665	si:dkey-175g6.2	46	1012	3,99	5,97E-07
ENSDARG00000103961	poln	2	37	3,98	7,08E-07
ENSDARG00000034344	pcdh19	13	296	3,98	1,93E-06
ENSDARG00000057671	epas1b	23	420	3,97	2,96E-10
ENSDARG00000058053	serping1	2	39	3,97	1,05E-06
ENSDARG00000087599	TENM3	27	458	3,94	4,16E-13
ENSDARG00000104315	fhl3b	3	80	3,93	2,16E-06
ENSDARG00000079578	rbpms2b	2	45	3,93	8,49E-09
ENSDARG00000055099	fam184b	7	118	3,91	3,97E-11
ENSDARG00000104361	barhl2	3	66	3,91	1,14E-05
ENSDARG00000079738	znf219	2	38	3,90	1,32E-06
ENSDARG00000002131	celf2	11	184	3,90	6,71E-13
ENSDARG00000006849	asic2	1	32	3,89	3,80E-07
ENSDARG00000055232	fancc	4	66	3,88	3,80E-11
ENSDARG00000028824	slc34a1a	11	184	3,87	6,81E-10
ENSDARG00000067545	adam19b	12	227	3,87	1,68E-07
ENSDARG00000037553	il1rapl2	2	40	3,86	4,26E-07
ENSDARG00000009281	dnm1b	8	134	3,86	1,54E-09
ENSDARG00000079414	sez6b	9	162	3,85	2,05E-07

ENSDARG00000046013	rasl11a	2	86	3,83	5,88E-04
ENSDARG00000024017	mdga2a	5	92	3,83	3,29E-10
ENSDARG00000069765	syngap1b	1	30	3,83	1,88E-06
ENSDARG00000070735	rnd2	1	22	3,83	7,91E-06
ENSDARG00000087633	si:dkey-11o18.5	1	28	3,83	1,86E-05
ENSDARG00000104664	dclk1b	12	220	3,82	2,98E-07
ENSDARG00000061249	myom1a	8	164	3,82	2,33E-06
ENSDARG00000052207	c3a.3	2	143	3,81	6,41E-04
ENSDARG00000029219	avpr2ab	3	56	3,81	1,37E-07
ENSDARG00000068182	crb3b	5	90	3,80	2,69E-06
ENSDARG00000054023	ttll6	4	60	3,79	4,09E-09
ENSDARG00000058471	plk1	2	56	3,79	3,89E-05
ENSDARG00000036670	aplnrb	0	351	3,75	3,80E-06
ENSDARG00000078953	epn3a	25	352	3,75	1,44E-13
ENSDARG00000060238	uacab	18	262	3,75	1,71E-14
ENSDARG00000093874	si:dkey-196j8.2	1	30	3,74	2,33E-05
ENSDARG00000075956	faxca	15	233	3,72	1,55E-09
ENSDARG00000074335	boc	52	823	3,71	5,49E-08
ENSDARG00000061240	cbln4	11	304	3,71	5,25E-04
ENSDARG00000021255	arhgap22	1	24	3,71	8,37E-06
ENSDARG00000024032	coch	4	93	3,68	1,56E-03
ENSDARG00000026926	rom1b	0	97	3,65	6,97E-05
ENSDARG00000019658	pou2f2a	5	87	3,64	2,41E-07
ENSDARG00000078671	cdk5r2b	7	105	3,62	6,70E-09
ENSDARG00000037402	lim2.3	2	34	3,61	3,78E-05
ENSDARG00000102335	ano1	9	153	3,61	2,21E-05
ENSDARG00000035832	pyyb	461	6393	3,59	6,52E-09
ENSDARG00000003411	foxa2	97	1192	3,58	9,91E-18
ENSDARG00000036008	caly	274	3688	3,58	1,55E-09
ENSDARG00000070743	tmem45a	9	124	3,57	1,21E-08
ENSDARG00000011929	plp1b	259	3370	3,57	1,46E-10
ENSDARG00000056080	si:dkey-191g9.5	2	42	3,56	6,11E-05
ENSDARG00000036820	mgll	5	72	3,56	3,12E-10
ENSDARG00000074258	gramd1bb	4	56	3,55	1,11E-08
ENSDARG00000077811	sox11a	13	171	3,54	4,05E-10
ENSDARG00000104501	FP236812.1	1	34	3,53	4,31E-04
ENSDARG00000056922	ltbp1	7	98	3,53	3,40E-07
ENSDARG00000003615	slc26a3.2	50	678	3,52	7,63E-08
ENSDARG00000006151	pde11al	3	67	3,52	2,99E-04
ENSDARG00000062687	kcnh7	17	226	3,51	1,34E-08
ENSDARG00000099101	gch2	153	2440	3,50	2,95E-05
ENSDARG00000102398	cnn1b	2	23	3,45	1,68E-06
ENSDARG00000076052	sez6l2	11	145	3,44	1,27E-07
ENSDARG00000024101	adra1d	9	129	3,42	8,75E-06
ENSDARG00000112049	cldn15a	1	23	3,41	1,62E-04
ENSDARG00000070220	pou3f2a	3	34	3,40	6,56E-07
ENSDARG00000061692	yjefn3	4	55	3,40	2,05E-06
ENSDARG00000107441	CABZ01063170.1	15	179	3,40	4,35E-09
ENSDARG00000114852	CR974456.1	2	29	3,40	2,76E-07
ENSDARG00000030289	jag1a	2	33	3,39	7,55E-07
ENSDARG00000040280	zgc:110182	5	80	3,39	9,09E-05
ENSDARG00000068144	nmba	7	127	3,38	5,48E-05
ENSDARG00000075393	spock2	1	26	3,38	8,76E-05
ENSDARG00000061248	xylt1	13	152	3,37	1,93E-08
ENSDARG00000013343	kif26ba	15	187	3,37	3,57E-07
ENSDARG00000100594	sez6l	11	140	3,37	3,34E-06
ENSDARG00000007436	avpr2aa	102	1276	3,36	1,34E-06
ENSDARG00000062376	nlgn3b	3	45	3,36	3,89E-06
ENSDARG00000087646	runx1	2	37	3,35	3,00E-04
ENSDARG00000043406	slc8a1b	3	40	3,35	5,62E-06
ENSDARG00000025679	comtb	14	161	3,35	1,51E-07
ENSDARG00000095743	sox11b	48	568	3,34	2,08E-07
ENSDARG00000056519	si:dkey-280e21.3	14	154	3,33	3,10E-09
ENSDARG00000032795	tmem8a	1	20	3,30	9,88E-05
ENSDARG00000058371	krt5	1	21	3,30	1,43E-03

ENSDARG00000101534	rab3db	38	392	3,30	4,96E-12
ENSDARG00000098802	map7d1a	2	20	3,29	1,70E-06
ENSDARG00000053724	adcyap1r1b	1	21	3,27	6,79E-06
ENSDARG00000062277	ppp2r2cb	2	27	3,27	7,03E-04
ENSDARG00000115070	si:dkeyp-74b6.2	29	320	3,26	2,40E-07
ENSDARG00000079372	si:ch211-264f5.6	27	289	3,25	6,70E-09
ENSDARG00000056427	auts2a	20	205	3,23	3,76E-10
ENSDARG00000022660	armc2	2	22	3,21	2,29E-04
ENSDARG00000116062	ADAM12 (1 of man	5	57	3,19	2,39E-05
ENSDARG00000011821	plod2	282	2665	3,17	6,82E-12
ENSDARG00000074329	arvcfa	25	302	3,16	2,03E-04
ENSDARG00000101777	pdlim5a	33	326	3,12	2,74E-07
ENSDARG00000024654	pgm3	306	2866	3,12	1,54E-09
ENSDARG00000100426	fam167b	9	90	3,12	1,18E-07
ENSDARG00000018846	dgat2	11	151	3,11	8,23E-04
ENSDARG00000016551	iqsec1b	121	1073	3,08	8,31E-13
ENSDARG00000078241	map7a	2	26	3,08	3,35E-05
ENSDARG00000104480	hcn1	15	148	3,06	9,85E-07
ENSDARG00000056745	necab2	35	347	3,06	7,49E-06
ENSDARG00000069402	lrrc4.1	6	57	3,03	2,60E-06
ENSDARG00000102972	CABZ01075125.1	10	95	3,02	1,11E-05
ENSDARG00000100900	si:ch211-183d5.2	114	1602	3,00	5,20E-03
ENSDARG00000062579	kremen1	38	328	3,00	3,58E-09
ENSDARG00000076294	klb	138	1250	2,99	1,25E-06
ENSDARG00000070972	si:ch211-81a5.8	5	50	2,97	8,11E-05
ENSDARG00000074183	si:dkey-172j4.3	2	19	2,97	1,20E-03
ENSDARG00000101422	hdac12	4	40	2,96	3,09E-06
ENSDARG00000013615	pbx3b	89	724	2,96	4,32E-12
ENSDARG00000034107	actl6b	21	178	2,95	1,61E-07
ENSDARG00000012694	c3a.1	2	32	2,95	2,87E-03
ENSDARG00000069752	ckba	24	234	2,94	1,35E-04
ENSDARG00000058206	si:ch211-153b23.5	18	291	2,93	2,37E-03
ENSDARG00000043843	akap7	2	19	2,92	3,12E-04
ENSDARG00000062720	pcdh1a	5	45	2,92	1,01E-06
ENSDARG00000095180	abr	6	56	2,92	2,44E-06
ENSDARG00000014420	elavl3	5	53	2,92	3,95E-04
ENSDARG00000016088	rtn2a	2	25	2,92	2,40E-04
ENSDARG00000053091	si:ch211-204c21.1	17	147	2,91	3,68E-06
ENSDARG00000087359	c3a.2	11	153	2,91	4,90E-03
ENSDARG00000003989	crhr1	7	55	2,91	2,44E-08
ENSDARG00000078123	slitrk3a	9	113	2,91	1,26E-03
ENSDARG00000094860	gpr186	5	51	2,90	2,81E-05
ENSDARG00000039828	zp3b	27	207	2,88	4,07E-11
ENSDARG00000075928	hivep3a	28	222	2,88	9,18E-09
ENSDARG00000062864	gk5	13	104	2,87	5,96E-07
ENSDARG00000033201	zgc:101840	11	98	2,87	7,84E-05
ENSDARG00000045834	si:dkey-14d8.7	175	1653	2,87	3,61E-04
ENSDARG00000074866	ptpn5	59	443	2,87	1,21E-13
ENSDARG00000071116	eepd1	13	111	2,86	3,59E-06
ENSDARG00000069928	prkceb	77	571	2,85	1,44E-13
ENSDARG00000027290	nrp1b	58	456	2,85	8,87E-08
ENSDARG00000007398	lrrk1	38	304	2,85	8,60E-07
ENSDARG00000009677	dlg1	252	1892	2,83	8,90E-10
ENSDARG00000075800	calml4a	12	104	2,81	3,95E-04
ENSDARG00000103396	znf804b	3	24	2,81	8,24E-05
ENSDARG00000034105	mtus1b	77	582	2,78	7,29E-07
ENSDARG00000091548	stard9	7	53	2,77	5,44E-05
ENSDARG00000078238	nacc1b	3	27	2,77	3,58E-05
ENSDARG00000059956	ptk6b	10	89	2,77	1,05E-03
ENSDARG00000063407	ADAMTSL4	11	85	2,76	8,73E-07
ENSDARG00000037030	casz1	10	78	2,76	5,37E-06
ENSDARG00000077710	nlgn1	29	208	2,75	2,96E-09
ENSDARG00000089382	zgc:158463	336	2919	2,75	7,10E-04
ENSDARG00000089640	si:dkey-117n7.5	5	38	2,75	7,15E-05
ENSDARG00000105184	nbeab	17	133	2,75	8,37E-06

ENSDARG00000006093	cdk15	27	194	2,74	3,26E-07
ENSDARG00000032799	kcnd2	47	340	2,74	8,81E-08
ENSDARG00000098257	pcdh15a	33	232	2,72	3,57E-08
ENSDARG00000011618	slc26a2	7	57	2,72	6,70E-05
ENSDARG00000101629	CABZ01079192.1	2	24	2,72	1,16E-03
ENSDARG00000026098	si:dkey-13a21.4	28	195	2,71	1,93E-08
ENSDARG00000102594	zgc:194627	2	28	2,71	3,05E-02
ENSDARG00000114762	CABZ01084131.1	9	65	2,71	3,67E-08
ENSDARG00000044688	dusp4	18	129	2,71	3,69E-08
ENSDARG00000096327	cd164l2	23	174	2,71	2,04E-05
ENSDARG00000013721	g6pca.2	11	86	2,70	2,67E-05
ENSDARG00000045442	cpb1	18	139	2,69	1,67E-04
ENSDARG00000075793	prex1	34	245	2,69	1,57E-06
ENSDARG00000062693	nrxn3b	13	95	2,69	3,49E-06
ENSDARG00000051923	ccnb1	2	24	2,69	1,44E-02
ENSDARG00000089856	cita	4	27	2,67	6,99E-05
ENSDARG00000074184	arhgap32b	66	437	2,67	1,19E-09
ENSDARG00000035018	thy1	14	98	2,66	1,18E-07
ENSDARG00000063150	nrxn2b	33	220	2,66	3,75E-09
ENSDARG00000004246	slit2	41	270	2,66	1,31E-09
ENSDARG00000023062	cyr61	16	114	2,66	6,63E-06
ENSDARG00000068432	si:dkey-56m19.5	17	115	2,65	2,37E-06
ENSDARG00000003200	foxm1	2	20	2,64	1,61E-02
ENSDARG00000103754	aspm	2	26	2,64	3,95E-02
ENSDARG00000062590	pleca	414	2654	2,63	3,57E-11
ENSDARG00000000606	dnah6	5	41	2,62	1,43E-03
ENSDARG00000071021	papss2a	4	30	2,62	8,37E-06
ENSDARG00000011027	fgfr1a	68	436	2,60	5,75E-08
ENSDARG00000089009	frmpd1b	63	404	2,60	2,54E-08
ENSDARG00000029439	atp2a2a	182	1158	2,59	2,44E-08
ENSDARG00000087804	tnfrsf11a	5	43	2,58	9,19E-03
ENSDARG00000102153	nrp1a	59	367	2,57	7,19E-09
ENSDARG00000061976	sema6bb	26	166	2,56	5,39E-07
ENSDARG00000053431	si:ch211-257p13.3	17	124	2,56	4,85E-04
ENSDARG00000103071	si:ch211-244c8.4	8	55	2,56	3,47E-05
ENSDARG00000071493	olfm3a	28	207	2,54	2,25E-03
ENSDARG00000087753	CR388166.1	3	23	2,54	2,31E-03
ENSDARG00000071042	cx28.8	26	163	2,54	7,95E-06
ENSDARG00000089444	omgb	4	31	2,54	1,15E-03
ENSDARG00000098925	prdm1b	18	120	2,53	2,38E-04
ENSDARG00000075491	si:ch211-26b3.4	11	67	2,52	7,86E-09
ENSDARG00000037062	dhh	2	19	2,52	4,84E-03
ENSDARG00000074863	si:dkey-112m2.1	2	25	2,50	1,60E-02
ENSDARG00000053684	aldob	4741	32356	2,50	8,84E-04
ENSDARG00000040982	fgf14	11	66	2,49	1,41E-07
ENSDARG00000079962	doc2d	86	528	2,49	6,98E-06
ENSDARG00000026165	col11a1a	7	56	2,49	5,53E-03
ENSDARG00000033327	unc5b	12	76	2,48	7,26E-05
ENSDARG00000069692	col7a1l	18	111	2,48	8,98E-05
ENSDARG00000043137	cdca8	3	26	2,47	4,85E-02
ENSDARG00000039453	dhrs7ca	8	56	2,46	2,31E-03
ENSDARG00000097652	si:dkey-234i14.2	26	162	2,46	2,33E-04
ENSDARG00000076174	trim2b	11	66	2,45	1,25E-05
ENSDARG00000019396	rergla	2	19	2,45	1,25E-02
ENSDARG00000087094	foxa	21	127	2,44	4,30E-06
ENSDARG00000004169	stmn1a	17	165	2,44	3,99E-02
ENSDARG00000014804	cacna2d1a	3	19	2,44	1,12E-03
ENSDARG00000002403	nusap1	5	37	2,44	1,87E-02
ENSDARG00000078888	iqgap1	32	198	2,43	1,86E-04
ENSDARG00000098191	si:dkey-16l2.20	3	21	2,43	1,70E-02
ENSDARG00000001559	csmd2	11	67	2,43	1,01E-04
ENSDARG00000041006	st3gal4	75	429	2,43	1,29E-07
ENSDARG00000042833	rffl	82	458	2,43	1,75E-09
ENSDARG00000104732	mlycd	7	42	2,42	1,77E-05
ENSDARG00000075718	rpz5	6	47	2,42	6,65E-03

ENSDARG00000078908	fbxo41	8	45	2,41	1,35E-07
ENSDARG00000071498	fhl1a	4	34	2,41	1,72E-02
ENSDARG00000011824	pbxip1b	522	2814	2,40	1,82E-12
ENSDARG00000062325	shank2	17	96	2,40	3,85E-08
ENSDARG00000079771	pea15	13	76	2,39	1,44E-05
ENSDARG00000069946	itga6b	553	3195	2,39	4,06E-05
ENSDARG00000012968	rhoub	15	94	2,39	1,78E-03
ENSDARG00000062045	il1rapl1a	96	514	2,37	3,23E-10
ENSDARG00000102824	pcdh10b	3	21	2,37	9,22E-04
ENSDARG00000079479	gpr4	30	167	2,37	3,45E-05
ENSDARG00000098029	si:ch211-129o18.4	11	65	2,37	1,10E-04
ENSDARG00000116871	BX284638.1	3	21	2,36	1,00E-03
ENSDARG00000061196	emilin2a	2	18	2,36	3,15E-02
ENSDARG00000024488	top2a	7	58	2,36	4,36E-02
ENSDARG00000100498	CABZ01079251.1	34	180	2,35	6,21E-08
ENSDARG00000077073	tanc1a	5	35	2,35	2,60E-03
ENSDARG00000076496	frmd4bb	18	95	2,34	1,03E-06
ENSDARG00000102584	cpne7	22	134	2,33	2,60E-03
ENSDARG00000020929	fam49ba	5	26	2,32	1,68E-04
ENSDARG00000060243	enox1	24	128	2,32	6,39E-07
ENSDARG00000090230	tmem108	5	25	2,31	2,23E-04
ENSDARG00000006456	pdgfrl	189	1015	2,30	2,95E-05
ENSDARG00000024365	crlf1a	14	74	2,30	6,79E-06
ENSDARG00000033683	tpma	3	20	2,30	1,47E-02
ENSDARG00000001634	kirrel1a	8	41	2,30	8,80E-06
ENSDARG00000038066	kpna2	17	122	2,30	3,30E-02
ENSDARG00000039657	fank1	4	28	2,30	1,28E-02
ENSDARG00000103503	dgat1a	18	94	2,30	5,97E-08
ENSDARG00000069111	IKZF2	3	19	2,30	1,43E-03
ENSDARG00000006832	galnt9	5	27	2,29	1,81E-03
ENSDARG00000070656	si:ch211-69g19.2	5	34	2,29	4,06E-02
ENSDARG00000099038	kcng1	8	45	2,29	3,24E-03
ENSDARG00000028507	itgb4	28	152	2,28	1,42E-04
ENSDARG00000091956	tmem184bb	4	22	2,28	1,48E-03
ENSDARG00000104295	arhgap21a	56	277	2,28	3,45E-10
ENSDARG00000069407	zgc:194990	421	2162	2,27	3,50E-06
ENSDARG00000088862	stxbp6	83	427	2,27	1,12E-05
ENSDARG00000033655	stmn1b	163	880	2,25	8,68E-04
ENSDARG00000001803	kcnh6a	158	770	2,25	2,29E-10
ENSDARG00000018653	acot7	3	18	2,25	1,93E-03
ENSDARG00000036107	txnipa	1330	6702	2,24	6,20E-06
ENSDARG00000034168	serpini1	63	315	2,24	5,65E-06
ENSDARG00000100307	adamts5	4	23	2,24	8,45E-05
ENSDARG00000055752	npas4a	159	814	2,24	6,94E-05
ENSDARG00000095458	si:dkey-240h12.3	70	363	2,24	1,36E-04
ENSDARG00000101911	si:dkeyp-86d6.2	8	42	2,23	1,25E-04
ENSDARG00000077367	ntng2a	17	96	2,23	2,25E-03
ENSDARG00000100313	pip5k1cb	33	165	2,22	4,26E-05
ENSDARG00000087704	gfra3	9	49	2,21	7,79E-03
ENSDARG00000059809	caska	16	77	2,21	1,46E-06
ENSDARG00000037640	aurkb	5	36	2,19	4,09E-02
ENSDARG00000016454	esr2a	15	75	2,19	5,62E-04
ENSDARG00000057833	gulp1b	5	35	2,18	1,28E-02
ENSDARG00000103679	bmp16	3	23	2,17	1,84E-02
ENSDARG00000092169	si:dkey-250k15.4	5	26	2,17	2,48E-03
ENSDARG00000026482	arhgap12b	151	697	2,17	6,70E-09
ENSDARG00000071823	adarb2	17	88	2,16	2,98E-03
ENSDARG00000016139	mkks	5	24	2,16	2,34E-04
ENSDARG00000017038	zgc:152670	4	22	2,16	4,32E-03
ENSDARG00000077761	nlgn4b	22	115	2,16	2,56E-03
ENSDARG00000013310	map3k15	7	50	2,15	1,31E-02
ENSDARG00000076534	si:ch211-14a17.10	9	42	2,15	4,83E-05
ENSDARG00000014646	aoc2	285	1529	2,15	7,52E-03
ENSDARG00000043746	nrxn3a	64	294	2,15	1,15E-07
ENSDARG00000079850	dchs1b	4	21	2,15	2,15E-02

ENSDARG00000058117	snap25b	163	763	2,14	1,59E-05
ENSDARG00000045568	bcat1	65	300	2,14	2,09E-06
ENSDARG00000116442	FAM46A	158	753	2,14	1,10E-04
ENSDARG00000007149	l1cama	46	223	2,13	2,87E-04
ENSDARG00000075562	si:ch211-119o8.4	8	38	2,12	1,01E-03
ENSDARG00000045878	nudt4b	19	90	2,12	1,71E-04
ENSDARG00000007856	rtn4rl1a	7	35	2,11	3,67E-05
ENSDARG00000023578	lpp	87	429	2,11	2,95E-03
ENSDARG00000060113	znf395a	333	1517	2,11	1,10E-05
ENSDARG00000068468	dnah3	4	22	2,11	3,30E-02
ENSDARG00000035895	fndc5b	175	803	2,10	5,46E-05
ENSDARG00000100019	arhgap11a	4	21	2,10	1,80E-02
ENSDARG00000104326	si:dkey-183i3.9	213	979	2,10	6,73E-05
ENSDARG00000055592	capn2b	4	19	2,09	8,54E-03
ENSDARG00000068461	zgc:162939	128	562	2,09	2,41E-08
ENSDARG00000062056	elmod1	44	191	2,09	5,43E-09
ENSDARG00000101812	zgc:114120	5	24	2,08	4,69E-03
ENSDARG00000075621	birc5a	4	21	2,08	3,36E-02
ENSDARG00000074443	gas7a	29	141	2,08	2,81E-03
ENSDARG00000103746	mapkbp1	11	48	2,08	1,89E-05
ENSDARG00000042151	skia	15	66	2,08	1,46E-05
ENSDARG00000091726	aebp1	2	26	2,08	7,95E-03
ENSDARG00000071375	celf5a	60	258	2,07	9,13E-08
ENSDARG00000061899	iqub	6	34	2,07	4,67E-02
ENSDARG00000063007	apc2	65	284	2,06	8,99E-08
ENSDARG00000087916	tmem240a	23	102	2,06	2,21E-08
ENSDARG00000030411	crygn2	5	22	2,06	7,28E-04
ENSDARG00000058666	dennd2da	28	126	2,06	2,29E-04
ENSDARG00000054177	adgra1a	10	44	2,04	1,08E-03
ENSDARG00000014302	brinp2	34	150	2,04	1,26E-04
ENSDARG00000097691	pclaf	4	18	2,04	1,09E-03
ENSDARG00000102975	nsg2	652	2776	2,04	6,38E-07
ENSDARG00000073952	slc4a7	131	562	2,03	6,55E-06
ENSDARG00000016868	rhobtb2a	132	560	2,03	3,43E-07
ENSDARG00000090035	rtn4r	32	140	2,03	1,69E-04
ENSDARG00000044167	padi2	35	148	2,03	7,53E-08
ENSDARG00000021806	zfp36l2	30	134	2,02	6,45E-04
ENSDARG00000020702	odf2b	6	26	2,02	8,02E-04
ENSDARG00000104296	zgc:165508	69	304	2,02	2,34E-04
ENSDARG00000018149	slc38a4	11	50	2,02	5,00E-03
ENSDARG00000010654	arhgap42b	39	165	2,02	2,95E-05
ENSDARG00000114589	jam3a	158	655	2,01	3,02E-08
ENSDARG00000004451	tnfrsfa	24	104	2,01	5,59E-04
ENSDARG00000045371	prdm14	10	46	2,01	9,18E-03
ENSDARG00000079622	frmd5	5	22	2,01	7,67E-03
ENSDARG00000025034	syngr3b	546	2234	2,00	2,36E-09
ENSDARG00000104713	zgc:165653	4	19	2,00	2,42E-02
ENSDARG00000086838	itga2.3	10	46	2,00	2,68E-03
ENSDARG00000068624	si:dkey-27p18.5	5	21	2,00	2,13E-03
ENSDARG00000000906	skap2	6	32	2,00	4,36E-02
ENSDARG00000016404	ptch1	10	46	1,99	4,91E-04
ENSDARG00000058369	mex3b	18	78	1,99	3,58E-05
ENSDARG00000079484	kcnt1	3	20	1,99	2,65E-02
ENSDARG00000006044	ust	7	35	1,99	3,84E-02
ENSDARG00000011407	col2a1b	11	52	1,98	1,04E-02
ENSDARG00000059746	plod1a	8	38	1,98	6,78E-03
ENSDARG00000006983	celf3b	86	350	1,98	4,14E-07
ENSDARG00000036082	tph1b	6	28	1,98	2,44E-04
ENSDARG00000091061	slc38a3b	78	332	1,97	8,46E-04
ENSDARG00000001889	tuba1a	31	127	1,97	2,83E-06
ENSDARG00000037393	slc43a1a	17	76	1,97	3,12E-03
ENSDARG00000060610	pcdh7b	8	42	1,97	4,53E-02
ENSDARG00000035715	marcksl1b	62	278	1,96	8,98E-03
ENSDARG00000046140	zgc:112038	7	30	1,96	4,08E-03
ENSDARG00000043241	arrb1	5	23	1,96	2,15E-03

ENSDARG00000009493	MAP3K13	39	164	1,96	5,10E-04
ENSDARG00000035338	tppp2	18	73	1,95	1,99E-05
ENSDARG00000088195	si:ch211-168f7.5	10	42	1,95	4,34E-04
ENSDARG00000013441	hey2	7	34	1,94	2,13E-02
ENSDARG00000070012	sesn2	90	361	1,94	3,90E-06
ENSDARG00000054911	tmsb	11	47	1,94	4,70E-03
ENSDARG00000105034	zdhhc18b	4	17	1,94	7,72E-03
ENSDARG00000059815	oaz2b	42	171	1,93	1,98E-04
ENSDARG00000059747	kcnj19a	245	985	1,93	6,70E-05
ENSDARG00000090876	si:dkey-217f16.5	5	22	1,92	6,45E-04
ENSDARG00000030107	si:ch211-207i1.2	76	302	1,91	8,77E-05
ENSDARG00000025024	pnoca	4626	18846	1,91	1,01E-03
ENSDARG00000036968	si:ch1073-416d2.3	217	893	1,91	2,04E-03
ENSDARG00000098511	ntrk2b	5	19	1,91	3,03E-03
ENSDARG00000076386	epdl1	516	2098	1,90	1,42E-03
ENSDARG00000101482	hk2	8	33	1,90	4,78E-03
ENSDARG00000105529	vgf	4	17	1,90	1,38E-02
ENSDARG00000045804	abhd2b	28	113	1,90	1,94E-03
ENSDARG00000114948	zgc:194551	6	28	1,89	3,96E-02
ENSDARG00000090646	tnk2a	73	279	1,88	2,19E-07
ENSDARG00000069430	tp53i11a	8	35	1,88	3,76E-03
ENSDARG00000103156	gpc6a	34	132	1,88	2,95E-05
ENSDARG00000102051	mtbl	384	1465	1,88	8,67E-06
ENSDARG00000103056	itga4	4	20	1,88	3,94E-02
ENSDARG00000034443	mpped2	273	1029	1,88	1,28E-07
ENSDARG00000089172	si:ch1073-391i24.1	31	122	1,87	1,42E-03
ENSDARG00000021086	znf367	11	49	1,87	2,09E-02
ENSDARG00000104841	CABZ01015475.1	8	32	1,86	2,55E-03
ENSDARG00000097855	C2orf88	7	30	1,86	2,52E-02
ENSDARG00000020956	pck2	23	86	1,85	6,18E-06
ENSDARG00000077569	lrrn3a	49	183	1,85	1,24E-05
ENSDARG00000037603	zgc:162144	10	42	1,85	4,02E-02
ENSDARG00000075158	igdcc3	20	76	1,84	1,16E-03
ENSDARG00000079166	ace	365	1440	1,84	5,50E-03
ENSDARG00000090607	si:dkeyp-117b11.1	54	216	1,83	9,01E-03
ENSDARG00000023683	cacna1fb	35	138	1,83	3,76E-03
ENSDARG00000043446	efhd1	18	72	1,82	3,00E-03
ENSDARG00000101178	sh3rf2	31	114	1,82	1,02E-04
ENSDARG00000044056	ahi1	105	378	1,82	5,32E-09
ENSDARG00000087299	armc9	26	98	1,82	2,74E-06
ENSDARG00000029431	si:ch211-147k10.5	5	23	1,82	4,90E-02
ENSDARG00000018270	olfm1a	35	129	1,82	1,29E-04
ENSDARG00000002731	sdc2	646	2319	1,81	3,69E-08
ENSDARG00000037495	rtn4rl2b	1856	7174	1,81	4,59E-03
ENSDARG00000092310	si:ch211-199g17.2	7	29	1,81	8,77E-03
ENSDARG00000100851	gphnb	105	376	1,81	7,93E-08
ENSDARG00000074149	itpr1b	214	775	1,80	1,59E-05
ENSDARG00000061600	tmem2	23	83	1,80	7,08E-06
ENSDARG00000069006	tle3b	76	271	1,80	6,38E-06
ENSDARG00000040907	gcgb	1785	7351	1,79	3,93E-02
ENSDARG00000063417	erf	80	282	1,79	5,48E-07
ENSDARG00000042368	kif26aa	18	69	1,79	1,88E-03
ENSDARG00000035859	angptl4	1126	4075	1,78	2,38E-04
ENSDARG00000073944	si:ch73-386h18.1	232	818	1,78	3,58E-07
ENSDARG00000019417	gadd45ga	1070	3960	1,78	2,26E-03
ENSDARG00000113771	GRIK2	143	502	1,77	2,81E-06
ENSDARG00000054448	kif1c	19	68	1,77	1,20E-03
ENSDARG00000067859	scospondin	6	23	1,77	1,34E-02
ENSDARG00000005464	dnase1l1	38	135	1,77	7,05E-05
ENSDARG00000060103	cpeb3	68	241	1,77	1,42E-04
ENSDARG00000079946	sqlea	8	30	1,76	3,55E-02
ENSDARG00000036446	PRKAR2B	6	22	1,76	1,64E-02
ENSDARG00000088593	chst1	22	81	1,76	2,11E-03
ENSDARG00000008186	syk	43	151	1,76	6,20E-06
ENSDARG00000052468	GSK3B (1 of many)	56	197	1,76	1,02E-04

ENSDARG00000094994	si:dkey-106c17.2	8	28	1,74	7,51E-03
ENSDARG00000007912	si:ch211-196i2.1	9	32	1,74	6,48E-03
ENSDARG00000056530	cpamd8	2	21	1,73	3,05E-02
ENSDARG00000044362	unc119b	27	93	1,73	5,94E-04
ENSDARG00000043131	BX664625.1	13	46	1,73	3,33E-03
ENSDARG00000038822	mrc1b	15	55	1,73	2,53E-02
ENSDARG00000001950	ak1	6	22	1,73	2,75E-02
ENSDARG00000028661	cntfr	31	108	1,72	1,78E-03
ENSDARG00000099096	gabrb4	35	123	1,72	3,98E-04
ENSDARG00000023302	gpbar1	36	129	1,71	1,25E-02
ENSDARG00000045803	mfge8b	1121	3813	1,71	8,71E-05
ENSDARG00000052020	cmtm4	95	321	1,71	1,32E-06
ENSDARG00000044808	slc4a4b	24	87	1,71	1,16E-02
ENSDARG00000006878	phf21aa	58	193	1,71	2,13E-08
ENSDARG00000027063	arpc1b	19	67	1,71	4,68E-03
ENSDARG00000025522	sgk1	74	269	1,71	1,33E-02
ENSDARG00000086490	si:dkey-92f12.2	14	48	1,70	9,90E-04
ENSDARG00000056797	ppp2r2ca	6	20	1,70	4,26E-03
ENSDARG00000078781	si:ch211-161c3.6	8	31	1,70	2,13E-02
ENSDARG00000017710	ppp1r14ab	15	53	1,70	2,31E-02
ENSDARG00000088100	KCNIP4	90	306	1,69	1,30E-03
ENSDARG00000071410	cks2	8	28	1,69	3,14E-02
ENSDARG00000058100	b3gnt3	169	555	1,68	4,47E-06
ENSDARG00000092199	si:ch211-250c4.4	217	755	1,68	6,85E-03
ENSDARG00000021539	ephb2b	111	365	1,68	1,77E-06
ENSDARG00000010823	memo1	188	608	1,68	1,25E-09
ENSDARG00000112961	LO017848.1	15	49	1,67	2,27E-03
ENSDARG00000075222	amer2	11	37	1,67	7,05E-03
ENSDARG00000060678	ndst2b	84	278	1,67	1,14E-04
ENSDARG00000104222	inppl1a	41	134	1,66	1,26E-04
ENSDARG00000098526	gpr75	76	250	1,66	2,39E-05
ENSDARG00000056541	nr0b1	60	206	1,66	6,42E-03
ENSDARG00000006601	kaznb	25	83	1,66	1,02E-04
ENSDARG00000059801	pdzd2	97	317	1,66	4,64E-05
ENSDARG00000095826	smdt1a	8	26	1,66	6,36E-03
ENSDARG00000017929	ncoa2	746	2399	1,65	4,04E-06
ENSDARG00000076943	lhfpl4b	32	104	1,65	5,07E-05
ENSDARG00000096637	si:dkey-11c5.11	54	178	1,65	1,30E-03
ENSDARG00000060372	plxna2	17	58	1,65	8,98E-03
ENSDARG00000098880	pclob	697	2212	1,64	1,84E-06
ENSDARG00000035918	bzw2	20	64	1,64	4,77E-04
ENSDARG00000027602	lrfn1	19	65	1,63	7,00E-03
ENSDARG00000100020	pim1	391	1238	1,63	4,09E-06
ENSDARG00000038574	scg2b	2763	8742	1,63	2,83E-06
ENSDARG00000004871	mst1rb	401	1273	1,63	2,55E-05
ENSDARG00000032369	btbd6b	433	1364	1,62	3,42E-06
ENSDARG00000093081	si:ch73-92i20.1	32	106	1,62	1,22E-02
ENSDARG00000087822	agap3	42	132	1,62	1,17E-05
ENSDARG00000099752	si:dkey-19b23.7	50	160	1,62	3,02E-04
ENSDARG00000076025	dgkzb	19	64	1,61	2,75E-02
ENSDARG00000014680	znf710a	6	22	1,60	4,56E-02
ENSDARG00000000161	itsn2a	27	83	1,60	2,82E-05
ENSDARG00000059616	hs3st2	21	69	1,60	1,57E-02
ENSDARG00000070338	hoxc4a	18	60	1,59	2,91E-02
ENSDARG00000053452	pop5	19	59	1,59	2,35E-05
ENSDARG00000061371	cdh18a	42	132	1,59	2,33E-03
ENSDARG00000099079	aclya	733	2254	1,59	9,38E-06
ENSDARG00000071009	kif20ba	86	278	1,59	7,41E-03
ENSDARG00000006522	otop2	19	61	1,59	1,33E-02
ENSDARG00000073866	tdp1	51	162	1,59	4,31E-03
ENSDARG00000022254	prkcbb	170	541	1,58	4,59E-03
ENSDARG00000014939	KCNN2	8	26	1,57	2,35E-02
ENSDARG00000055585	c1galt1a	22	70	1,57	6,82E-03
ENSDARG00000006474	saxo2	13	43	1,57	1,78E-02
ENSDARG00000027724	dgkb	31	100	1,57	8,88E-03

ENSDARG00000056156	npdc1b	191	578	1,57	1,67E-06
ENSDARG00000091342	adcy5	170	512	1,57	4,02E-06
ENSDARG00000098852	smdt1b	63	198	1,57	6,48E-03
ENSDARG00000088083	sik3	66	210	1,56	1,06E-02
ENSDARG00000069185	celsr1a	130	389	1,56	1,84E-07
ENSDARG00000090965	ripk3	13	41	1,56	4,67E-03
ENSDARG00000076998	si:ch73-92i20.1	28	88	1,55	8,75E-03
ENSDARG00000041616	b3gnt9	10	33	1,55	2,53E-02
ENSDARG00000058032	si:dkey-6n6.2	22	69	1,54	1,25E-02
ENSDARG00000071465	lrrc39	7	20	1,54	1,12E-02
ENSDARG00000077536	snrnp200	458	1356	1,54	9,38E-06
ENSDARG00000009607	ccnc	366	1085	1,54	6,18E-06
ENSDARG00000027183	namptb	51	153	1,54	6,88E-04
ENSDARG00000059202	tspan2b	13	40	1,53	1,61E-02
ENSDARG00000101606	rims2a	2830	8484	1,53	8,16E-04
ENSDARG00000114503	phtf2	28	83	1,51	2,82E-03
ENSDARG00000056572	si:dkey-261i16.5	9	30	1,51	2,65E-02
ENSDARG00000079741	CSRNP3	22	68	1,51	3,69E-03
ENSDARG00000018750	npr1b	20	61	1,50	3,34E-02
ENSDARG00000010267	dpydb	47	139	1,50	1,87E-03
ENSDARG00000012914	arhgef4	79	229	1,50	1,34E-04
ENSDARG00000012078	meis1b	693	2032	1,50	7,79E-04
ENSDARG00000090326	MAPK8IP1 (1 of ma	55	161	1,50	1,03E-03
ENSDARG00000063646	lnpa	23	65	1,49	7,91E-05
ENSDARG00000102899	cremb	43	131	1,48	4,05E-02
ENSDARG00000105083	chd5	212	603	1,48	6,10E-07
ENSDARG00000075850	gnal2	207	602	1,48	2,55E-03
ENSDARG00000028322	pex14	214	605	1,48	7,79E-08
ENSDARG00000027423	igf1ra	82	240	1,48	3,41E-03
ENSDARG00000034424	atp1b2b	10	31	1,47	3,01E-02
ENSDARG00000008912	slc8a2a	17	48	1,47	6,37E-05
ENSDARG00000027807	frk	7	20	1,46	4,72E-02
ENSDARG00000058646	ptprna	3010	8419	1,46	2,45E-06
ENSDARG00000091211	adh8a	18	51	1,46	1,97E-02
ENSDARG00000014053	olfm1b	1131	3258	1,46	5,87E-03
ENSDARG00000059729	sec14l8	57	164	1,45	4,21E-03
ENSDARG00000011600	epha4b	212	596	1,45	3,76E-04
ENSDARG00000033950	lamb2l	49	139	1,45	3,80E-03
ENSDARG00000023448	galnt14	98	276	1,45	8,16E-04
ENSDARG00000005626	RHOU (1 of many)	35	98	1,45	2,13E-06
ENSDARG00000075188	adamts10	10	28	1,45	3,19E-02
ENSDARG00000027822	tmtops2b	10	29	1,44	1,42E-02
ENSDARG00000059587	CU928117.1	28	79	1,44	1,64E-03
ENSDARG00000074828	si:dkey-263f15.2	9	26	1,44	3,94E-02
ENSDARG00000016429	farp2	94	263	1,44	1,75E-03
ENSDARG00000112644	CU459056.1	113	309	1,43	2,92E-05
ENSDARG00000013841	abl2	92	255	1,43	5,84E-04
ENSDARG00000045301	st8sia3	27	77	1,42	2,96E-03
ENSDARG00000020080	ek1	394	1120	1,42	2,29E-02
ENSDARG00000063649	tead3b	33	91	1,42	8,23E-04
ENSDARG00000104574	pcsk6	92	261	1,41	2,89E-02
ENSDARG00000015002	cdh4	106	287	1,41	6,72E-05
ENSDARG00000075156	tbc1d4	53	146	1,41	1,85E-03
ENSDARG00000061255	dusp3a	41	114	1,41	3,54E-03
ENSDARG00000104701	map7d1b	62	168	1,40	3,06E-05
ENSDARG00000061096	dapk2a	67	187	1,40	1,17E-02
ENSDARG00000069619	atf7ip	281	747	1,40	3,75E-09
ENSDARG00000102908	il16	6	17	1,40	1,80E-02
ENSDARG00000010610	sarm1	34	95	1,39	2,23E-02
ENSDARG00000095011	si:ch211-67n3.9	25	67	1,39	1,22E-04
ENSDARG00000022858	wnt7ba	8	23	1,39	4,20E-02
ENSDARG00000098368	aplp1	53	144	1,39	6,85E-03
ENSDARG00000095457	si:ch211-147d7.5	28	77	1,38	2,79E-02
ENSDARG00000061121	adgrl3.1	138	366	1,38	3,21E-07
ENSDARG00000018961	gtpbp4	1020	2711	1,38	1,02E-04

ENSDARG00000018773	hivep2b	9	25	1,38	4,67E-02
ENSDARG00000062915	polr3b	46	121	1,37	1,01E-04
ENSDARG00000100422	ptpro	10	27	1,37	2,69E-02
ENSDARG00000075024	si:ch73-375g18.1	100	271	1,37	9,94E-03
ENSDARG00000075110	dab2ipb	398	1047	1,37	6,11E-05
ENSDARG00000087013	cubn	6	16	1,37	3,64E-02
ENSDARG00000057032	ppm1nb	37	97	1,37	8,03E-04
ENSDARG00000087616	maptb	52	140	1,37	4,69E-03
ENSDARG00000058421	lgi1b	73	194	1,37	1,39E-03
ENSDARG00000074447	dapk3	84	219	1,36	1,68E-05
ENSDARG00000078348	tcf20	361	938	1,36	1,77E-06
ENSDARG00000079468	rhobtb1	28	76	1,36	6,51E-03
ENSDARG00000090185	si:dkeyp-73b11.8	16	43	1,36	2,92E-02
ENSDARG00000076618	tm2d3	107	280	1,36	8,11E-05
ENSDARG00000077134	gpr158a	629	1676	1,35	5,65E-03
ENSDARG00000020387	nipsnap3a	101	267	1,35	4,33E-03
ENSDARG00000005343	ccdc85al	89	236	1,35	6,96E-03
ENSDARG00000035132	rgs3b	160	417	1,35	3,80E-04
ENSDARG00000008983	sh3glb2a	58	149	1,34	2,60E-04
ENSDARG00000054837	zgc:136870	51	134	1,33	1,37E-02
ENSDARG00000061093	phldb1a	41	109	1,33	1,07E-02
ENSDARG00000022176	IGLON5	77	200	1,32	2,98E-03
ENSDARG00000062080	shc2	11	29	1,32	4,85E-02
ENSDARG00000060309	srgap3	129	331	1,32	8,39E-04
ENSDARG00000062485	dock11	84	213	1,32	1,54E-04
ENSDARG00000076005	piezo2a.2	72	183	1,32	1,04E-04
ENSDARG00000003635	mogat3b	22	58	1,31	9,94E-03
ENSDARG00000013649	casr	322	820	1,31	2,13E-03
ENSDARG00000018534	slc6a9	16	43	1,31	3,68E-02
ENSDARG00000102123	phtf2	14	36	1,31	2,05E-02
ENSDARG00000067626	ywhag1	205	532	1,30	1,70E-02
ENSDARG00000036774	rgs12b	233	583	1,30	1,14E-07
ENSDARG00000021309	rhoca	59	148	1,30	9,12E-05
ENSDARG00000060184	pkn1a	99	247	1,30	9,19E-06
ENSDARG00000088590	pxna	15	38	1,30	1,31E-02
ENSDARG00000036684	ndufa7	170	431	1,29	5,34E-03
ENSDARG00000035056	fgf13a	25	65	1,29	3,90E-02
ENSDARG00000077178	zgc:152977	16	41	1,29	9,36E-03
ENSDARG00000031756	mef2aa	80	199	1,29	1,11E-03
ENSDARG00000037961	rcn3	262	657	1,29	2,13E-03
ENSDARG00000077329	nlgn2a	33	82	1,29	3,66E-03
ENSDARG00000078404	cdh26.1	12	30	1,28	3,31E-02
ENSDARG00000015731	ano5a	22	58	1,28	2,33E-02
ENSDARG00000104387	slc4a5	38	95	1,27	1,44E-02
ENSDARG00000105782	CABZ01081777.1	32	78	1,27	3,74E-04
ENSDARG00000020000	sh3bp4a	89	222	1,27	4,06E-03
ENSDARG00000023916	phactr1	34	86	1,27	3,97E-03
ENSDARG00000058606	sik1	3418	8400	1,27	1,28E-03
ENSDARG00000033599	zgc:100906	85	207	1,26	1,40E-03
ENSDARG00000071026	mllt11	176	433	1,25	5,49E-03
ENSDARG00000004251	dhfr	10	26	1,25	2,76E-02
ENSDARG00000099257	si:ch73-119p20.1	117	284	1,24	8,21E-04
ENSDARG00000104571	gadd45ab	86	209	1,24	5,20E-03
ENSDARG00000006899	far1	109	259	1,24	2,44E-05
ENSDARG00000032868	pde4ba	576	1370	1,23	1,14E-04
ENSDARG00000061732	raph1b	117	280	1,23	4,66E-04
ENSDARG00000092722	unc5da	52	126	1,23	1,35E-02
ENSDARG00000075849	prr12b	134	318	1,22	2,85E-05
ENSDARG00000055305	ret	2736	6512	1,22	1,10E-03
ENSDARG00000092489	si:ch73-103b9.2	24	57	1,22	7,73E-03
ENSDARG00000037607	nol4lb	44	105	1,22	9,07E-04
ENSDARG00000077545	si:dkey-16p21.7	15	38	1,22	5,11E-03
ENSDARG00000045549	bik	9	21	1,22	3,54E-02
ENSDARG00000036291	nucb2b	4465	10444	1,21	2,04E-06
ENSDARG00000103924	zgc:154055	37	90	1,21	1,26E-02

ENSDARG00000018179	acvrl1	13	32	1,21	3,12E-03
ENSDARG00000100452	nop53	484	1146	1,21	2,20E-03
ENSDARG00000036420	si:dkey-33c9.6	13	30	1,21	3,34E-02
ENSDARG00000014081	fam184a	190	448	1,21	1,46E-03
ENSDARG00000079295	cacna1bb	249	585	1,20	8,24E-04
ENSDARG00000096603	bmb	94	228	1,20	4,05E-02
ENSDARG00000042141	myo6b	62	148	1,20	1,03E-02
ENSDARG00000115902	lnpa	54	127	1,20	6,39E-04
ENSDARG00000101419	drp2	36	85	1,20	2,66E-02
ENSDARG00000029668	crim1	176	411	1,20	1,48E-03
ENSDARG00000031763	smad6b	110	260	1,20	6,50E-03
ENSDARG00000102340	ptn	5252	12530	1,20	2,52E-02
ENSDARG00000098897	pds5b	196	454	1,19	8,25E-05
ENSDARG00000063626	ddx21	250	586	1,19	3,35E-03
ENSDARG00000002988	tnnt2d	29	68	1,19	2,07E-02
ENSDARG00000073883	clstn3	1081	2547	1,19	1,56E-02
ENSDARG00000026871	uchl1	240	554	1,18	4,31E-04
ENSDARG00000099753	zgc:63863	47	109	1,18	1,10E-03
ENSDARG00000079908	tmem151a	11	25	1,18	9,26E-03
ENSDARG00000052748	capn1b	37	87	1,17	1,04E-02
ENSDARG00000005453	foxp2	142	327	1,17	1,68E-03
ENSDARG00000075851	oafa	85	201	1,17	4,50E-02
ENSDARG00000062797	nell2b	46	108	1,17	4,06E-02
ENSDARG00000077410	myo9b	51	116	1,17	4,29E-03
ENSDARG00000020845	tns1b	131	303	1,17	7,48E-03
ENSDARG00000017058	large2	107	246	1,17	3,21E-03
ENSDARG00000091756	insm1a	840	1910	1,17	2,23E-04
ENSDARG00000015053	grip1	28	65	1,16	3,32E-02
ENSDARG00000100524	abca3b	450	1019	1,16	1,79E-05
ENSDARG00000036505	syt4	918	2089	1,16	2,07E-03
ENSDARG00000100708	jakmip3	12	28	1,16	9,77E-03
ENSDARG00000020107	usp2a	305	687	1,16	4,27E-05
ENSDARG00000006892	myo3b	139	315	1,16	5,00E-04
ENSDARG00000029524	impdh1b	624	1402	1,16	1,34E-06
ENSDARG00000104478	cap2	55	127	1,16	1,03E-02
ENSDARG00000062934	gpr12	42	97	1,15	2,46E-02
ENSDARG00000021579	orc5	44	100	1,15	1,84E-02
ENSDARG00000005034	ispd	340	759	1,15	5,65E-06
ENSDARG00000104874	CABZ01088508.1	16	37	1,15	3,41E-03
ENSDARG00000007774	vash2	66	148	1,15	1,09E-03
ENSDARG00000002127	stam	283	630	1,14	1,56E-06
ENSDARG00000078676	myrf	42	96	1,14	3,03E-02
ENSDARG00000028000	pfkpa	172	383	1,14	7,21E-04
ENSDARG00000100947	mafk	82	184	1,13	7,14E-03
ENSDARG00000009693	llgl1	74	168	1,13	1,62E-02
ENSDARG00000009242	fev	343	762	1,13	3,00E-03
ENSDARG00000036329	ndufa1	732	1621	1,13	3,52E-04
ENSDARG00000103390	cacna2d2a	348	779	1,13	7,58E-03
ENSDARG00000021846	hid1a	208	460	1,13	2,51E-04
ENSDARG00000007171	aig1	51	114	1,12	4,95E-03
ENSDARG00000061994	acacb	80	179	1,12	1,01E-02
ENSDARG00000069995	dpy19l1l	61	136	1,12	2,84E-02
ENSDARG00000043661	cadpsa	130	288	1,12	9,55E-04
ENSDARG00000037403	si:dkey-4p15.3	236	530	1,12	2,71E-02
ENSDARG00000060070	adcy7	129	283	1,12	3,01E-04
ENSDARG00000063433	atp2b2	181	407	1,12	2,24E-02
ENSDARG00000104014	mark1	28	63	1,12	8,16E-03
ENSDARG00000057007	ctbp1	1036	2272	1,12	8,16E-05
ENSDARG00000100170	pam	6467	14195	1,12	1,58E-04
ENSDARG00000017798	bcor	208	457	1,11	4,77E-04
ENSDARG00000031768	roraa	156	346	1,11	1,30E-02
ENSDARG00000014655	itpr1a	35	77	1,11	8,71E-03
ENSDARG00000090213	rheb	145	314	1,10	1,75E-05
ENSDARG00000063910	mt-atp8	271	587	1,10	2,51E-04
ENSDARG00000061272	BX294434.1	29	64	1,10	1,02E-02

ENSDARG00000069467	igsf9bb	28	61	1,10	1,68E-02
ENSDARG00000104339	SMARCA4	42	92	1,09	3,00E-02
ENSDARG00000055874	cpe	11521	24810	1,09	3,07E-04
ENSDARG00000101844	mibp2	1644	3551	1,09	1,61E-03
ENSDARG00000075437	shc1	127	271	1,09	2,70E-05
ENSDARG00000102380	cnnm4a	38	83	1,08	3,12E-02
ENSDARG00000039346	ndufa5	357	768	1,08	2,08E-03
ENSDARG00000069940	ppap2d	153	327	1,08	1,88E-03
ENSDARG00000036337	cers3b	27	59	1,08	2,02E-02
ENSDARG00000078078	lrfn4b	38	83	1,08	3,92E-02
ENSDARG00000037593	prickle2b	237	508	1,08	1,12E-03
ENSDARG00000087843	cntn1a	31	67	1,08	3,42E-02
ENSDARG00000078169	cacna2d2b	155	335	1,08	8,16E-03
ENSDARG00000069318	efr3bb	66	143	1,08	7,17E-04
ENSDARG00000071673	ctdspla	101	215	1,07	7,99E-05
ENSDARG00000008026	fam129bb	75	162	1,07	1,35E-02
ENSDARG00000042545	sema3ga	35	76	1,07	1,50E-02
ENSDARG00000043055	hiat1b	318	673	1,07	2,42E-05
ENSDARG00000056262	slc35g2a	505	1074	1,07	1,38E-03
ENSDARG00000110357	irf1b	465	992	1,07	5,11E-03
ENSDARG00000007886	slc35b2	157	333	1,07	9,69E-07
ENSDARG00000104347	rtn4b	40	84	1,06	6,11E-05
ENSDARG00000014017	rrm1	30	63	1,06	1,56E-02
ENSDARG00000021984	ndufa2	252	535	1,06	6,97E-03
ENSDARG00000087018	FO704748.1	25	54	1,06	1,36E-02
ENSDARG00000036903	irak1bp1	23	50	1,06	2,71E-02
ENSDARG00000077145	adcy3a	54	115	1,05	2,67E-02
ENSDARG00000101979	eps8l3a	57	121	1,05	9,17E-03
ENSDARG00000023724	cdc42se1	152	321	1,05	3,89E-02
ENSDARG00000046022	tmed7	1068	2228	1,04	1,67E-03
ENSDARG00000102118	slf1	32	68	1,04	4,75E-03
ENSDARG00000012593	col5a1	57	120	1,04	1,03E-02
ENSDARG00000104538	tmem184a	288	600	1,04	2,08E-03
ENSDARG00000074604	phc3	60	127	1,04	2,73E-02
ENSDARG00000059789	parp8	193	398	1,03	4,79E-05
ENSDARG00000043130	notch2	55	114	1,03	7,55E-03
ENSDARG00000032985	mrpl27	118	246	1,03	2,02E-02
ENSDARG00000007614	wwox	45	93	1,03	7,98E-04
ENSDARG00000005359	sucla2	684	1399	1,03	1,17E-05
ENSDARG00000055502	cicb	238	489	1,02	2,99E-04
ENSDARG00000006923	cacna1ab	167	347	1,02	2,82E-02
ENSDARG00000069970	lingo2b	107	219	1,02	1,78E-03
ENSDARG00000056105	syt11b	60	122	1,02	4,04E-03
ENSDARG00000100371	eef2b	8130	16683	1,01	1,07E-02
ENSDARG00000030756	dnmt1	30	61	1,01	2,11E-02
ENSDARG00000099499	ndufa4l	1066	2169	1,01	5,76E-04
ENSDARG00000079244	pigw	35	71	1,00	4,44E-02
ENSDARG00000042484	tle2a	139	282	1,00	2,49E-04
ENSDARG00000029296	nexmifb	571	282	-1,01	1,46E-04
ENSDARG00000016623	si:ch211-195b13.1	2181	1070	-1,01	1,08E-02
ENSDARG00000031075	cadm1a	277	135	-1,01	4,21E-02
ENSDARG00000115425	CABZ01038524.1	56	28	-1,01	1,21E-02
ENSDARG00000063370	sgk2a	1000	494	-1,01	2,65E-07
ENSDARG00000018153	nt5c1ba	41	19	-1,01	4,85E-02
ENSDARG00000103848	apba1a	81	39	-1,01	2,67E-02
ENSDARG00000059857	blvra	190	93	-1,01	2,88E-03
ENSDARG00000034222	hs1bp3	98	47	-1,02	1,58E-02
ENSDARG00000075564	fam13a	669	326	-1,02	2,20E-03
ENSDARG00000056483	ssbp4	527	259	-1,02	1,11E-04
ENSDARG00000005049	rab20	134	66	-1,02	1,95E-03
ENSDARG00000042005	elp3	381	185	-1,02	8,98E-05
ENSDARG00000061021	fam160a1a	118	56	-1,03	8,12E-03
ENSDARG00000028957	maff	657	320	-1,03	4,21E-04
ENSDARG00000101725	parietopsin	51	26	-1,03	4,69E-02
ENSDARG00000103743	si:dkeyp-72e1.9	168	80	-1,03	4,11E-02

ENSDARG00000029472	afdna	952	459	-1,04	2,51E-03
ENSDARG00000052074	si:ch211-122f10.4	145	69	-1,04	1,83E-03
ENSDARG00000043710	parvaa	74	36	-1,04	3,87E-02
ENSDARG00000077138	zgc:195173	532	254	-1,04	1,15E-02
ENSDARG00000015822	sesn3	1943	932	-1,04	2,13E-03
ENSDARG00000103542	vegfaa	1139	547	-1,04	2,45E-04
ENSDARG00000102424	si:ch211-114m9.1	258	124	-1,04	1,58E-03
ENSDARG00000060366	slc12a9	306	147	-1,04	6,93E-04
ENSDARG00000112923	CABZ01088036.1	186	89	-1,05	4,74E-03
ENSDARG00000041108	ctsh	543	259	-1,05	1,72E-05
ENSDARG00000098108	dusp2	12763	6048	-1,05	1,39E-02
ENSDARG00000057378	selenou1a	232	111	-1,05	3,19E-05
ENSDARG00000053474	tdrkh	142	67	-1,05	7,98E-04
ENSDARG00000062831	si:ch73-22o12.1	891	425	-1,06	1,98E-04
ENSDARG00000087048	si:dkey-165n16.1	241	113	-1,06	2,76E-02
ENSDARG00000036058	gnao1b	949	450	-1,06	2,35E-03
ENSDARG00000037894	ezh1	72	33	-1,06	3,94E-02
ENSDARG00000002037	pfkfb2b	349	165	-1,06	2,83E-04
ENSDARG00000101175	mcu	512	239	-1,07	2,86E-02
ENSDARG00000039422	fuom	90	42	-1,07	3,75E-02
ENSDARG00000007818	palm1b	403	192	-1,07	1,43E-06
ENSDARG00000061817	kif1aa	188	86	-1,07	2,95E-02
ENSDARG00000024828	tmem134	444	210	-1,07	1,57E-06
ENSDARG00000042846	disp2	2305	1081	-1,07	2,52E-03
ENSDARG00000053119	smpdl3a	240	112	-1,07	8,17E-03
ENSDARG00000029751	wbp1lb	431	200	-1,08	1,03E-02
ENSDARG00000042620	gstr	1194	560	-1,08	1,19E-04
ENSDARG00000016745	slc35f6	660	307	-1,09	1,56E-03
ENSDARG00000074071	mrnip	108	50	-1,09	4,29E-02
ENSDARG00000006257	vldlr	969	452	-1,09	2,60E-03
ENSDARG00000112454	FO744833.2	51	24	-1,09	1,39E-02
ENSDARG00000075253	pik3cb	173	80	-1,09	1,60E-02
ENSDARG00000026680	slc25a14	914	424	-1,09	1,04E-04
ENSDARG00000103522	si:zfos-943e10.1	91	41	-1,10	2,79E-02
ENSDARG00000020761	arrdc2	1445	661	-1,10	1,10E-02
ENSDARG00000001303	psmb8a	350	161	-1,10	6,82E-04
ENSDARG00000102684	lrrc1	105	48	-1,10	8,94E-03
ENSDARG00000079530	si:dkey-17m8.1	266	123	-1,10	8,30E-05
ENSDARG00000011042	pgbd5	424	192	-1,10	1,76E-02
ENSDARG00000010794	tfeb	887	409	-1,11	1,02E-04
ENSDARG00000099313	naga	298	136	-1,11	7,71E-04
ENSDARG00000011157	arhgef18a	435	198	-1,11	4,91E-03
ENSDARG00000055115	dtnbp1a	405	185	-1,12	4,57E-06
ENSDARG00000042630	hebp2	447	203	-1,12	2,52E-03
ENSDARG00000059836	ddit3	1599	721	-1,12	1,63E-02
ENSDARG00000043081	ctsz	237	108	-1,12	1,09E-04
ENSDARG00000026406	anxa5a	614	276	-1,12	1,10E-02
ENSDARG00000035652	sat1a.1	585	259	-1,13	4,94E-02
ENSDARG00000007760	spg21	654	297	-1,13	2,77E-05
ENSDARG00000063509	lrrc58b	126	57	-1,13	2,35E-03
ENSDARG00000031817	trim2a	352	158	-1,13	2,71E-03
ENSDARG00000024785	ctnna2	418	189	-1,13	9,98E-04
ENSDARG00000061918	cplx2	1361	609	-1,13	1,25E-02
ENSDARG00000076010	twist1b	186	83	-1,13	3,95E-02
ENSDARG00000059906	sdc4	2173	977	-1,13	7,94E-04
ENSDARG00000076779	fam13b	3664	1655	-1,14	9,36E-06
ENSDARG00000062054	cpt1ab	788	349	-1,14	1,39E-02
ENSDARG00000058538	alcamb	4366	1905	-1,14	3,29E-02
ENSDARG00000074414	sema4ba	279	125	-1,14	5,74E-04
ENSDARG00000038025	cbx7a	1104	488	-1,14	1,52E-02
ENSDARG00000094578	epsti1	64	29	-1,14	1,80E-02
ENSDARG00000043899	psma6l	59	26	-1,14	7,19E-03
ENSDARG00000044073	fuca2	807	363	-1,14	2,09E-06
ENSDARG00000092667	si:ch211-271e10.2	155	69	-1,14	7,01E-03
ENSDARG00000087413	bean1	396	174	-1,14	9,16E-03

ENSDARG00000075264	FAM124A	228	101	-1,15	3,18E-04
ENSDARG00000002656	stxbp5a	555	243	-1,15	1,85E-02
ENSDARG00000062135	sytl5	186	82	-1,15	6,07E-03
ENSDARG00000099646	pcdh1g3	86	38	-1,15	2,87E-03
ENSDARG00000058692	fzd3b	127	56	-1,15	2,35E-02
ENSDARG00000018921	fam84a	486	212	-1,15	1,75E-02
ENSDARG00000019532	fads2	92	40	-1,16	2,75E-02
ENSDARG00000014914	lamp2	2394	1054	-1,16	3,44E-03
ENSDARG00000069302	snx9b	1083	483	-1,16	5,71E-06
ENSDARG00000069951	eef1a1b	540	234	-1,16	3,31E-02
ENSDARG00000053298	nkx2.2a	1153	511	-1,16	9,06E-06
ENSDARG00000095754	si:dkey-234h16.7	199	87	-1,16	3,29E-03
ENSDARG00000052658	pptc7b	151	66	-1,16	4,18E-03
ENSDARG00000017105	vaspb	340	149	-1,17	1,32E-06
ENSDARG00000100280	znfx1	70	29	-1,17	3,22E-02
ENSDARG00000006031	abat	1119	496	-1,17	1,00E-06
ENSDARG00000031776	zgc:92066	4090	1790	-1,17	1,29E-03
ENSDARG00000011376	dnase1l4.2	334	145	-1,17	1,54E-03
ENSDARG00000032856	ccdc120	107	46	-1,18	4,53E-03
ENSDARG00000101446	PCP4L1	265	115	-1,18	7,07E-04
ENSDARG00000037196	arid5b	342	149	-1,18	4,38E-04
ENSDARG00000043211	ripk4	159	69	-1,18	2,20E-03
ENSDARG00000068981	glceb	455	199	-1,18	1,26E-05
ENSDARG00000110950	CU929544.1	160	68	-1,18	4,21E-02
ENSDARG00000026655	tspo	468	202	-1,18	4,00E-03
ENSDARG00000009390	npl	513	222	-1,18	3,19E-03
ENSDARG00000100822	mapk15	167	71	-1,18	7,79E-03
ENSDARG00000055288	prelid3a	49	20	-1,19	4,70E-02
ENSDARG00000077101	zbtb10	105	46	-1,19	8,18E-04
ENSDARG00000062096	pm20d1.2	513	224	-1,19	4,35E-07
ENSDARG00000098578	pdgfab	286	121	-1,19	3,14E-02
ENSDARG00000099483	vdra	66	28	-1,19	1,72E-02
ENSDARG00000013847	egfra	315	136	-1,19	2,40E-04
ENSDARG00000056647	klhl3	594	259	-1,19	1,13E-07
ENSDARG00000078011	NAV1 (1 of many)	135	58	-1,20	4,56E-03
ENSDARG00000002523	hsdl2	1013	438	-1,20	1,78E-06
ENSDARG00000024443	gnpda2	287	123	-1,20	6,41E-04
ENSDARG00000031647	stat2	287	121	-1,20	1,32E-02
ENSDARG00000025974	magi3b	243	103	-1,20	3,90E-03
ENSDARG00000037421	egr1	4925	2075	-1,20	1,70E-02
ENSDARG00000117133	cst14b.1	172	73	-1,20	1,84E-03
ENSDARG00000021987	plecb	551	232	-1,20	1,12E-02
ENSDARG00000040930	deptor	2261	970	-1,20	4,04E-05
ENSDARG00000059610	gpr146	696	297	-1,20	2,29E-04
ENSDARG00000095453	si:rp71-17i16.6	117	48	-1,21	4,22E-02
ENSDARG00000032990	plxnb3	174	73	-1,21	3,33E-02
ENSDARG00000000796	nr4a1	4096	1725	-1,21	4,48E-03
ENSDARG00000070227	nhsa	69	28	-1,21	4,02E-02
ENSDARG00000069264	si:ch211-196h16.5	36	14	-1,21	3,19E-02
ENSDARG00000054302	ehd4	518	218	-1,22	7,01E-04
ENSDARG00000094210	zgc:109934	96	39	-1,23	3,87E-02
ENSDARG00000114963	BX901962.12	22	8	-1,23	2,25E-02
ENSDARG00000058358	krt8	2070	864	-1,23	1,06E-03
ENSDARG00000075149	nagpa	149	62	-1,23	6,50E-04
ENSDARG00000086735	si:ch1073-111c8.3	90	38	-1,23	4,47E-03
ENSDARG00000012040	rnf19a	516	214	-1,23	1,56E-03
ENSDARG00000076265	ano8a	164	68	-1,24	3,73E-03
ENSDARG00000086739	SLC17A3	270	110	-1,24	1,90E-02
ENSDARG00000092731	mhc1uka	1532	625	-1,24	1,25E-02
ENSDARG00000068421	ttc9b	99	42	-1,24	3,55E-03
ENSDARG00000039150	lgmn	2121	888	-1,24	7,46E-07
ENSDARG00000054454	epha4a	688	280	-1,25	5,86E-03
ENSDARG00000055698	pcnxl2	346	139	-1,26	2,42E-02
ENSDARG00000058231	nt5c2b	194	81	-1,26	1,04E-04
ENSDARG00000089213	adam15	24	10	-1,26	4,88E-02

ENSDARG00000076014	ERC1	273	112	-1,26	4,77E-04
ENSDARG00000037905	cacna1aa	317	125	-1,26	3,80E-02
ENSDARG00000056477	ccdc125	241	97	-1,26	3,68E-03
ENSDARG00000117163	znf1114	19	7	-1,27	2,24E-02
ENSDARG00000099035	pcdh1g33	25	10	-1,27	3,97E-02
ENSDARG00000092407	zmp:0000001316	19	7	-1,27	4,99E-02
ENSDARG00000039931	slc25a33	671	273	-1,27	4,32E-04
ENSDARG00000059048	mpzl1l	152	61	-1,27	4,78E-03
ENSDARG00000008548	arhgap12a	1022	412	-1,27	4,92E-03
ENSDARG00000052617	raver1	381	154	-1,28	4,20E-04
ENSDARG00000009961	rundc3b	190	74	-1,28	3,54E-02
ENSDARG00000053130	pcp4a	540	213	-1,28	3,43E-02
ENSDARG00000011770	dhrs12	253	100	-1,28	1,30E-02
ENSDARG00000103370	lrrc8da	84	32	-1,28	3,93E-02
ENSDARG00000015732	cax2	61	24	-1,28	1,69E-02
ENSDARG00000004372	fmnl3	138	54	-1,28	9,79E-03
ENSDARG00000088342	tmem145	543	220	-1,28	2,65E-04
ENSDARG00000079584	zgc:194930	475	194	-1,29	3,71E-08
ENSDARG00000087088	si:ch211-274p24.4	27	10	-1,29	1,94E-02
ENSDARG00000063075	soga1	485	194	-1,29	1,60E-03
ENSDARG00000038398	FQ311927.1	71	28	-1,29	4,61E-02
ENSDARG00000053583	cers6	1194	480	-1,29	3,47E-05
ENSDARG00000052322	tlr5b	225	89	-1,29	8,59E-03
ENSDARG00000009040	si:ch73-141c7.1	90	35	-1,31	2,87E-02
ENSDARG00000009689	daam1b	197	77	-1,31	2,49E-04
ENSDARG00000059690	slc29a4	341	131	-1,32	7,16E-03
ENSDARG00000053636	cracr2b	161	62	-1,32	1,25E-02
ENSDARG00000077726	nocta	1518	597	-1,32	1,69E-05
ENSDARG00000104172	diabloa	71	28	-1,32	1,32E-02
ENSDARG00000031683	fosab	49096	19204	-1,33	1,65E-04
ENSDARG00000033978	nxn	77	29	-1,33	3,34E-02
ENSDARG00000029795	fam213b	235	88	-1,33	2,76E-02
ENSDARG00000029955	glb1l	243	95	-1,33	2,19E-04
ENSDARG00000055751	fosb	765	284	-1,34	2,90E-02
ENSDARG00000063032	dok6	56	21	-1,34	9,26E-03
ENSDARG00000034181	esr2b	197	73	-1,35	2,01E-02
ENSDARG00000040492	mthfd1b	121	44	-1,35	4,26E-02
ENSDARG00000093799	setbp1	203	79	-1,35	1,51E-07
ENSDARG00000053215	me1	257	97	-1,36	1,44E-04
ENSDARG00000051853	galns	134	51	-1,37	3,52E-04
ENSDARG00000098694	FQ311908.1	43	15	-1,37	2,16E-02
ENSDARG00000070498	phyhiplb	681	249	-1,37	2,09E-02
ENSDARG00000094268	si:ch73-337l15.2	40	15	-1,37	2,81E-02
ENSDARG00000007172	plxna3	205	77	-1,37	2,97E-03
ENSDARG00000037091	asah1a	120	44	-1,37	1,43E-02
ENSDARG00000100913	arhgef12b	246	92	-1,38	4,58E-04
ENSDARG00000028943	mtss1la	266	99	-1,38	4,01E-03
ENSDARG00000039373	epha3	89	33	-1,38	1,56E-03
ENSDARG00000094984	si:ch211-197g15.8	180	66	-1,38	1,77E-03
ENSDARG00000076339	cln5	88	33	-1,38	3,04E-04
ENSDARG00000058102	sardh	164	61	-1,38	2,18E-03
ENSDARG00000057767	nudt4a	166	61	-1,39	9,55E-04
ENSDARG00000018032	scn8ab	100	38	-1,39	5,70E-04
ENSDARG00000059826	crtac1a	822	301	-1,39	8,43E-03
ENSDARG00000103980	ets2	326	123	-1,39	1,45E-04
ENSDARG00000060723	stim1a	416	156	-1,39	1,11E-06
ENSDARG00000009881	ier5	1391	498	-1,39	2,59E-02
ENSDARG00000090552	si:dkey-7j14.6	2782	1025	-1,40	1,01E-03
ENSDARG00000102167	ppox	152	56	-1,40	5,76E-03
ENSDARG00000086222	nat16	157	57	-1,40	3,66E-03
ENSDARG00000038703	hkdc1	1565	583	-1,40	1,02E-06
ENSDARG00000021113	ptmaa	1367	503	-1,41	4,17E-04
ENSDARG00000040466	vil1	633	230	-1,41	1,21E-02
ENSDARG00000017986	vps13d	875	327	-1,41	1,49E-06
ENSDARG00000040717	macc1	476	176	-1,41	1,38E-05

ENSDARG00000092077	si:dkey-147f3.4	33	11	-1,41	3,95E-02
ENSDARG00000046030	zgc:110339	1670	619	-1,41	8,99E-08
ENSDARG00000036848	slc43a2a	1976	674	-1,42	3,98E-02
ENSDARG00000020298	btg2	13581	4947	-1,42	3,73E-04
ENSDARG00000057262	si:dkey-91i10.3	54	19	-1,42	3,29E-03
ENSDARG00000103394	kif5bb	1153	426	-1,42	9,30E-09
ENSDARG00000075048	lonrf1	470	170	-1,42	2,13E-03
ENSDARG00000079892	si:dkey-183c2.4	50	18	-1,43	2,82E-03
ENSDARG00000078645	si:ch73-62l21.1	22	8	-1,43	1,87E-02
ENSDARG00000076092	slc31a2	287	103	-1,43	4,77E-04
ENSDARG00000079133	si:dkey-10c21.1	151	54	-1,43	2,79E-06
ENSDARG00000007807	cyth3a	70	25	-1,43	1,95E-02
ENSDARG00000051748	ccnd2a	760	277	-1,43	1,20E-06
ENSDARG00000086881	ier2b	2026	729	-1,44	2,91E-04
ENSDARG00000070165	tnfrsf1b	344	124	-1,44	5,80E-05
ENSDARG00000039218	gpr101	104	36	-1,44	2,34E-02
ENSDARG00000015749	hps3	169	60	-1,44	2,07E-03
ENSDARG00000079572	plcd3b	36	12	-1,44	3,24E-02
ENSDARG00000061551	rap1gap2a	636	228	-1,44	3,57E-04
ENSDARG00000001463	tdh2	157	55	-1,45	1,38E-03
ENSDARG00000037069	zdhhc14	206	74	-1,45	1,42E-04
ENSDARG00000074311	mast2	702	254	-1,45	1,40E-05
ENSDARG00000070844	gamt	236	82	-1,46	3,49E-03
ENSDARG00000010789	homer3b	395	140	-1,46	1,30E-03
ENSDARG00000043249	irf1b	342	117	-1,46	3,15E-02
ENSDARG00000098497	hrasa	96	33	-1,46	4,61E-03
ENSDARG00000090036	RHOBTB3	160	56	-1,46	5,15E-03
ENSDARG00000102056	si:dkeyp-94b4.1	571	204	-1,46	1,02E-04
ENSDARG00000043531	jun	3024	1038	-1,46	8,56E-03
ENSDARG00000011490	scube3	276	95	-1,47	9,72E-03
ENSDARG00000077473	mych	124	42	-1,47	3,01E-02
ENSDARG00000097019	dhrs13b	92	32	-1,47	1,80E-02
ENSDARG00000008388	mmp14b	56	18	-1,47	4,52E-02
ENSDARG00000038359	enosf1	267	92	-1,47	2,39E-03
ENSDARG00000009939	micu2	96	33	-1,48	1,42E-03
ENSDARG00000006672	fam49a	2128	750	-1,48	2,48E-05
ENSDARG00000078882	slc22a31	61	21	-1,48	3,10E-03
ENSDARG00000103937	ndrg4	297	99	-1,48	1,73E-02
ENSDARG00000102572	si:ch211-260e23.9	1087	376	-1,48	1,56E-03
ENSDARG00000009499	syne1a	783	274	-1,49	8,71E-05
ENSDARG00000002411	pde4cb	414	141	-1,49	6,55E-03
ENSDARG00000055075	svila	517	173	-1,49	1,78E-02
ENSDARG00000055120	ctsba	573	199	-1,49	4,72E-05
ENSDARG00000062799	baiap2a	259	89	-1,50	1,14E-04
ENSDARG00000015907	dcbld1	215	75	-1,50	5,51E-05
ENSDARG00000062756	pllp	693	238	-1,50	1,22E-03
ENSDARG00000058820	bin1b	106	36	-1,50	1,50E-03
ENSDARG00000090444	ponzr1	1079	365	-1,50	3,37E-03
ENSDARG00000055831	si:dkey-182g1.6	92	30	-1,51	1,47E-02
ENSDARG00000090844	CABZ01054965.1	235	81	-1,51	7,16E-04
ENSDARG00000062020	gse1	389	136	-1,51	7,29E-07
ENSDARG00000058108	eps8l2	600	204	-1,51	5,36E-04
ENSDARG00000095019	lmo2	73	23	-1,51	1,78E-02
ENSDARG00000079291	rapgef3	375	127	-1,51	1,70E-03
ENSDARG00000037496	gria4a	256	87	-1,51	4,53E-04
ENSDARG00000071685	slco5a1a	755	257	-1,52	8,37E-05
ENSDARG00000000730	slc6a22.1	190	62	-1,52	1,63E-02
ENSDARG00000057296	cbln1	36	11	-1,52	4,94E-02
ENSDARG00000028173	slc4a2a	164	53	-1,53	3,07E-02
ENSDARG00000023287	hsd17b3	40	13	-1,54	1,51E-02
ENSDARG00000093403	si:ch73-376l24.4	55	18	-1,54	4,71E-03
ENSDARG00000058603	jph1a	168	56	-1,54	1,09E-03
ENSDARG00000030630	mfsd2aa	23	7	-1,54	3,14E-02
ENSDARG00000040177	rgs16	2476	829	-1,54	2,11E-04
ENSDARG00000028017	tp53inp1	801	267	-1,54	3,04E-04

ENSDARG00000054304	homeza	243	81	-1,55	4,47E-05
ENSDARG00000074233	pde4bb	260	87	-1,55	1,81E-04
ENSDARG00000079788	fam171a1	211	70	-1,55	4,10E-04
ENSDARG00000039500	LRFN3	310	102	-1,56	2,50E-03
ENSDARG00000004177	fam169ab	58	18	-1,56	1,16E-02
ENSDARG00000019498	cry5	44	14	-1,57	1,21E-02
ENSDARG00000102783	slc25a35	54	18	-1,57	9,74E-04
ENSDARG00000037140	pfkfb1	24	7	-1,57	3,96E-02
ENSDARG00000109715	sncgb	587	187	-1,57	1,00E-02
ENSDARG00000016866	fam102ab	260	82	-1,57	3,80E-03
ENSDARG00000028164	cst14a.1	25	8	-1,57	7,89E-03
ENSDARG00000035610	zgc:113208	235	75	-1,58	4,24E-04
ENSDARG00000002587	dpysl3	408	135	-1,58	1,89E-07
ENSDARG00000099724	relt	100	32	-1,58	8,62E-04
ENSDARG00000032317	tox	1853	606	-1,58	5,01E-06
ENSDARG00000014675	efna3a	82	26	-1,58	4,20E-03
ENSDARG00000016904	bckdk	3174	1037	-1,58	9,94E-06
ENSDARG00000054036	rnf34b	36	10	-1,58	3,44E-02
ENSDARG00000008568	slc25a24	315	103	-1,59	2,24E-05
ENSDARG00000055129	pim3	473	150	-1,59	2,68E-03
ENSDARG00000044345	cyfip1	54	16	-1,59	3,34E-02
ENSDARG00000074469	amigo3	945	303	-1,60	2,44E-04
ENSDARG00000103900	sept9b	1119	367	-1,60	5,05E-10
ENSDARG00000076027	kif5c	135	42	-1,62	5,16E-03
ENSDARG00000099106	afmid	91	29	-1,62	1,05E-03
ENSDARG00000001712	si:ch211-247n2.1	534	166	-1,62	1,28E-03
ENSDARG00000093612	si:dkey-92i17.2	24	7	-1,62	9,94E-03
ENSDARG00000099918	arl4aa	87	26	-1,63	5,67E-03
ENSDARG00000021735	cacna1ba	566	179	-1,63	8,60E-05
ENSDARG00000035879	fuca1.2	218	68	-1,63	3,65E-05
ENSDARG00000071029	si:dkey-147f3.4	59	17	-1,64	5,73E-03
ENSDARG00000004307	lypd6	275	84	-1,65	8,21E-04
ENSDARG00000060534	sgpp1	105	33	-1,65	5,76E-04
ENSDARG00000092726	si:dkey-184p9.7	90	26	-1,66	1,64E-02
ENSDARG00000101703	zgc:158260	141	41	-1,67	2,56E-02
ENSDARG00000051880	kcnj11	2145	663	-1,67	3,59E-07
ENSDARG00000090690	nell2a	415	122	-1,67	7,98E-03
ENSDARG00000077301	mlf1	56	16	-1,68	9,17E-03
ENSDARG00000004363	lhfpl6	183	54	-1,68	1,77E-04
ENSDARG00000103535	SORCS3	218	65	-1,68	4,70E-03
ENSDARG00000074378	junba	3355	1015	-1,68	3,08E-05
ENSDARG00000057410	rxfp3	1096	329	-1,68	6,36E-04
ENSDARG00000026070	cd82b	210	65	-1,68	6,92E-06
ENSDARG00000092998	si:ch211-202f5.3	467	143	-1,69	5,87E-09
ENSDARG00000079917	rassf7a	52	14	-1,69	6,96E-03
ENSDARG00000070174	si:ch211-18i17.2	570	166	-1,69	1,68E-03
ENSDARG00000007788	atp2b1b	208	58	-1,71	6,31E-03
ENSDARG00000086645	hs3st3b1b	2529	758	-1,71	1,61E-07
ENSDARG00000025428	socs3a	1420	388	-1,72	1,17E-02
ENSDARG00000102332	spint1a	366	107	-1,72	9,66E-06
ENSDARG00000037921	gng13b	1309	388	-1,72	9,42E-07
ENSDARG00000089156	egr3	35	10	-1,73	1,27E-02
ENSDARG00000058226	ak3	489	143	-1,73	3,32E-05
ENSDARG00000019062	arpc5b	968	285	-1,73	1,10E-06
ENSDARG00000016412	agt	173	48	-1,74	2,90E-03
ENSDARG00000096902	si:ch211-180n24.3	22	6	-1,74	1,58E-02
ENSDARG00000115646	CR846082.1	46	13	-1,74	1,56E-03
ENSDARG00000010572	slc25a25a	423	122	-1,74	3,36E-05
ENSDARG00000056267	dixdc1b	274	79	-1,75	1,20E-08
ENSDARG00000059972	raly	542	148	-1,75	1,62E-02
ENSDARG00000102312	scn12ab	101	29	-1,75	6,88E-04
ENSDARG00000031336	hsd20b2	111	29	-1,76	1,07E-02
ENSDARG00000055159	cyp27a1.4	77	21	-1,77	3,81E-03
ENSDARG00000001909	vill	176	46	-1,77	2,47E-02
ENSDARG00000077948	usp54a	534	154	-1,78	8,15E-09

ENSDARG00000110186	CDCP1	102	28	-1,78	9,45E-04
ENSDARG00000029072	klf6a	119	32	-1,78	1,54E-03
ENSDARG00000076144	si:ch211-74m13.3	36	9	-1,78	2,57E-02
ENSDARG00000088251	si:ch211-24o10.6	55	12	-1,79	4,02E-02
ENSDARG00000069708	cdc34a	133	37	-1,79	1,96E-04
ENSDARG00000103114	ndufa4l2a	6200	1677	-1,79	5,26E-03
ENSDARG00000062220	plekha7b	126	36	-1,79	4,59E-06
ENSDARG00000039133	lamb4	38	10	-1,80	4,68E-02
ENSDARG00000099841	PRKCA	189	52	-1,80	2,60E-04
ENSDARG00000023214	mob2a	38	10	-1,80	1,13E-02
ENSDARG00000069135	ppp1r15a	1926	529	-1,80	1,37E-04
ENSDARG00000037154	hivep3b	91	25	-1,80	7,91E-04
ENSDARG00000087196	fibinb	189	51	-1,80	1,76E-03
ENSDARG00000024195	znf395b	493	138	-1,80	1,97E-07
ENSDARG00000001656	psmb13a	124	34	-1,80	1,87E-04
ENSDARG00000015278	plxnc1	55	14	-1,81	2,65E-02
ENSDARG00000037852	zgc:101663	68	18	-1,81	2,35E-04
ENSDARG00000038655	ajap1	193	51	-1,81	1,91E-03
ENSDARG00000043006	gnav1	29	8	-1,81	2,67E-02
ENSDARG00000036787	tap2a	20	5	-1,81	8,35E-03
ENSDARG00000058939	b4galnt3b	30	7	-1,81	1,84E-03
ENSDARG00000057665	sprn2	636	174	-1,81	4,43E-05
ENSDARG00000074530	KCNK12	39	10	-1,82	4,02E-02
ENSDARG00000013076	bmi1b	211	56	-1,82	1,19E-03
ENSDARG00000088258	CABZ01038494.1	172	46	-1,82	1,53E-05
ENSDARG00000008790	actr3b	45	11	-1,82	3,37E-03
ENSDARG00000032708	arl4cb	79	20	-1,82	1,85E-02
ENSDARG00000020594	txlnba	24	6	-1,82	3,49E-02
ENSDARG00000051926	plag1	43	11	-1,83	3,52E-04
ENSDARG00000013946	ivns1abpb	137	36	-1,84	3,06E-05
ENSDARG00000068745	map4l	755	210	-1,84	1,37E-09
ENSDARG00000078155	INAVA	186	50	-1,84	6,07E-04
ENSDARG00000077341	ppp1r14c	38	9	-1,84	3,86E-02
ENSDARG00000013628	cd164	8651	2337	-1,84	6,45E-06
ENSDARG00000033614	rasgef1ba	276	63	-1,84	1,85E-02
ENSDARG00000062575	si:ch211-210g13.5	20	5	-1,84	2,04E-02
ENSDARG00000058260	cip2a	51	12	-1,84	3,37E-02
ENSDARG00000056619	arhgap42a	63	15	-1,85	2,15E-02
ENSDARG00000026611	socs3b	372	93	-1,86	2,35E-03
ENSDARG00000070025	dchs1a	295	70	-1,86	1,47E-02
ENSDARG00000039265	arhgap4a	44	11	-1,87	1,08E-03
ENSDARG00000012340	ptpn11b	169	44	-1,87	2,54E-04
ENSDARG00000112970	BX571719.1	36	8	-1,88	4,76E-02
ENSDARG00000078186	tex2	1032	277	-1,89	6,49E-11
ENSDARG00000013787	gucy1a1	504	123	-1,89	2,31E-02
ENSDARG00000037917	itga3a	79	17	-1,89	4,19E-02
ENSDARG00000090946	si:zfos-364h11.1	27	6	-1,89	4,29E-02
ENSDARG00000098408	stbd1	60	15	-1,90	3,42E-03
ENSDARG00000007179	sash1a	221	57	-1,91	1,52E-05
ENSDARG00000060112	sybu	160	36	-1,92	1,17E-02
ENSDARG00000044415	tlr5a	197	47	-1,92	2,07E-03
ENSDARG00000004724	tcea3	67	16	-1,93	1,78E-03
ENSDARG00000075163	cxcl20	296	73	-1,93	9,94E-05
ENSDARG00000054842	hsd17b14	128	32	-1,93	1,66E-05
ENSDARG00000011473	calcrla	558	143	-1,93	2,87E-09
ENSDARG00000003655	tmem59l	330	79	-1,93	1,05E-03
ENSDARG00000029766	nr1i2	262	65	-1,94	5,25E-05
ENSDARG00000076404	mctp1a	224	55	-1,94	3,26E-03
ENSDARG00000076135	mmrn2a	24	5	-1,94	4,99E-02
ENSDARG00000095152	si:ch211-255i3.4	1229	314	-1,94	3,57E-09
ENSDARG00000037191	ttr	58	14	-1,95	3,56E-03
ENSDARG00000089862	si:dkey-16j16.4	154	39	-1,96	3,86E-08
ENSDARG00000075924	zgc:171558	23	5	-1,96	3,21E-02
ENSDARG00000078523	ddr1	364	84	-1,97	1,63E-03
ENSDARG00000074425	si:busm1-57f23.1	433	96	-1,97	6,11E-03

ENSDARG00000032705	foxg1b	30	5	-1,97	4,14E-02
ENSDARG00000071772	si:ch211-253p2.2	393	97	-1,97	1,20E-05
ENSDARG00000019260	dhrs9	26	5	-1,97	4,97E-02
ENSDARG00000057527	mef2ab	96	23	-1,98	3,44E-04
ENSDARG00000044254	anxa3b	52	11	-1,98	1,76E-02
ENSDARG00000008127	pcdh15b	1133	276	-1,99	7,38E-06
ENSDARG00000007836	ctsla	2605	632	-1,99	2,34E-06
ENSDARG00000105562	si:dkey-23i12.7	63	14	-1,99	4,73E-03
ENSDARG00000071215	palm3	352	86	-1,99	1,74E-08
ENSDARG00000034473	ttyh3a	442	107	-2,00	9,91E-07
ENSDARG00000105450	si:ch211-63p21.1	50	10	-2,00	3,01E-02
ENSDARG00000061120	slc43a2b	4336	1068	-2,00	1,48E-09
ENSDARG00000045589	kcnj8	204	43	-2,01	2,79E-02
ENSDARG00000062462	shisa7a	21	4	-2,02	3,03E-02
ENSDARG00000103903	LO017650.1	52	11	-2,02	7,25E-03
ENSDARG00000052336	ociad2	54	12	-2,03	1,94E-03
ENSDARG00000039974	rai14	37	7	-2,05	9,77E-03
ENSDARG00000038428	sulf1	490	104	-2,05	4,21E-03
ENSDARG00000013613	anxa13l	566	123	-2,05	1,48E-03
ENSDARG00000045753	tmtc2a	38	7	-2,06	2,12E-02
ENSDARG00000074827	si:ch73-184c24.1	182	39	-2,06	1,18E-03
ENSDARG00000015985	abcc9	217	48	-2,06	1,76E-03
ENSDARG00000063416	ptprk	189	43	-2,06	7,96E-05
ENSDARG00000031348	mc5ra	1017	227	-2,06	7,53E-05
ENSDARG00000026052	ccdc3a	55	10	-2,06	8,35E-03
ENSDARG00000101495	ugt5b3	22	4	-2,08	1,35E-02
ENSDARG00000112100	neto2b	17	4	-2,09	1,21E-02
ENSDARG00000102724	zgc:153039	47	9	-2,09	1,04E-02
ENSDARG00000021352	gria1a	341	69	-2,10	1,69E-02
ENSDARG00000098112	btc	1723	391	-2,10	2,99E-08
ENSDARG00000069427	nkain2	29	5	-2,10	1,79E-02
ENSDARG00000095415	si:ch211-202f5.2	69	14	-2,10	1,81E-03
ENSDARG00000052242	aff2	33	6	-2,11	2,93E-02
ENSDARG00000042188	ttc38	21	4	-2,12	1,27E-02
ENSDARG00000037320	fam131c	593	131	-2,13	1,86E-06
ENSDARG00000057107	si:ch73-383g2.1	47	8	-2,13	3,54E-02
ENSDARG00000097371	mcf2b	33	6	-2,13	2,85E-02
ENSDARG00000030269	grtp1b	47	9	-2,14	1,07E-02
ENSDARG00000015355	fosl1a	2740	585	-2,14	1,50E-05
ENSDARG00000043180	gpd1b	3597	799	-2,15	2,09E-13
ENSDARG00000053190	galnt16	130	26	-2,15	4,23E-04
ENSDARG00000009142	ppp1r13bb	740	163	-2,15	5,64E-11
ENSDARG00000014910	panx1b	229	48	-2,16	9,95E-05
ENSDARG00000068128	ppp1r15b	187	40	-2,16	4,47E-05
ENSDARG00000077799	egr4	12308	2579	-2,16	2,18E-05
ENSDARG00000086374	isg15	347	65	-2,16	5,68E-03
ENSDARG00000100104	zgc:101785	72	13	-2,17	1,51E-02
ENSDARG00000106258	SGCZ	26	5	-2,17	7,97E-03
ENSDARG00000106873	LO018188.1	531	111	-2,17	7,09E-06
ENSDARG00000059523	rims4	129	26	-2,18	5,10E-04
ENSDARG00000036840	krt15	585	119	-2,18	2,76E-04
ENSDARG00000070903	met	177	37	-2,19	1,11E-04
ENSDARG00000056704	zgc:91818	208	40	-2,19	5,57E-03
ENSDARG00000089162	afap1l1a	279	58	-2,19	1,44E-05
ENSDARG00000060532	adam22	242	47	-2,19	6,48E-04
ENSDARG00000007751	lmnl3	184	37	-2,20	3,19E-04
ENSDARG00000024540	tspan36	1781	366	-2,20	8,04E-06
ENSDARG00000108535	CABZ01072077.1	74	13	-2,21	1,32E-02
ENSDARG00000001807	tnfrsf21	646	137	-2,21	1,92E-09
ENSDARG00000028213	ttn.2	40	4	-2,21	1,56E-02
ENSDARG00000062501	hhipl1	55	10	-2,21	3,15E-02
ENSDARG00000078468	fap	51	9	-2,22	7,67E-03
ENSDARG00000002196	bach1b	206	42	-2,22	4,25E-07
ENSDARG00000097503	si:ch73-288o11.5	241	46	-2,22	4,03E-03
ENSDARG00000051915	pde5ab	69	13	-2,23	9,93E-04

ENSDARG00000060253	si:ch211-216b21.2	41	7	-2,24	3,61E-03
ENSDARG00000027355	slc25a4	39	7	-2,24	3,42E-02
ENSDARG00000007370	dysf	46	8	-2,24	5,10E-03
ENSDARG00000010250	nqo1	52	9	-2,26	1,27E-02
ENSDARG00000070423	zgc:153157	99	18	-2,26	5,88E-04
ENSDARG00000045485	rassf8b	66	13	-2,27	1,01E-05
ENSDARG00000101348	plppr5b	576	114	-2,27	5,18E-07
ENSDARG00000093458	si:dkey-76d14.2	21	4	-2,30	7,55E-03
ENSDARG00000099195	ier2a	14915	2870	-2,31	3,23E-07
ENSDARG00000093549	selenop	503	78	-2,31	2,08E-02
ENSDARG00000018404	krt18	2850	535	-2,31	8,79E-06
ENSDARG00000075017	myzap	344	64	-2,33	6,05E-06
ENSDARG00000012555	ak5	437	66	-2,33	9,78E-03
ENSDARG00000093193	chia.6	172	29	-2,33	3,25E-03
ENSDARG00000038235	pkdccb	45	7	-2,33	3,46E-03
ENSDARG00000013317	pygmb	220	40	-2,34	2,53E-05
ENSDARG00000055302	grid2	109	15	-2,34	1,88E-02
ENSDARG00000031702	prkg1b	38	4	-2,35	8,41E-03
ENSDARG00000075706	si:ch211-106h4.9	361	63	-2,36	7,67E-04
ENSDARG00000098588	gchfr	1892	352	-2,36	2,71E-07
ENSDARG00000089790	efna5a	56	8	-2,36	6,34E-03
ENSDARG00000116487	CABZ01039304.1	160	28	-2,37	2,38E-04
ENSDARG00000074698	sdsl	81	13	-2,37	2,09E-03
ENSDARG00000004218	rnd1b	323	53	-2,38	3,85E-03
ENSDARG00000035350	ins	2313357	398302	-2,38	5,06E-03
ENSDARG00000057630	galm	45	7	-2,38	1,45E-03
ENSDARG00000059923	slc25a47a	22	4	-2,39	3,01E-03
ENSDARG00000031600	rd3	64	10	-2,40	1,61E-03
ENSDARG00000017780	rorcb	75	11	-2,42	7,61E-03
ENSDARG00000087084	hcar1-4	34	4	-2,42	5,27E-03
ENSDARG00000057698	ctsd	4067	731	-2,43	2,99E-10
ENSDARG00000057062	si:dkey-157g16.6	18	3	-2,43	3,75E-03
ENSDARG00000077641	thbs3a	81	13	-2,46	1,51E-03
ENSDARG00000054323	pparab	79	14	-2,46	1,54E-06
ENSDARG00000014462	rab3c	1477	262	-2,47	5,06E-14
ENSDARG00000086851	si:dkey-264d12.5	87	11	-2,47	5,49E-03
ENSDARG00000102096	adra2b	114	18	-2,47	6,81E-03
ENSDARG00000053381	plpp1a	145	24	-2,48	2,81E-06
ENSDARG00000061421	FQ311928.1	92	14	-2,48	7,24E-04
ENSDARG00000021948	tnc	333	50	-2,48	1,24E-04
ENSDARG00000020693	sesn1	751	118	-2,50	4,83E-05
ENSDARG00000077465	sorcs2	55	6	-2,50	3,58E-03
ENSDARG00000013838	sulf2b	56	8	-2,50	1,12E-03
ENSDARG00000042840	eml1	19	1	-2,50	1,10E-02
ENSDARG00000095674	tmem123	23	3	-2,50	9,94E-03
ENSDARG00000101457	cdc42ep4a	371	58	-2,51	5,25E-05
ENSDARG00000063621	aadac	46	7	-2,52	6,94E-03
ENSDARG00000041607	eif4ebp3l	69	9	-2,52	2,11E-03
ENSDARG00000104259	CABZ01072885.1	57	8	-2,53	1,34E-04
ENSDARG00000089217	ubap1la	22	3	-2,54	4,91E-03
ENSDARG00000057017	slc6a4b	39	5	-2,55	3,32E-03
ENSDARG00000093019	si:dkey-83k24.5	150	18	-2,56	7,77E-03
ENSDARG00000019728	bmpr1aa	1058	172	-2,59	1,46E-13
ENSDARG00000071250	tmem79a	21	3	-2,61	2,07E-03
ENSDARG00000073684	quo	72	9	-2,61	1,46E-02
ENSDARG00000091111	si:ch211-15b10.6	54	6	-2,62	1,87E-03
ENSDARG00000034368	hexb	969	150	-2,64	4,32E-12
ENSDARG00000086300	si:ch211-236d3.4	54	7	-2,66	1,65E-03
ENSDARG00000099728	zgc:56622	55	8	-2,66	5,11E-05
ENSDARG00000059001	pim2	402	56	-2,67	1,44E-05
ENSDARG00000105088	tenm4	37	5	-2,69	8,16E-04
ENSDARG00000005989	rgl1	138	18	-2,69	3,63E-05
ENSDARG00000079895	ehbp1l1b	33	4	-2,70	1,54E-03
ENSDARG00000100475	fgf10b	69	7	-2,74	2,81E-02
ENSDARG00000044098	egr2a	73	8	-2,74	4,56E-04

ENSDARG00000098312	scarb2a	493	56	-2,74	5,01E-03
ENSDARG00000093931	rflnb	98	13	-2,74	2,67E-05
ENSDARG00000068849	fam83ha	293	37	-2,76	2,03E-05
ENSDARG00000094935	si:dkey-58f10.11	34	4	-2,78	1,21E-03
ENSDARG00000026499	ppm1e	113	15	-2,78	1,11E-05
ENSDARG00000078433	spc25	140	16	-2,81	8,85E-04
ENSDARG00000027609	hpn	737	93	-2,82	4,36E-06
ENSDARG00000020924	myo1ca	36	4	-2,82	1,43E-02
ENSDARG00000042826	egr2b	54	4	-2,83	1,83E-03
ENSDARG00000098777	CU693379.1	34	4	-2,84	1,01E-03
ENSDARG00000077069	srgn	47	4	-2,86	1,25E-02
ENSDARG00000094526	ksr2	57	6	-2,86	3,51E-03
ENSDARG00000075249	fam171a2a	68	7	-2,91	7,73E-05
ENSDARG00000059601	map1aa	182	19	-2,92	4,35E-05
ENSDARG00000029036	rab32a	89	9	-2,93	3,95E-04
ENSDARG00000043341	npy4r	305	33	-2,96	9,30E-05
ENSDARG00000040841	adra2a	322	31	-2,97	5,06E-04
ENSDARG00000076246	prune	353	42	-2,98	6,70E-09
ENSDARG00000077217	zgc:162184	60	6	-2,98	4,18E-04
ENSDARG00000007804	gas6	29	2	-2,98	1,61E-02
ENSDARG00000052997	sema4e	42	4	-2,99	1,26E-04
ENSDARG00000088143	sema4gb	21	2	-3,00	1,87E-03
ENSDARG00000076828	zgc:194101	144	1	-3,02	1,00E-02
ENSDARG00000021869	rcan2	1258	144	-3,02	5,42E-09
ENSDARG00000069199	zgc:172120	119	11	-3,02	4,03E-04
ENSDARG00000094557	nupr1	1899	182	-3,03	3,00E-04
ENSDARG00000056656	tspan18a	884	74	-3,06	1,48E-03
ENSDARG00000062147	otc	563	58	-3,07	2,38E-05
ENSDARG00000002745	tdh	848	87	-3,07	4,25E-06
ENSDARG00000102138	foxa1	120	11	-3,08	1,96E-04
ENSDARG00000014927	uts1	179	8	-3,08	1,12E-03
ENSDARG00000021137	adgrv1	120	8	-3,08	5,08E-04
ENSDARG00000012449	si:ch211-169p10.1	31	3	-3,09	9,85E-05
ENSDARG00000010318	srpx	500	55	-3,09	9,30E-09
ENSDARG00000089529	desi1b	28	2	-3,11	1,29E-05
ENSDARG00000052121	rag2	109	10	-3,13	1,50E-04
ENSDARG00000017367	rhbdf1b	77	7	-3,16	9,80E-04
ENSDARG00000070734	dyrk4	48	4	-3,17	2,43E-04
ENSDARG00000054683	prdm8b	34	3	-3,20	8,30E-05
ENSDARG00000020541	ism1	40	3	-3,20	1,87E-03
ENSDARG00000079105	mhc2dab	140	6	-3,20	1,78E-02
ENSDARG00000062672	kcnj19b	995	92	-3,22	3,18E-07
ENSDARG00000071331	ryr3	173	14	-3,23	8,58E-06
ENSDARG00000077162	nwd2	211	21	-3,24	3,50E-10
ENSDARG00000040198	fstl5	50	3	-3,28	4,58E-04
ENSDARG00000020953	dnajb6b	238	22	-3,28	1,13E-09
ENSDARG00000005716	olfml2ba	96	7	-3,30	2,23E-04
ENSDARG00000042277	efnb3b	281	25	-3,34	2,98E-07
ENSDARG00000015536	sox6	155	13	-3,34	5,51E-07
ENSDARG00000105059	zgc:172282	94	7	-3,34	2,51E-05
ENSDARG00000076729	myo9aa	94	7	-3,34	5,89E-06
ENSDARG00000093176	zgc:55413	23	1	-3,39	1,07E-02
ENSDARG00000069560	kcnh3	26	1	-3,41	1,18E-02
ENSDARG00000025667	adgrb2	287	22	-3,42	1,36E-05
ENSDARG00000071596	neto1l	225	17	-3,45	1,46E-06
ENSDARG00000074671	nptx1l	115	6	-3,46	5,81E-03
ENSDARG00000073970	mctp2b	411	32	-3,46	5,08E-07
ENSDARG00000058734	prdx1	113	7	-3,47	6,99E-05
ENSDARG00000093748	si:ch211-217k17.11	32	0	-3,47	1,47E-02
ENSDARG00000089362	grn1	339	22	-3,50	5,43E-05
ENSDARG00000037097	slc7a2	3190	235	-3,51	1,19E-06
ENSDARG00000042861	ltk	79	5	-3,52	8,62E-05
ENSDARG00000017739	ak5l	1380	101	-3,54	1,46E-07
ENSDARG00000098113	CABZ01007222.1	111	6	-3,55	2,09E-04
ENSDARG00000102050	LO017850.1	99	6	-3,58	8,75E-05

ENSDARG00000056603	mcf2b	213	10	-3,59	3,52E-04
ENSDARG00000099411	zgc:158343	176	10	-3,59	6,60E-05
ENSDARG00000034522	rab6ba	24	1	-3,61	1,54E-03
ENSDARG00000040528	lgals3bpb	23	1	-3,63	2,13E-03
ENSDARG00000004782	fgfr3	227	16	-3,64	5,01E-08
ENSDARG00000097209	cox8b	453	26	-3,66	2,20E-06
ENSDARG00000007356	fgf20a	32	2	-3,68	2,44E-04
ENSDARG00000061752	ripor2	23	1	-3,68	2,06E-03
ENSDARG00000017388	gstt1b	60	3	-3,68	8,41E-06
ENSDARG00000041959	cxcr4b	37	0	-3,70	1,89E-02
ENSDARG00000034718	tfpia	173	6	-3,71	7,31E-05
ENSDARG00000025914	si:dkey-190g11.3	48	2	-3,72	8,80E-05
ENSDARG00000002564	syngr1a	75	4	-3,73	7,76E-06
ENSDARG00000104068	gstp1	4154	285	-3,73	1,13E-10
ENSDARG00000036074	cebpa	33	1	-3,75	2,16E-03
ENSDARG00000031598	calb1	250	13	-3,76	2,94E-04
ENSDARG00000035852	cart3	44	1	-3,76	8,89E-04
ENSDARG00000076858	TCIM (1 of many)	131	7	-3,77	2,19E-05
ENSDARG00000056230	ghsra	134	8	-3,77	7,12E-07
ENSDARG00000088911	cbln17	135	8	-3,79	2,04E-07
ENSDARG00000077826	efcc1	65	3	-3,80	5,87E-05
ENSDARG00000045066	entpd1	92	4	-3,83	3,73E-04
ENSDARG00000016718	mmp11b	393	24	-3,85	8,90E-10
ENSDARG00000101209	lamb1a	241	13	-3,85	1,40E-06
ENSDARG00000020771	tnr	51	3	-3,87	9,91E-05
ENSDARG00000074781	ptprt	166	4	-3,87	8,53E-03
ENSDARG00000063437	wnt9a	226	7	-3,88	4,05E-05
ENSDARG00000099129	phlda1	93	5	-3,90	7,39E-07
ENSDARG00000092600	si:dkey-256e7.5	37	1	-3,91	1,00E-03
ENSDARG00000043662	cnih2	44	2	-3,94	1,05E-05
ENSDARG00000106669	CABZ01068358.1	446	24	-3,95	1,69E-08
ENSDARG00000076799	rnd3a	26	1	-3,96	9,71E-04
ENSDARG00000061665	hcn2b	148	8	-3,97	3,66E-07
ENSDARG00000038814	myrip	247	12	-3,99	1,10E-06
ENSDARG00000099238	rasd4	2520	105	-4,01	8,30E-05
ENSDARG00000103638	CR936977.2	21	0	-4,02	6,10E-03
ENSDARG00000010946	cbsb	2089	107	-4,05	4,22E-09
ENSDARG00000099387	jpt1a	108	4	-4,05	4,41E-06
ENSDARG00000045486	pawr	122	7	-4,06	3,76E-10
ENSDARG00000114920	TTC28	138	5	-4,08	8,04E-06
ENSDARG00000002576	cabp5a	51	1	-4,10	4,09E-04
ENSDARG00000004111	esr1	1145	57	-4,16	2,85E-11
ENSDARG00000070110	opn5	40	1	-4,16	2,70E-05
ENSDARG00000070228	cdk6	43	2	-4,16	4,57E-06
ENSDARG00000090140	tmem196b	385	20	-4,17	1,14E-10
ENSDARG00000053301	insm1b	871	36	-4,18	6,88E-08
ENSDARG00000001733	gulp1a	24	0	-4,19	8,97E-05
ENSDARG00000077709	iqsec2b	446	20	-4,21	8,79E-09
ENSDARG00000075905	si:ch211-266g18.9	25	0	-4,26	2,09E-02
ENSDARG00000039240	cacng5b	48	1	-4,30	6,83E-04
ENSDARG00000006025	tp53i11b	24	0	-4,31	3,25E-03
ENSDARG00000056228	pld1a	154	6	-4,31	1,76E-09
ENSDARG00000007490	adrb1	37	0	-4,33	1,69E-05
ENSDARG00000060626	dgkaa	1135	41	-4,33	2,31E-06
ENSDARG00000057479	hrh2b	733	24	-4,34	2,20E-06
ENSDARG00000034504	lmo1	809	25	-4,38	6,09E-07
ENSDARG00000097055	si:ch211-284f22.3	44	1	-4,39	2,04E-05
ENSDARG00000011673	si:ch211-246m6.5	83	2	-4,40	1,52E-04
ENSDARG00000036816	pou2f2a	299	10	-4,41	3,49E-08
ENSDARG00000012204	CDK18	206	8	-4,44	8,08E-09
ENSDARG00000035942	hrh3	424	16	-4,49	3,51E-11
ENSDARG00000007461	srgap1a	138	4	-4,50	1,11E-08
ENSDARG00000063538	kalrnb	830	27	-4,51	4,22E-08
ENSDARG00000010727	ttyh2l	315	9	-4,51	4,81E-07
ENSDARG00000030905	cited2	23	0	-4,67	5,28E-05

ENSDARG00000103498	epd	375	2	-4,69	2,58E-02
ENSDARG00000041960	si:ch211-173a9.6	127	0	-4,72	1,17E-03
ENSDARG00000116524	zgc:103700	57	0	-4,75	6,66E-04
ENSDARG00000014670	colgalt2	119	3	-4,80	1,99E-06
ENSDARG00000002847	fndc1	24	0	-4,83	7,49E-03
ENSDARG00000076520	mafb	1539	38	-4,84	4,53E-08
ENSDARG00000032565	cacng2a	104	2	-4,86	1,52E-07
ENSDARG00000010083	rbfox3a	325	8	-4,88	9,13E-09
ENSDARG00000021584	cntn5	266	6	-4,91	5,08E-07
ENSDARG00000013926	slc16a9a	435	10	-4,95	1,30E-08
ENSDARG00000035468	slc25a25b	822	19	-4,97	3,97E-10
ENSDARG00000029372	rasa4	623	15	-5,03	3,76E-10
ENSDARG00000079876	CU855878.1	1013	22	-5,04	7,38E-10
ENSDARG00000062821	slc6a15	116	1	-5,06	8,03E-05
ENSDARG00000013082	uap1l1	583	12	-5,17	4,70E-11
ENSDARG00000112040	CR774179.5	376	4	-5,19	1,81E-07
ENSDARG00000088202	sh3d19	576	11	-5,20	7,07E-10
ENSDARG00000051939	pcxb	8566	152	-5,29	3,16E-09
ENSDARG00000074372	elfn1b	93	1	-5,37	1,22E-05
ENSDARG00000099791	pdk3b	57	1	-5,47	5,22E-08
ENSDARG00000104022	gcgra	155	3	-5,52	2,11E-06
ENSDARG00000035544	etnppl	1594	25	-5,53	3,45E-10
ENSDARG00000077866	nostrin	109	2	-5,56	4,33E-09
ENSDARG00000103750	si:ch211-246m6.5	530	7	-5,63	1,22E-09
ENSDARG00000032435	itga9	289	4	-5,67	3,04E-10
ENSDARG00000027584	ttpa	647	4	-5,81	2,95E-05
ENSDARG00000100336	zgc:158654	367	1	-5,85	6,04E-04
ENSDARG00000043010	camk2d1	502	5	-6,04	5,40E-10
ENSDARG00000060637	clstn2	602	4	-6,44	3,50E-10
ENSDARG00000021186	si:dkeyp-4h4.1	63	0	-6,54	1,38E-04
ENSDARG00000076401	cacng3b	361	2	-6,60	1,68E-09
ENSDARG00000062449	xpr1a	239	2	-6,68	1,60E-11
ENSDARG00000069459	si:dkey-6n21.13	247	1	-6,70	3,58E-09
ENSDARG00000035984	mnx1	1808	10	-6,92	3,83E-13
ENSDARG00000078768	abhd15a	4014	15	-6,94	3,66E-09
ENSDARG00000039576	fstl1b	1644	6	-6,99	3,50E-10
ENSDARG00000088708	ngfra	963	5	-7,18	3,90E-13
ENSDARG00000104735	nkx6.2	1025	0	-9,84	1,13E-10
