# Supplementary material for: A δ-cell subpopulation with a pro-β-cell identity contributes to efficient age-independent recovery in a zebrafish model of diabetes
Source: eLife. 2022 Jan 21;11:e67576. doi: 10.7554/eLife.67576 (PMC8820734; doi:10.7554/eLife.67576)
Supplement: Figure 3—source data 2. [file elife-67576-fig3-data2.pdf]

Figure 3-Source Data 2. β-cell markers from Tarifeño-Saldivia et al (2017)

β–cell markers enriched >4-fold versus the other main pancreatic cell types, α-, sst2 δ-, acinar and duct, based on RNAseq data from Tarifeño-Saldivia 2017 and mapped on more recent GRCz11 v92

ins
ppdpfb
ndufa4l2a
g6pcb
tagln3b
wif1
pcdh10a
kcnj2a
ca7
fstl1b
esr1
nptna
zgc:154058
ucn3l
ca12
rasd4
klb
prkdc
otc
dpp6b
tmtc1
tmeff2a
man2c1
prkcbb
srpx
nkx6.2
ngfra
fgfr1b
cntn4
tmem163a
rrad
CABZ01085139.1
ace
slc38a11
grapb
rnd1b
stmn4
nkx3.2
ppox
fstl1a
plxnb3
npm2a
cacng3b
cart3
gabrb4
iqsec2b
nlgn4b
CSMD3 (1 of many)
LO017650.1
snx8b
chrm5a
pnocb
si:dkey-215k6.1
hcn1
zgc:91818
arvcfa
grin2bb
bcl2b
zgc:158260
runx2a
si:ch211-242f23.3
orc6
