# Supplementary material for: A δ-cell subpopulation with a pro-β-cell identity contributes to efficient age-independent recovery in a zebrafish model of diabetes
Source: eLife. 2022 Jan 21;11:e67576. doi: 10.7554/eLife.67576 (PMC8820734; doi:10.7554/eLife.67576)
Supplement: Figure 3—source data 3. [file elife-67576-fig3-data3.pdf]

Figure 3-Source Data 3. Gene Ontology analysis and pathways overrepresented in bihormonal cells

GO from genes overexpressed in 20dpt bihormonal cells versus CTL Beta (DESeq FC>2x, Padj<0.05)
GO Padj (FDR) <0.25

Biological Process (non redundant)

geneSet	description	enrichmentRatio	pValue	FDR	userId
GO:0022610	biological adhesion	2,544078307	1,54E-05	0,00438131	adora2b;dlg1;cdh4;ek1;pcdh19;arrb1;nrxn3a;ret;epdl2;cdh10a;plxna2;emilin2a;nlgn3b;nrxn3b;pcdh1a;celsr1a;epdl1;nlgn2a;nlgn1;runx1;pcdh15a;vstm2l;pcdh10b;itga4;inppl1a
GO:0099504	synaptic vesicle cycle	4,358854167	4,15E-04	0,0589034	dnm1b;syt4;syt11b;snap25b;nlgn3b;si:ch211-81a5.8;nlgn2a;nlgn1
GO:0050808	synapse organization	4,10245098	6,34E-04	0,05998269	dlg1;bdnf;npas4a;nlgn3b;nlgn2a;nlgn1;pclob;gphnb
GO:0051336	regulation of hydrolase activity	1,879266467	0,00213353	0,15148057	llgl1;fgfr1a;iqsec1b;sh3bp4a;ek1;crim1;serpini1;atp1b2b;ppp1r14ba;ppp1r3b;bik;c3a.3;si:dkey-191g9.5;serping1;srgap3;plxna2;elmod1;ppp2r2cb;syngap1b;arhgap32b;dab2ipb;myo9b;si:dkey-117n7.5;abr
GO:0009887	animal organ morphogenesis	1,683677575	0,00339133	0,16502121	sox9a;ispd;slc35b2;mef2cb;fgfr1a;slc26a2;cdh4;ptch1;bcor;atp2a2a;crim1;dnmt1;pcdh19;atp1b2b;prickle1a;notch2;ostn;ngs;slc8a4a;ret;lgi1b;plxna2;myom1a;syngap1b;hoxc4a;lrrc39;fhl1a;sox11a;insm1a;pim1
GO:0072358	cardiovascular system development	1,838238818	0,00421576	0,16502121	dlc;vash2;syk;hey2;iqsec1b;crim1;aplnrb;notch2;nrxn3a;c1galt1a;npas4a;ackr3b;enox1;colec12;yjefn3;nrxn3b;dab2ipb;shc1;nlgn1;runx1;mtbl;itga4
GO:0048585	negative regulation of response to stimulus	1,855458193	0,00461044	0,16502121	fgfr1a;sema3b;ptch1;bcor;sh3bp4a;ek1;ephb2b;aplnrb;tle2a;sema3ga;arrb1;ret;serping1;sik1;tle3b;sesn2;dkk3b;dab2ipb;amer2;sox11a;inppl1a
GO:0010648	negative regulation of cell communication	1,94527376	0,00522955	0,16502121	fgfr1a;ptch1;bcor;sh3bp4a;ek1;ephb2b;aplnrb;tle2a;arrb1;ret;sik1;tle3b;sesn2;dkk3b;dab2ipb;amer2;sox11a;inppl1a
GO:0007163	establishment or maintenance of cell polarity	2,827364865	0,00702175	0,19941759	dlg1;llgl1;rhoub;rhoca;aplnrb;prickle1a;rnd2;cap2
GO:0040011	locomotion	1,575489458	0,00865666	0,20511549	nusap1;slc35b2;syk;sema3b;ek1;rhoca;mdga2a;tekt4;sst1.2;aplnrb;prickle1a;sema3ga;arrb1;tmsb;ret;ackr3b;srgap3;plxna2;rxfp3.3a1;celsr1a;chrm4a;rnd2;rhobtb1;rbpms2b;insm1a;vstm2l;efna3b;nsmfb;nr6a1a;inppl1a
GO:0035295	tube development	1,590022275	0,00866685	0,20511549	dlc;sox9a;foxa2;vash2;syk;mef2cb;hey2;iqsec1b;bcor;cdc42se1;atp2a2a;dnmt1;pcdh19;aplnrb;prickle1a;nrxn3a;slc8a4a;c1galt1a;ackr3b;colec12;yjefn3;nrxn3b;polr3b;dab2ipb;shc1;nlgn1;nr6a1a;mtbl;cldn15a

GO:0060537	muscle tissue development	2,196063931	0,01149092	0,24478239	mef2cb;fgfr1a;plod2;myl7;npr1a;aplnrb;slc8a4a;ret;myom1a;lrrc39;gfra3
GO:0030029	actin filament-based process	1,755863516	0,01206674	0,24478239	dlg1;llgl1;rhoub;iqsec1b;myl7;ek1;rhoca;arpc1b;tpma;gas2l3;tmsb;myom1a;rnd2;lrrc39;gas7a;rhobtb1;frmd5;cnn1b;cap2

KEGG pathways

geneSet	description	enrichmentRatio	pValue	FDR	userId
dre04270	Vascular smooth muscle contraction	2,420892909	0,00181354	0,22131854	adora2b;itpr1a;ppp1r14ab;npr1b;prkcbb;cacna1fb;adra1d;npr1a;adcy7;prkceb;itpr1b;calml4a;adcy3a;adcy5
dre04114	Oocyte meiosis	2,409696591	0,00273233	0,22131854	itpr1a;igf1ra;pgr;ccnb1;plk1;adcy7;cpeb3;ywhag1;itpr1b;calml4a;adcy3a;adcy5;igf1
