# Supplementary material for: A δ-cell subpopulation with a pro-β-cell identity contributes to efficient age-independent recovery in a zebrafish model of diabetes
Source: eLife. 2022 Jan 21;11:e67576. doi: 10.7554/eLife.67576 (PMC8820734; doi:10.7554/eLife.67576)
Supplement: Figure 3—source data 4. [file elife-67576-fig3-data4.pdf]

**Figure 3-Source Data 4. Biological Process and pathways overrepresented in β-cells**

GO identified by WebGestalt using the list of DE genes overexpressed in CTL βcells (versus 20dpt bihormonal cells) (DESeq FC>2x, Padj<0.05)
GO Padj (FDR) <0.25

**Biological Process (non redundant)**

| geneSet | description | enrichmentRatio | pValue | FDR | userId |
|---|---|---|---|---|---|
| GO:0032879 | regulation of localization | 2,58036999 | 3,56E-08 | 1,01E-05 | dpysl3;stxbp5a;rnd1b;plxna3;srgap1a;mmp14b;rab3c;panx1b;rhbdf1b;cacna1ba;klf6a;nexmifb;cacng2a;plxnb3;sulf1;arhgap4a;cxcr4b;anxa3b;kcnj8;kcnj11;sema4e;pparab;ccdc125;jph1a;bin1b;rims4;stim1a;ripor2;cplx2;pm20d1.2;kcnj19b;pllp;sema4ba;cacng3b;rnd3a;si:dkey-183c2.4;mcu;parietopsin;vegfaa |
| GO:0051674 | localization of cell | 2,113862421 | 1,02E-04 | 0,01061422 | dpysl3;esr1;rnd1b;fmnl3;plxna3;srgap1a;mmp14b;arpc5b;tnc;klf6a;nexmifb;plxnb3;cebpa;sulf1;arhgap4a;cxcr4b;anxa3b;sema4e;pld1a;ccdc125;alcamb;ripor2;met;sema4ba;cxcl20;pik3cb;rnd3a;si:dkey-183c2.4;vegfaa |
| GO:0098657 | import into cell | 2,709388489 | 1,12E-04 | 0,01061422 | gulp1a;vldlr;cacng2a;slc7a2;lgals3bpb;anxa3b;kcnj8;kcnj11;zgc:91818;slc6a4b;bin1b;slc12a9;hhipl1;kcnj19b;pllp;met;cacng3b;scarb2a |
| GO:1901264 | carbohydrate derivative transport | 6,947149972 | 1,67E-04 | 0,011870554 | slc25a24;slc25a25a;slc25a4;mfsd2aa;slc25a25b;slc29a4 |
| GO:0022610 | biological adhesion | 2,342748369 | 3,41E-04 | 0,019346833 | plxna3;cntn5;tnc;ctnna2;nexmifb;itga9;plxnb3;itga3a;lamb4;lgals3bpb;parvaa;anxa3b;clstn2;ripor2;dchs1a;si:ch211-74m13.3;thbs3a;si:dkey-183c2.4;epd;vegfaa |
| GO:0040011 | locomotion | 1,87396348 | 5,08E-04 | 0,024025397 | dpysl3;esr1;rnd1b;fmnl3;plxna3;srgap1a;mmp14b;arpc5b;tnc;klf6a;nexmifb;plxnb3;cebpa;sulf1;arhgap4a;cxcr4b;anxa3b;cyfip1;sema4e;pld1a;ccdc125;alcamb;ripor2;met;sema4ba;cxcl20;pik3cb;kif5c;rnd3a;si:dkey-183c2.4;vegfaa |
| GO:0006812 | cation transport | 1,833049719 | 0,001981178 | 0,080379225 | si:ch211-247n2.1;atp2b1b;micu2;panx1b;cax2;abcc9;cacna1ba;cacng2a;slc7a2;anxa3b;kcnj8;kcnj11;zgc:91818;slc6a4b;jph1a;slc25a47a;slc12a9;stim1a;pm20d1.2;kcnj19b;slc6a15;slc31a2;cacng3b;cox8b;mcu;ndufa4l2a |
| GO:0015748 | organophosphate ester transport | 3,763039568 | 0,00238089 | 0,084521592 | slc25a24;slc25a25a;slc25a4;mfsd2aa;slc25a25b;slc25a33;prelid3a |
| GO:0051336 | regulation of hydrolase activity | 1,89278637 | 0,003656415 | 0,09310942 | stxbp5a;plxna3;srgap1a;arhgap12a;agt;rcan2;cst14a.1;rasa4;plxnb3;rasgef1ba;tfpia;arhgap4a;rap1gap2a;ppp1r15a;tnfrsf1b;myo9aa;ppp1r14c;si:dkey-183c2.4;gchfr;vegfaa;cst14b.1 |
| GO:0071526 | semaphorin-plexin signaling pathway | 4,70379946 | 0,003762361 | 0,09310942 | plxna3;plxnb3;sema4e;sema4ba;si:dkey-183c2.4 |
| GO:0071875 | adrenergic receptor signaling pathway | 6,020863309 | 0,00379002 | 0,09310942 | adrb1;hrh3;gpr101;adra2b |
| GO:0009611 | response to wounding | 2,424508715 | 0,003934201 | 0,09310942 | dpysl3;fgf20a;dysf;gucy1a1;tnc;socs3a;tfpia;cxcr4b;ctsba;pik3cb;hcar1-4;lmo2 |
| GO:0022603 | regulation of anatomical structure morphogenesis | 2,022819689 | 0,004477354 | 0,097812966 | rnd1b;lypd6;fmnl3;plxna3;palm1b;ism1;tnc;plxnb3;sulf1;cxcr4b;cyfip1;sema4e;ripor2;sema4ba;rnd3a;si:dkey-183c2.4;vegfaa |
| GO:0032989 | cellular component morphogenesis | 1,688778733 | 0,009459357 | 0,191889809 | dpysl3;tmem59l;rnd1b;fmnl3;plxna3;palm1b;dnajb6b;tnc;ctnna2;plxnb3;mnx1;cxcr4b;elp3;parvaa;cyfip1;sema4e;alcamb;sema4ba;kif5c;myo9aa;rnd3a;si:dkey-183c2.4;vegfaa |
| GO:0098660 | inorganic ion transmembrane transport | 1,730133135 | 0,011797485 | 0,223365721 | si:ch211-247n2.1;atp2b1b;micu2;ttyh2l;cax2;abcc9;cacna1ba;cacng2a;kcnj8;kcnj11;jph1a;slc12a9;stim1a;pm20d1.2;kcnj19b;slc31a2;cacng3b;cox8b;mcu;ndufa4l2a |
| GO:0033993 | response to lipid | 2,263482447 | 0,012949478 | 0,225550479 | esr1;rorcb;nr1i2;esr2b;cxcr4b;jun;anxa3b;pparab;cxcl20;vdra |
| GO:0030522 | intracellular receptor signaling pathway | 3,010431655 | 0,014184152 | 0,225550479 | esr1;rorcb;nr1i2;esr2b;pparab;vdra |
| GO:0032409 | regulation of transporter activity | 3,420945062 | 0,014697831 | 0,225550479 | cacng2a;anxa3b;stim1a;pm20d1.2;cacng3b |
| GO:0040007 | growth | 1,858291145 | 0,015338373 | 0,225550479 | tmem59l;plxna3;fgf20a;mmp14b;tnc;klf6a;cxcr4b;cyfip1;sema4e;ctsba;mpzl1l;sema4ba;myo9aa;lmo2;ndrg4 |
| GO:0007264 | small GTPase mediated signal transduction | 1,843121421 | 0,016414535 | 0,225550479 | rnd1b;cyth3a;arhgef18a;rab3c;rasa4;rasgef1ba;eps8l2;rap1gap2a;ripor2;kalrnb;rnd3a;iqsec2b;rapgef3;ngfra;ksr2 |
| GO:1901700 | response to oxygen-containing compound | 2,006954436 | 0,016678028 | 0,225550479 | esr1;sesn3;sesn1;socs3a;ins;cxcr4b;jun;entpd1;cxcl20;vdra;vegfaa;gcgra |

| | | | | | |
|---|---|---|---|---|---|
| GO:0120036 | plasma membrane bounded cell projection organization | 1,551768894 | 0,018726031 | 0,238246687 | dpysl3;tmem59l;fmnl3;plxna3;ak5;tnc;ctnna2;socs3a;plxnb3;mnx1;cxcr4b;elp3;cyfip1;sema4e; dtnbp1a;eps8l2;alcamb;ripor2;map4l;sema4ba;kif5c;myo9aa;si:dkey-183c2.4;vegfaa;sept9b |
| GO:0010035 | response to inorganic substance | 2,778859989 | 0,020414727 | 0,238246687 | si:ch211-247n2.1;slc25a24;rasa4;jun;entpd1;pcxb |
| GO:0051240 | positive regulation of multicellular organismal process | 2,019191964 | 0,020582906 | 0,238246687 | plxna3;panx1b;tnc;plxnb3;ins;sulf1;anxa3b;clstn2;tnfrsf1b;si:dkey-183c2.4;vegfaa |
| GO:0051128 | regulation of cellular component organization | 1,535934518 | 0,02097242 | 0,238246687 | dpysl3;rnd1b;fmnl3;plxna3;palm1b;mmp14b;arpc5b;tnc;ctnna2;socs3a;plxnb3;vil1;cxcr4b; cyfip1;sema4e;eps8l2;bin1b;clstn2;ripor2;pllp;sema4ba;rnd3a;si:dkey-183c2.4;rassf7a;vegfaa |
| GO:0008283 | cell proliferation | 1,77084215 | 0,022691697 | 0,244356751 | btg2;ism1;tnc;tp53inp1;klf6a;sulf1;cdk6;plcd3b;lmo2;btc;vdra;parietopsin;si:ch211-260e23.9; vegfaa;ndrg4 |
| GO:2000026 | regulation of multicellular organismal development | 1,565709135 | 0,024315188 | 0,244356751 | dpysl3;lypd6;fam49a;plxna3;ism1;tnc;socs3a;maff;klf6a;nexmifb;plxnb3;ins;sulf1;cxcr4b; sema4e;clstn2;tnfrsf1b;sema4ba;twist1b;si:dkey-183c2.4;rflnb;vegfaa |
| GO:0014070 | response to organic cyclic compound | 2,27202389 | 0,024457375 | 0,244356751 | esr1;rorcb;nr1i2;esr2b;jun;anxa3b;pparab;vdra |
| GO:0051094 | positive regulation of developmental process | 2,020423929 | 0,026487893 | 0,244356751 | plxna3;tnc;plxnb3;ins;jun;clstn2;ripor2;tnfrsf1b;si:dkey-183c2.4;vegfaa |
| GO:0016192 | vesicle-mediated transport | 1,5178647 | 0,026556855 | 0,244356751 | gulp1a;stxbp5a;vldlr;rab3c;mfsd2aa;cacng2a;arl4cb;lgals3bpb;cnih2;anxa3b;dtnbp1a; zgc:91818;bin1b;rims4;kif1aa;cplx2;hhipl1;pllp;met;kif5c;cacng3b;si:ch211-284f22.3;scarb2a;arl4aa |
| GO:0031016 | pancreas development | 2,394661543 | 0,026672744 | 0,244356751 | klf6a;ins;mnx1;kcnj11;insm1b;met;nkx6.2 |

**KEGG pathways**

| **geneSet** | **description** | **enrichmentRatio** | **pValue** | **FDR** | **userId** |
|---|---|---|---|---|---|
| dre04142 | Lysosome | 3,090536124 | 8,97E-05 | 0,0145294 | ctsla;cd164;hexb;asah1a;lgmn;ctsh;ctsz;galns;si:ch211-122f10.4;ctsba;ctsd;nagpa;cln5; scarb2a;naga |
| dre04510 | Focal adhesion | 2,104447369 | 0,002804914 | 0,227197998 | egfra;vaspb;tnr;tnc;itga9;itga3a;lamb4;jun;parvaa;ccnd2a;met;pik3cb;thbs3a;hrasa;pdgfab; lamb1a;vegfaa |
