# Supplementary material for: A δ-cell subpopulation with a pro-β-cell identity contributes to efficient age-independent recovery in a zebrafish model of diabetes
Source: eLife. 2022 Jan 21;11:e67576. doi: 10.7554/eLife.67576 (PMC8820734; doi:10.7554/eLife.67576)
Supplement: Figure 3—source data 5. [file elife-67576-fig3-data5.docx]

**Figure 3-source data 1**

| Transcription factor | Expression in mouse/human islets  (mature/adults) | Zebrafish orthologue | Expression in zebrafish islets (mature/adults) | Functional orthologue/paralogue or equivalent in zebrafish |
| --- | --- | --- | --- | --- |
| *Neurod1* | Pan-endocrine | *neurod1* | Pan-endocrine |  |
| *Pax6* | Pan-endocrine | *pax6b* | Pan-endocrine | pax6b |
| *Isl1* | Pan-endocrine | *isl1* | Pan-endocrine |  |
| *Pdx1* | β-cells, δ-cells | *pdx1* | β-cells, sst1.1 δ-cell (this study) |  |
| *Nkx6.1* | β-cells | *nkx6.1* | Not expressed in mature islet cells | *nkx6.2* in β-cells |
| *Nkx6.2* | Not detected in mature islet cells | *nkx6.2* | β-cells |  |
| *Mnx1* | β-cells | *mnx1* | β-cells and α-cells |  |
| *Hhex* | δ-cells | *hhex* | δ-cells |  |

| Transcription factor | Expression in mouse/human pancreatic  progenitors | Zebrafish orthologue | Expression in pancreatic progenitors | Functional orthologue/paralogue or equivalent in zebrafish |
| --- | --- | --- | --- | --- |
| *Ascl1 (previously Mash1)* | Not expressed in mature islet cells | *ascl1b* | Not expressed in mature islet cells | *ascl1b* |
| *Neurog3* | Endocrine progenitors. Not expressed in mature islet cells | *neurog3* | No expression in zebrafish pancreas | *ascl1b and neurod1* in embryonic progenitors |
| *Nkx6.1* | Pancreatic embryonic progenitors  (ducts) | *nkx6.1* | Pancreatic embryonic progenitors and duct cells |  |
| *Sox9* | Pancreatic embryonic progenitors | *sox9b* | Pancreatic embryonic progenitors | *sox9b* |
| *Pdx1* | Pancreatic embryonic progenitors and duct cells | *Pdx1* | Pancreatic embryonic progenitors and duct cells |  |
