# Supplementary material for: A δ-cell subpopulation with a pro-β-cell identity contributes to efficient age-independent recovery in a zebrafish model of diabetes
Source: eLife. 2022 Jan 21;11:e67576. doi: 10.7554/eLife.67576 (PMC8820734; doi:10.7554/eLife.67576)
Supplement: Figure 4—source data 1. [file elife-67576-fig4-data1.pdf]

Figure 4-Source Data 1

Cell counts in the pancreatic tail (adult zebrafish)

Bihormonal mCherry+ GFP+ cells

CTL	20dpt
185	2810
123	691
288	1440
115	2446
54	1598

Monohormonal mCherry+ (GFP-) β-cells

CTL	20dpt
3946	109
2190	76
1570	72
989	50
651	52
