# Supplementary material for: A δ-cell subpopulation with a pro-β-cell identity contributes to efficient age-independent recovery in a zebrafish model of diabetes
Source: eLife. 2022 Jan 21;11:e67576. doi: 10.7554/eLife.67576 (PMC8820734; doi:10.7554/eLife.67576)
Supplement: Figure 4—source data 2. [file elife-67576-fig4-data2.pdf]

Figure 4-Source Data 2

Intraperitoneal Glucose Tolerance test (adults)

	CTL					
**Time (min)**						
0	55	58	57	87	59	69
60	394	368	380	371	439	456
180	119	202	231	81	410	232
240	92	66	234	63		

	NFP (regenerated)					
**Time (min)**						
0	65	67	118	111	102	142
60	364	271	372	459	489	467
180	82	74	91	100	158	222
240	103	91	78	119		

71	49	
370	420	489
143		

71	87	
409	462	
384	213	229
