# Supplementary material for: A δ-cell subpopulation with a pro-β-cell identity contributes to efficient age-independent recovery in a zebrafish model of diabetes
Source: eLife. 2022 Jan 21;11:e67576. doi: 10.7554/eLife.67576 (PMC8820734; doi:10.7554/eLife.67576)
Supplement: Figure 5—source data 1. [file elife-67576-fig5-data1.pdf]

Figure 5-Source Data 1. Significant differentially expressed genes between GFPhigh and GFPlow δ-cells

DESeq Padj<0.05

Ensembl_ID	Gene_Name	Mean_ CTL_GFPhigh	Mean_ CTL_GFPlow	log2FC	Padj
ENSDARG00000071209	oprl1	0	88	5,27	4,19E-04
ENSDARG00000006865	glra4a	1	180	5,20	1,36E-04
ENSDARG00000076280	ppp1r1b	1	355	5,12	1,13E-04
ENSDARG00000035120	gldc	2	319	5,08	5,87E-05
ENSDARG00000005616	bfb	1	218	5,04	3,30E-04
ENSDARG00000075757	gig2e	0	341	4,94	1,72E-04
ENSDARG00000059438	galnt18b	0	76	4,93	3,63E-04
ENSDARG00000075718	rpz5	5	905	4,92	6,58E-05
ENSDARG00000028346	tesca	0	83	4,84	4,76E-04
ENSDARG00000046054	CT027815.1	3	329	4,83	1,07E-04
ENSDARG00000091513	si:dkey-22o12.2	0	153	4,83	4,76E-04
ENSDARG00000044501	viml	0	79	4,82	1,45E-03
ENSDARG00000112049	cldn15a	2	459	4,82	1,07E-04
ENSDARG00000010266	igf2bp3	0	56	4,80	3,60E-04
ENSDARG00000022971	epha6	1	108	4,78	5,44E-04
ENSDARG00000015869	slc22a16	7	699	4,76	1,34E-04
ENSDARG00000052713	lrrtm1	15	782	4,76	2,18E-05
ENSDARG00000061365	hepacam2	15	1510	4,76	1,35E-04
ENSDARG00000089885	slc16a12b	13	1158	4,72	9,58E-05
ENSDARG00000051876	ush1c	4	286	4,72	4,75E-05
ENSDARG00000037159	oprd1b	11	1913	4,72	1,19E-04
ENSDARG00000099111	zgc:175280	0	61	4,72	1,10E-03
ENSDARG00000035562	mpdu1a	1	119	4,72	4,19E-04
ENSDARG00000103679	bmp16	5	899	4,72	1,14E-04
ENSDARG00000057833	gulp1b	26	2415	4,71	1,29E-04
ENSDARG00000044566	fabp6	5	1448	4,64	1,35E-04
ENSDARG00000087798	pdyn	17	2081	4,64	1,63E-04
ENSDARG00000099850	iqsec3a	0	113	4,63	6,53E-04
ENSDARG00000062478	ackr3a	0	75	4,62	5,37E-04
ENSDARG00000110069	pdgfaa	0	90	4,62	9,65E-04
ENSDARG00000090297	ldlrad2	0	60	4,62	6,02E-04
ENSDARG00000094511	ccl20b	3	988	4,60	1,94E-04
ENSDARG00000034718	tfpia	3	672	4,59	1,34E-04
ENSDARG00000007108	lipia	1	191	4,58	8,53E-04
ENSDARG00000035521	sfrp1a	1	95	4,58	1,20E-03
ENSDARG00000058961	ngfa	0	40	4,58	1,17E-03
ENSDARG00000100494	pbx1a	7	408	4,58	6,58E-05
ENSDARG00000068288	lamc2	4	870	4,57	3,29E-04
ENSDARG00000074723	myo10l1	0	62	4,55	4,80E-04
ENSDARG00000019856	atp1a1b	21	2290	4,53	3,80E-04
ENSDARG00000101637	ccnd1	7	286	4,53	3,66E-06
ENSDARG00000017591	fat1a	10	868	4,52	1,14E-04
ENSDARG00000016470	anxa5b	1	68	4,51	7,54E-04
ENSDARG00000070917	kitlga	1	146	4,51	7,54E-04
ENSDARG00000089790	efna5a	0	110	4,51	4,31E-04
ENSDARG00000031228	podxl	2	196	4,51	1,83E-04
ENSDARG00000027088	ptgdsb.1	0	107	4,49	1,42E-03
ENSDARG00000036456	anxa4	51	4267	4,49	1,29E-04
ENSDARG00000070110	opn5	0	71	4,49	8,46E-04
ENSDARG00000105153	ifitm5	3	499	4,48	3,40E-04
ENSDARG00000053542	kctd12.2	523	31726	4,48	2,34E-04
ENSDARG00000092520	si:dkey-250k15.10	0	70	4,48	1,20E-03
ENSDARG00000101481	rbp5	1	64	4,46	1,32E-04
ENSDARG00000068144	nmba	40	3309	4,46	1,36E-04
ENSDARG00000075542	zfhx4	5	269	4,46	1,14E-04
ENSDARG00000054239	ghrl	276	44119	4,46	1,68E-04
ENSDARG00000058243	phactr3a	0	36	4,46	1,16E-03
ENSDARG00000033614	rasgef1ba	57	2908	4,45	1,07E-04
ENSDARG00000002235	mmp14a	0	149	4,45	1,10E-03
ENSDARG00000020794	neurod6b	0	62	4,45	8,00E-04
ENSDARG00000102464	wnt5b	0	80	4,44	7,63E-04
ENSDARG00000096971	si:dkey-206p8.1	37	1674	4,44	9,27E-05
ENSDARG00000022531	ntn1b	0	55	4,44	1,34E-03
ENSDARG00000034643	fhl3a	1	86	4,43	3,63E-04
ENSDARG00000057209	plppr5a	4	363	4,43	1,07E-03
ENSDARG00000051975	cd99	11	471	4,42	8,69E-05
ENSDARG00000069250	uacaa	0	98	4,40	1,37E-03
ENSDARG00000087349	reep6	1	129	4,40	1,24E-03

ENSDARG00000105223	pmp22a	3	436	4,39	6,01E-04
ENSDARG00000116139	cdx1b	23	1929	4,38	4,19E-04
ENSDARG00000039577	ptk2bb	0	46	4,38	1,21E-03
ENSDARG00000054616	cldni	80	9670	4,38	4,79E-04
ENSDARG00000038794	zgc:113531	2	117	4,37	6,39E-04
ENSDARG00000070391	tspan4b	4	524	4,37	4,19E-04
ENSDARG00000077847	olfm2a	34	3151	4,37	5,60E-04
ENSDARG00000099260	PITPNC1	8	1015	4,36	4,76E-04
ENSDARG00000077573	tmprss13b	1	127	4,35	8,95E-04
ENSDARG00000053383	rprma	4	514	4,34	3,63E-04
ENSDARG00000093413	edil3a	5	899	4,34	4,76E-04
ENSDARG00000022727	epha2b	0	59	4,32	2,23E-03
ENSDARG00000061242	tuft1a	6	338	4,30	3,29E-04
ENSDARG00000074763	mrap2a	41	2567	4,30	5,42E-04
ENSDARG00000099184	map2k6	572	17482	4,30	2,08E-05
ENSDARG00000053358	basp1	11	778	4,30	3,59E-04
ENSDARG00000035253	npr3	2	263	4,29	3,58E-04
ENSDARG00000077817	cxxc4	3	396	4,29	7,32E-04
ENSDARG00000038424	si:dkey-8k3.2	2	480	4,28	1,86E-04
ENSDARG00000088157	smoc2	4	647	4,28	7,92E-04
ENSDARG00000021442	cdh11	4	830	4,27	4,76E-04
ENSDARG00000011163	sema3fa	8	703	4,25	8,47E-04
ENSDARG00000019260	dhrs9	0	65	4,25	5,74E-04
ENSDARG00000079484	kcnt1	44	2475	4,25	5,47E-04
ENSDARG00000112990	BX897729.1	1	66	4,24	7,46E-04
ENSDARG00000014190	sst2	26197	1625452	4,23	5,29E-04
ENSDARG00000092987	zmat4a	0	92	4,23	1,87E-03
ENSDARG00000031702	prkg1b	6	583	4,22	6,02E-04
ENSDARG00000043226	nfixa	0	34	4,21	1,80E-03
ENSDARG00000078853	arhgef19	0	47	4,20	1,45E-03
ENSDARG00000020708	mdkb	54	3492	4,18	6,73E-04
ENSDARG00000112993	efnb3a	2	106	4,18	8,95E-04
ENSDARG00000074865	frmpd3	1	70	4,18	1,11E-03
ENSDARG00000062510	bcl11ba	0	25	4,18	1,44E-03
ENSDARG00000039434	oprm1	0	28	4,17	1,11E-03
ENSDARG00000077465	sorcs2	11	604	4,17	5,39E-04
ENSDARG00000013310	map3k15	117	6810	4,17	6,86E-04
ENSDARG00000090253	zgc:113223	0	40	4,16	1,78E-03
ENSDARG00000093739	si:dkey-83m22.7	8	281	4,15	1,98E-04
ENSDARG00000062967	kcng4a	0	34	4,15	2,05E-03
ENSDARG00000003706	ryr2b	0	24	4,14	3,92E-03
ENSDARG00000018303	etv4	5	441	4,14	8,48E-04
ENSDARG00000076499	usp53b	0	21	4,14	1,30E-03
ENSDARG00000015404	arl3l2	3	276	4,14	9,26E-04
ENSDARG00000007383	kcnk6	0	48	4,14	1,43E-03
ENSDARG00000079198	usp13	0	37	4,13	1,48E-03
ENSDARG00000060846	cabp7b	3	191	4,12	7,93E-04
ENSDARG00000012449	si:ch211-169p10.1	1	56	4,12	7,46E-04
ENSDARG00000074591	eda	4	172	4,12	6,34E-04
ENSDARG00000008637	mttp	0	96	4,12	6,39E-04
ENSDARG00000099183	fkbp10a	2	426	4,12	6,39E-04
ENSDARG00000076182	stat1b	1	162	4,11	1,27E-03
ENSDARG00000075012	si:ch211-12n8.3	3	125	4,11	5,42E-04
ENSDARG00000059950	plxdc2	0	34	4,11	1,76E-03
ENSDARG00000099129	phlda1	0	35	4,11	1,44E-03
ENSDARG00000039974	rai14	3	141	4,10	3,63E-04
ENSDARG00000013921	frya	32	1389	4,09	6,02E-04
ENSDARG00000060711	sv2bb	13	689	4,09	7,56E-04
ENSDARG00000012397	eya4	6	366	4,07	5,93E-04
ENSDARG00000104370	esm1	1	102	4,07	1,61E-03
ENSDARG00000014792	lrrc4bb	2	132	4,06	7,05E-04
ENSDARG00000091726	aebp1	51	4546	4,06	1,24E-03
ENSDARG00000055349	pknox2	3	277	4,06	8,47E-04
ENSDARG00000009026	ank2a	19	801	4,06	6,67E-04
ENSDARG00000056619	arhgap42a	3	148	4,06	3,73E-04
ENSDARG00000091560	slc6a19a.2	1	77	4,06	9,57E-04
ENSDARG00000098024	si:dkey-262k9.2	10	357	4,06	2,29E-04
ENSDARG00000074695	mertka	11	1121	4,05	1,35E-03
ENSDARG00000016081	cldn15la	0	32	4,05	1,24E-03
ENSDARG00000006120	tbx2b	36	1577	4,05	7,78E-04
ENSDARG00000095184	si:ch73-52p7.1	0	34	4,05	1,76E-03
ENSDARG00000045528	fgd6	5	225	4,04	5,05E-04
ENSDARG00000004415	tcf7l2	1	68	4,04	2,30E-03

ENSDARG00000069431	slc26a4	1	88	4,03	1,70E-03
ENSDARG00000063437	wnt9a	6	498	4,02	8,06E-04
ENSDARG00000104665	kirrel3l	1	89	4,02	2,70E-03
ENSDARG00000014215	cdh13	7	403	4,01	1,43E-03
ENSDARG00000009567	spegb	60	2911	4,01	1,44E-03
ENSDARG00000071465	lrrc39	10	224	4,01	2,21E-05
ENSDARG00000099880	sp6	0	84	4,00	1,50E-03
ENSDARG00000101077	SCTR	1	68	4,00	2,47E-03
ENSDARG00000112040	CR774179.5	3	255	3,99	6,82E-04
ENSDARG00000032565	cacng2a	0	27	3,99	9,35E-04
ENSDARG00000079029	dhx32b	0	26	3,98	1,59E-03
ENSDARG00000061963	vrtn	0	38	3,98	2,07E-03
ENSDARG00000053129	carhsp1	73	2035	3,98	1,36E-04
ENSDARG00000074589	rin2	1	136	3,98	1,48E-03
ENSDARG00000070081	rspo3	0	40	3,98	3,43E-03
ENSDARG00000008019	mxg	0	29	3,97	5,07E-03
ENSDARG00000079862	kl	89	3627	3,97	1,11E-03
ENSDARG00000025254	s100a10b	11	534	3,97	8,01E-04
ENSDARG00000062341	wfs1a	0	73	3,97	2,21E-03
ENSDARG00000023963	tpm4a	5	277	3,96	1,06E-03
ENSDARG00000103286	kcnk4a	1	47	3,96	3,11E-03
ENSDARG00000059043	ntf3	9	706	3,95	3,29E-03
ENSDARG00000090064	TMEM179 (1 of ma	40	863	3,93	2,44E-05
ENSDARG00000079611	sema4c	13	512	3,93	6,05E-04
ENSDARG00000011066	glra3	0	29	3,92	2,47E-03
ENSDARG00000018263	pdia2	2	194	3,92	1,72E-03
ENSDARG00000051912	zgc:152945	0	30	3,92	5,04E-03
ENSDARG00000069290	bida	0	23	3,92	1,71E-03
ENSDARG00000093686	si:rp71-36a1.2	0	40	3,91	1,71E-03
ENSDARG00000094112	slc22a21	1	45	3,91	1,48E-03
ENSDARG00000096809	si:ch211-168b3.2	0	32	3,91	4,09E-03
ENSDARG00000040944	ntd5	1	193	3,89	1,39E-03
ENSDARG00000087165	si:dkeyp-69c1.7	0	28	3,89	2,93E-03
ENSDARG00000088140	hsd17b7	2	76	3,88	6,80E-04
ENSDARG00000037677	fgf24	2	245	3,88	2,54E-03
ENSDARG00000045638	slc13a1	0	38	3,87	9,84E-03
ENSDARG00000054177	adgra1a	8	181	3,87	8,94E-05
ENSDARG00000005841	tnni2a.2	2	98	3,87	2,42E-03
ENSDARG00000014804	cacna2d1a	2	75	3,86	4,28E-04
ENSDARG00000002197	pygl	2	146	3,86	2,28E-03
ENSDARG00000022165	mgst1.2	0	77	3,86	9,38E-04
ENSDARG00000112767	LO017951.1	0	27	3,85	1,58E-03
ENSDARG00000087394	tshz3a	13	761	3,84	2,78E-03
ENSDARG00000117145	notum1b	0	38	3,84	3,29E-03
ENSDARG00000097209	cox8b	16	457	3,83	3,47E-04
ENSDARG00000012019	glra1	4	395	3,83	2,05E-03
ENSDARG00000008140	kcnab1a	0	35	3,83	1,58E-03
ENSDARG00000037917	itga3a	3	133	3,83	1,08E-03
ENSDARG00000044048	prnpb	4	239	3,82	1,52E-03
ENSDARG00000018757	klf5l	2	59	3,82	9,90E-04
ENSDARG00000101629	CABZ01079192.1	51	1671	3,82	1,11E-03
ENSDARG00000061692	yjefn3	84	1855	3,82	1,34E-04
ENSDARG00000060200	CU019662.1	3	269	3,82	1,96E-03
ENSDARG00000073935	wnt6b	0	34	3,82	4,37E-03
ENSDARG00000074508	si:dkey-28e7.3	1	27	3,82	4,27E-03
ENSDARG00000007941	hmx4	3	487	3,82	1,11E-03
ENSDARG00000076853	areg	1	62	3,82	1,89E-03
ENSDARG00000086623	vav3a	1	63	3,82	3,28E-03
ENSDARG00000008541	chia.4	0	25	3,81	5,38E-03
ENSDARG00000035715	marcksl1b	183	4679	3,81	3,63E-04
ENSDARG00000023369	mxd	0	24	3,81	4,14E-03
ENSDARG00000053272	si:dkey-166d12.2	11	257	3,81	3,08E-04
ENSDARG00000059601	map1aa	20	506	3,81	2,61E-04
ENSDARG00000059442	smtnb	0	37	3,80	3,34E-03
ENSDARG00000079393	tmprss15	0	27	3,79	2,32E-03
ENSDARG00000029497	tfcp2l1	5	172	3,79	1,10E-03
ENSDARG00000011618	slc26a2	42	908	3,79	1,35E-04
ENSDARG00000052948	zgc:195023	85	6762	3,78	1,48E-03
ENSDARG00000100446	si:ch211-286o17.1	1	98	3,78	2,41E-03
ENSDARG00000045685	cntn1b	9	240	3,78	6,37E-04
ENSDARG00000021137	adgrv1	8	397	3,78	1,08E-03
ENSDARG00000104089	p3h4	22	1194	3,78	2,80E-03
ENSDARG00000060941	ANKFN1	1	51	3,78	4,84E-03

ENSDARG00000105324	si:dkey-11o1.7	0	20	3,77	6,07E-03
ENSDARG00000113737	GABRA2	1	109	3,77	2,09E-03
ENSDARG00000007490	adrb1	2	141	3,77	2,56E-03
ENSDARG00000023712	mao	13	391	3,77	6,39E-04
ENSDARG00000024546	pla2g4aa	0	37	3,76	2,81E-03
ENSDARG00000090499	tmem121ab	0	23	3,76	4,43E-03
ENSDARG00000092810	stap2a	0	61	3,75	3,60E-03
ENSDARG00000015506	klf5a	0	37	3,74	3,76E-03
ENSDARG00000078650	kcna4	1	35	3,73	6,42E-03
ENSDARG00000087084	hcar1-4	10	338	3,73	1,74E-03
ENSDARG00000040048	GALNTL6	9	545	3,73	3,88E-03
ENSDARG00000079293	gdpd4b	2	69	3,73	1,14E-03
ENSDARG00000079756	peak1	1	50	3,72	4,63E-03
ENSDARG00000057035	stoml3b	5	373	3,72	4,81E-03
ENSDARG00000045525	tmcc3	3	108	3,72	1,08E-03
ENSDARG00000052494	pcdh18b	1	135	3,72	5,57E-03
ENSDARG00000059348	rxfp3.3b	2	188	3,71	4,14E-03
ENSDARG00000059948	si:dkey-121b10.7	1	73	3,71	3,93E-03
ENSDARG00000101919	foxj1a	1	39	3,71	3,19E-03
ENSDARG00000059362	cavin1b	8	296	3,70	1,83E-03
ENSDARG00000014246	jag2a	0	41	3,70	2,62E-03
ENSDARG00000019516	sp7	1	55	3,69	9,91E-03
ENSDARG00000029832	slc26a1	9	177	3,69	1,94E-04
ENSDARG00000005595	adgb	1	60	3,69	3,92E-03
ENSDARG00000102138	foxa1	2	79	3,68	1,34E-03
ENSDARG00000078163	hpse2	0	45	3,68	7,66E-03
ENSDARG00000034504	lmo1	22	657	3,68	1,11E-03
ENSDARG00000078624	arhgef9b	0	53	3,68	5,25E-03
ENSDARG00000103607	wtip	1	39	3,67	9,63E-03
ENSDARG00000070735	rnd2	18	377	3,67	4,25E-04
ENSDARG00000075467	shisa3	1	113	3,66	2,27E-03
ENSDARG00000055825	celsr3	28	802	3,66	1,20E-03
ENSDARG00000007369	tcf7l1b	2	125	3,66	2,62E-03
ENSDARG00000020136	ptges	0	43	3,65	5,86E-03
ENSDARG00000020764	slmapb	78	1897	3,65	1,11E-03
ENSDARG00000057479	hrh2b	3	144	3,65	1,19E-03
ENSDARG00000078898	pcdh7a	0	31	3,65	9,52E-03
ENSDARG00000087736	si:dkey-118k5.3	1	93	3,65	4,92E-03
ENSDARG00000011434	ptger2a	31	1443	3,64	4,72E-03
ENSDARG00000101812	zgc:114120	33	651	3,64	4,25E-04
ENSDARG00000044632	myo7ab	8	219	3,64	8,47E-04
ENSDARG00000003750	pfas	35	782	3,63	6,39E-04
ENSDARG00000043864	nptnb	2	61	3,63	3,01E-03
ENSDARG00000018820	flncb	1	44	3,63	2,60E-03
ENSDARG00000069540	si:dkey-30c15.2	1	282	3,63	3,06E-03
ENSDARG00000091503	FADS6	2	98	3,62	2,39E-03
ENSDARG00000007950	itga11b	17	381	3,62	8,47E-04
ENSDARG00000015176	ror1	0	33	3,62	7,84E-03
ENSDARG00000100876	sez6a	0	60	3,61	2,02E-03
ENSDARG00000114444	CABZ01079081.1	2	93	3,61	3,14E-03
ENSDARG00000041107	cftr	1	49	3,61	6,12E-03
ENSDARG00000062672	kcnj19b	69	1186	3,61	1,09E-04
ENSDARG00000059746	plod1a	20	416	3,59	4,52E-04
ENSDARG00000042390	syndig1l	1	100	3,59	5,78E-03
ENSDARG00000056499	ca6	3	896	3,59	1,45E-03
ENSDARG00000006468	grap2a	0	28	3,59	7,16E-03
ENSDARG00000086815	MYADM	3	72	3,58	1,27E-03
ENSDARG00000099535	si:ch73-379j16.2	1	32	3,58	1,59E-03
ENSDARG00000067976	ar	1	122	3,58	3,36E-03
ENSDARG00000104868	si:dkey-21e5.1	0	25	3,57	4,11E-03
ENSDARG00000099411	zgc:158343	0	39	3,57	9,30E-04
ENSDARG00000090081	si:dkey-1m11.5	0	29	3,56	6,08E-03
ENSDARG00000077540	f2rl1.2	11	259	3,55	1,45E-03
ENSDARG00000097372	si:ch211-139d20.3	1	49	3,55	5,16E-03
ENSDARG00000039436	il13ra2	0	42	3,55	3,62E-03
ENSDARG00000035468	slc25a25b	11	231	3,54	4,19E-04
ENSDARG00000051768	fhod1	10	164	3,54	1,35E-04
ENSDARG00000101677	chrnb4	4	162	3,54	7,43E-03
ENSDARG00000039243	zgc:152791	2	124	3,53	5,69E-03
ENSDARG00000017446	camk1db	2	71	3,53	3,89E-03
ENSDARG00000078839	si:dkey-28o19.1	0	20	3,53	9,27E-03
ENSDARG00000057610	cdkn2c	2	49	3,53	1,21E-03
ENSDARG00000037879	lfng	2	93	3,53	1,66E-03

ENSDARG00000039490	pitpnaa	390	6528	3,53	1,76E-04
ENSDARG00000004618	stx2a	39	559	3,53	2,18E-05
ENSDARG00000011407	col2a1b	25	521	3,52	8,47E-04
ENSDARG00000073824	RASGRF1	1	30	3,52	1,45E-02
ENSDARG00000099898	si:ch211-27e6.1	0	20	3,51	7,33E-03
ENSDARG00000035609	mtnr1c	17	352	3,50	1,22E-03
ENSDARG00000013174	nkd3	0	28	3,50	3,56E-03
ENSDARG00000056530	cpamd8	73	3682	3,49	4,82E-03
ENSDARG00000053380	fam189a1	1	128	3,49	4,80E-03
ENSDARG00000014927	uts1	14	925	3,49	4,26E-03
ENSDARG00000012860	tmprss4b	2	37	3,49	1,45E-03
ENSDARG00000014910	panx1b	3	62	3,49	3,08E-04
ENSDARG00000095048	si:dkey-250k15.7	20	483	3,48	3,70E-03
ENSDARG00000063713	syngap1a	2	84	3,48	4,86E-03
ENSDARG00000025667	adgrb2	1	50	3,48	1,83E-03
ENSDARG00000022895	mcf2a	28	738	3,48	3,29E-03
ENSDARG00000070617	vhl	41	625	3,48	1,13E-04
ENSDARG00000005141	camkvb	24	852	3,48	4,47E-03
ENSDARG00000077643	lypd6b	3	108	3,48	3,92E-03
ENSDARG00000001234	map4k2	1	35	3,48	5,84E-03
ENSDARG00000070618	tatdn2	6	140	3,47	2,76E-03
ENSDARG00000037267	zgc:158263	6	141	3,47	1,42E-03
ENSDARG00000098573	sgcd	11	580	3,47	7,67E-03
ENSDARG00000003615	slc26a3.2	427	6565	3,46	1,94E-04
ENSDARG00000109686	si:ch211-59p23.1	0	25	3,46	7,58E-03
ENSDARG00000116353	CABZ01071171.1	1	46	3,46	1,01E-02
ENSDARG00000074307	rapgef5b	0	25	3,45	6,28E-03
ENSDARG00000101980	bmper	3	82	3,45	2,73E-03
ENSDARG00000076500	chgb	18	320	3,45	7,07E-04
ENSDARG00000001968	dock5	2	55	3,44	3,50E-03
ENSDARG00000103736	gramd2aa	1	37	3,44	5,02E-03
ENSDARG00000031712	grm3	4	165	3,44	7,05E-03
ENSDARG00000010862	amt	12	213	3,44	8,82E-04
ENSDARG00000060837	trpc5b	17	636	3,43	1,05E-02
ENSDARG00000090063	fa2h	11	259	3,42	3,29E-03
ENSDARG00000007823	atf3	44	722	3,42	3,49E-04
ENSDARG00000062129	mxra8b	1	40	3,42	9,77E-03
ENSDARG00000007723	efnb1	5	97	3,41	2,00E-03
ENSDARG00000032435	itga9	2	45	3,41	2,16E-03
ENSDARG00000027957	fgf12a	8	285	3,40	7,66E-03
ENSDARG00000010565	aqp4	133	4867	3,40	8,02E-03
ENSDARG00000017354	epha2a	0	78	3,40	3,54E-03
ENSDARG00000020250	rhoj	0	25	3,40	1,06E-02
ENSDARG00000088202	sh3d19	8	146	3,39	1,02E-03
ENSDARG00000101495	ugt5b3	2	43	3,39	3,58E-03
ENSDARG00000014273	camk2d2	2	62	3,39	4,87E-03
ENSDARG00000063230	bmp7b	9	480	3,39	9,07E-03
ENSDARG00000053301	insm1b	19	354	3,39	8,15E-04
ENSDARG00000069143	map3k7cl	3	76	3,37	4,88E-03
ENSDARG00000102995	rbm24a	5	397	3,37	3,11E-03
ENSDARG00000016837	glipr2l	1	42	3,37	3,89E-03
ENSDARG00000103950	pcdh1gc6	16	240	3,36	5,14E-04
ENSDARG00000036292	cdx4	11	274	3,36	5,52E-03
ENSDARG00000069954	kcnq5a	0	22	3,36	1,75E-02
ENSDARG00000075277	si:dkeyp-14d3.1	1	60	3,36	6,82E-03
ENSDARG00000097889	si:ch73-265h17.2	2	52	3,36	5,71E-03
ENSDARG00000071879	tgfb2l	2	69	3,36	1,06E-02
ENSDARG00000044010	loxl2a	21	793	3,36	5,81E-03
ENSDARG00000055229	ncs1a	1	45	3,36	1,75E-02
ENSDARG00000090202	rbm11	0	59	3,35	6,63E-03
ENSDARG00000088251	si:ch211-24o10.6	11	240	3,35	3,01E-03
ENSDARG00000020292	si:ch211-254n4.3	2	174	3,34	6,28E-03
ENSDARG00000073802	cntnap5l	3	208	3,34	8,99E-03
ENSDARG00000100731	CU372926.1	1	25	3,33	7,85E-03
ENSDARG00000071107	wnt7bb	19	399	3,33	2,91E-03
ENSDARG00000074760	ttc7a	15	284	3,33	3,76E-03
ENSDARG00000077470	si:dkey-193b15.8	0	25	3,33	1,22E-02
ENSDARG00000037363	st5	28	484	3,32	2,39E-03
ENSDARG00000006308	ptpn18	1	28	3,32	9,89E-03
ENSDARG00000104847	ptger1b	4	365	3,32	1,06E-02
ENSDARG00000060753	pear1	4	336	3,31	7,47E-03
ENSDARG00000042112	dio1	2	38	3,30	4,14E-03
ENSDARG00000015025	l1camb	6	139	3,30	6,75E-03

ENSDARG00000006775	cass4	1	21	3,29	1,64E-02
ENSDARG00000060884	nmur1a	1	51	3,29	1,38E-02
ENSDARG00000101577	si:ch211-172l8.4	0	25	3,28	1,37E-02
ENSDARG00000077722	ppp2r3a	1	61	3,28	9,91E-03
ENSDARG00000076981	zgc:198329	1	29	3,28	1,05E-02
ENSDARG00000074908	col6a1	0	33	3,28	1,63E-02
ENSDARG00000089368	hopx	267	3164	3,28	4,72E-05
ENSDARG00000013837	spo11	2	54	3,27	1,15E-02
ENSDARG00000002634	b4galt1	0	29	3,27	7,71E-03
ENSDARG00000076690	avpr2l	4	418	3,26	1,10E-02
ENSDARG00000027419	gad1b	1	23	3,26	1,66E-02
ENSDARG00000014626	dlx3b	2	42	3,26	1,53E-02
ENSDARG00000059707	ZNF423	4	72	3,26	2,02E-03
ENSDARG00000031783	adcy8	4	77	3,26	5,58E-03
ENSDARG00000060152	fam155b	0	34	3,26	5,81E-03
ENSDARG00000099236	sept-10	9	161	3,25	2,81E-03
ENSDARG00000031095	veph1	0	26	3,25	1,86E-02
ENSDARG00000088878	c1qtnf1	3	89	3,25	7,07E-03
ENSDARG00000016981	bcar3	4	139	3,25	8,66E-03
ENSDARG00000042548	tpd52l1	18	329	3,25	4,41E-03
ENSDARG00000078177	cxcr3.1	3	54	3,25	8,16E-03
ENSDARG00000055283	id2a	14	194	3,24	6,58E-04
ENSDARG00000080009	bahcc1b	4	192	3,24	1,03E-02
ENSDARG00000005800	ampd3a	8	157	3,24	8,03E-03
ENSDARG00000063634	hpse	7	102	3,24	1,43E-03
ENSDARG00000004635	epha7	31	658	3,24	8,93E-03
ENSDARG00000043646	slc6a8	0	71	3,24	2,23E-03
ENSDARG00000010083	rbfox3a	3	61	3,24	2,63E-03
ENSDARG00000009544	cldnb	1	118	3,23	3,22E-03
ENSDARG00000039181	trpm3	28	566	3,23	8,57E-03
ENSDARG00000063224	nxph2a	0	21	3,23	3,33E-02
ENSDARG00000075189	cntnap5b	1	102	3,23	5,87E-03
ENSDARG00000045443	agtr1b	26	993	3,22	1,72E-02
ENSDARG00000061047	abcg4a	4	159	3,22	1,94E-02
ENSDARG00000098769	slitrk6	2	185	3,21	8,68E-03
ENSDARG00000053475	ngb	0	26	3,21	2,18E-02
ENSDARG00000052898	kcnk3b	250	3370	3,21	9,79E-04
ENSDARG00000091459	gabra2a	1	164	3,21	3,87E-03
ENSDARG00000045749	ppfibp1a	52	731	3,21	1,21E-03
ENSDARG00000099387	jpt1a	3	58	3,21	5,08E-03
ENSDARG00000053179	cttnbp2nla	1	56	3,21	1,10E-02
ENSDARG00000098420	SNTA1	11	382	3,21	1,48E-02
ENSDARG00000017676	mmp2	3	274	3,20	7,66E-03
ENSDARG00000010420	ndrg1b	2	62	3,20	7,71E-03
ENSDARG00000098899	zmp:0000001082	15	587	3,20	1,44E-02
ENSDARG00000054683	prdm8b	3	42	3,19	3,87E-03
ENSDARG00000030867	opn7c	2	118	3,19	5,08E-03
ENSDARG00000040526	s1pr5a	0	85	3,19	3,06E-03
ENSDARG00000104569	CABZ01088484.1	28	791	3,19	1,31E-02
ENSDARG00000068100	fam19a5l	4	73	3,18	7,75E-03
ENSDARG00000045544	hgfa	0	24	3,18	1,65E-02
ENSDARG00000020031	cldn11a	30	439	3,18	2,07E-03
ENSDARG00000100847	esrrb	2	38	3,17	9,91E-03
ENSDARG00000042840	eml1	3	105	3,17	1,05E-02
ENSDARG00000027360	si:ch211-106h11.3	0	22	3,16	1,94E-02
ENSDARG00000071091	chrm3a	5	249	3,16	1,54E-02
ENSDARG00000077677	pdgfd	4	99	3,16	2,18E-02
ENSDARG00000105190	rhbdl3	1	106	3,15	7,78E-03
ENSDARG00000104213	si:dkey-283b1.6	4	61	3,15	2,64E-03
ENSDARG00000062156	abi3bpa	1	37	3,15	2,53E-02
ENSDARG00000091111	si:ch211-15b10.6	8	140	3,14	5,36E-03
ENSDARG00000089529	desi1b	3	38	3,14	1,27E-03
ENSDARG00000115189	LO017791.1	22	840	3,14	1,91E-02
ENSDARG00000060298	nin	53	617	3,14	3,90E-04
ENSDARG00000044485	sall4	37	719	3,14	5,52E-03
ENSDARG00000034080	plcd1b	3	58	3,14	8,33E-03
ENSDARG00000087616	maptb	66	700	3,13	5,41E-05
ENSDARG00000096567	si:ch211-155o21.4	2	72	3,13	9,14E-03
ENSDARG00000033735	ncf1	14	289	3,13	5,76E-03
ENSDARG00000103591	dkk3a	0	54	3,13	1,12E-02
ENSDARG00000032820	rxfp2a	41	482	3,13	4,19E-04
ENSDARG00000023053	fam129ab	1	38	3,13	1,09E-02
ENSDARG00000097869	si:dkey-1m11.6	0	31	3,13	1,02E-02

ENSDARG00000088885	si:ch1073-340i21.3	0	22	3,12	2,33E-02
ENSDARG00000089441	si:ch211-105c13.3	1	37	3,12	2,80E-02
ENSDARG00000036065	mgat1b	9	110	3,12	3,33E-04
ENSDARG00000045747	cped1	1	88	3,12	1,02E-02
ENSDARG00000061051	brinp3a.2	0	26	3,11	1,05E-02
ENSDARG00000077740	b3glctb	1	30	3,11	1,90E-02
ENSDARG00000067958	sh3gl1a	4	63	3,11	3,32E-03
ENSDARG00000052997	sema4e	1	25	3,11	7,31E-03
ENSDARG00000090963	atp6ap1lb	8	211	3,11	1,48E-02
ENSDARG00000103333	baiap2b	4	64	3,11	2,19E-03
ENSDARG00000098057	dscaml1	25	452	3,10	1,45E-02
ENSDARG00000071331	ryr3	60	780	3,10	1,99E-03
ENSDARG00000076856	frem2a	11	261	3,10	8,01E-03
ENSDARG00000097285	si:ch211-147g22.5	1	25	3,10	1,63E-02
ENSDARG00000093279	si:dkey-11o1.3	4	59	3,09	1,16E-02
ENSDARG00000076685	wisp1b	0	25	3,08	1,27E-02
ENSDARG00000079876	CU855878.1	16	222	3,08	1,85E-03
ENSDARG00000004445	grm5a	1	79	3,08	1,44E-02
ENSDARG00000070491	hpcal4	19	243	3,08	1,84E-03
ENSDARG00000100055	tox2	21	292	3,07	2,98E-03
ENSDARG00000018653	acot7	19	236	3,07	1,48E-03
ENSDARG00000021633	sh3bgr	3	49	3,07	5,33E-03
ENSDARG00000059347	calml4b	0	25	3,07	1,32E-02
ENSDARG00000068557	htr5ab	11	164	3,06	8,93E-03
ENSDARG00000100342	zgc:158464	1	22	3,06	9,91E-03
ENSDARG00000004932	anos1b	2	64	3,06	1,00E-02
ENSDARG00000046053	slc27a6	1	21	3,06	2,35E-02
ENSDARG00000090338	fam20cb	13	175	3,05	3,39E-03
ENSDARG00000006604	nectin3b	6	105	3,05	8,99E-03
ENSDARG00000015890	mafa	0	27	3,05	5,47E-03
ENSDARG00000069669	adra2c	0	30	3,05	1,80E-02
ENSDARG00000010183	si:ch73-265h17.1	7	94	3,04	6,30E-03
ENSDARG00000087446	mmd2a	7	88	3,04	3,16E-03
ENSDARG00000116804	CABZ01037174.1	7	225	3,04	2,43E-02
ENSDARG00000052157	il12rb2	4	56	3,04	5,74E-03
ENSDARG00000104125	ttc6	17	316	3,04	1,48E-02
ENSDARG00000038010	rac2	2	34	3,03	6,07E-03
ENSDARG00000015955	cldnc	0	89	3,03	2,60E-03
ENSDARG00000058821	sema5a	6	106	3,02	1,05E-02
ENSDARG00000055238	kif17	1	24	3,02	1,71E-02
ENSDARG00000060008	hhipl2	6	71	3,01	3,66E-03
ENSDARG00000006396	nrcama	196	2247	3,01	1,69E-03
ENSDARG00000016494	ddc	6	140	3,01	2,02E-02
ENSDARG00000010132	dacha	1	32	3,01	1,60E-02
ENSDARG00000056653	fhl1b	24	438	3,01	1,59E-02
ENSDARG00000044441	si:ch73-194h10.2	1	26	3,01	1,41E-02
ENSDARG00000078185	pacs2	2	124	3,00	1,18E-02
ENSDARG00000010727	ttyh2l	4	64	3,00	7,65E-03
ENSDARG00000088842	KCNB2	0	53	2,99	5,92E-03
ENSDARG00000074223	MDFIC	3	149	2,99	2,90E-02
ENSDARG00000061231	tinagl1	0	76	2,99	3,72E-03
ENSDARG00000000857	mapk14a	329	3062	2,99	6,58E-05
ENSDARG00000087709	FAT3 (1 of many)	3	288	2,99	9,00E-03
ENSDARG00000009624	UTS2R	0	122	2,99	5,78E-03
ENSDARG00000044694	fybb	1	39	2,98	1,46E-02
ENSDARG00000034453	unc119a	1	32	2,98	1,81E-02
ENSDARG00000106669	CABZ01068358.1	7	85	2,98	1,60E-03
ENSDARG00000024278	adh8b	25	289	2,97	2,52E-03
ENSDARG00000040430	nptxra	2	114	2,97	2,12E-02
ENSDARG00000074126	ttc39a	10	169	2,97	1,39E-02
ENSDARG00000077590	arsj	0	26	2,97	4,88E-02
ENSDARG00000076564	hspg2	33	467	2,97	5,86E-03
ENSDARG00000039220	st6galnac5a	22	467	2,96	2,83E-02
ENSDARG00000029905	phyhd1	5	77	2,96	1,54E-02
ENSDARG00000106606	CT027674.1	1	21	2,96	3,31E-02
ENSDARG00000102805	cyp2aa11	0	20	2,96	2,52E-02
ENSDARG00000045483	lrtm2a	5	75	2,95	6,15E-03
ENSDARG00000103712	plpp7	10	105	2,95	4,76E-04
ENSDARG00000073728	gal3st3	5	110	2,94	1,72E-02
ENSDARG00000115359	CABZ01077555.1	6	73	2,93	2,68E-03
ENSDARG00000036848	slc43a2a	734	7931	2,92	1,96E-03
ENSDARG00000025478	gipr	0	195	2,92	9,80E-03
ENSDARG00000113411	lrrc4c	1	32	2,91	2,63E-02

ENSDARG00000051899	exoc3l1	2	37	2,91	1,22E-02
ENSDARG00000013749	slc25a55b	1	22	2,91	2,14E-02
ENSDARG00000094747	si:ch211-197g15.6	2	60	2,91	2,56E-02
ENSDARG00000052376	b3gnt2a	0	26	2,91	1,16E-02
ENSDARG00000007412	slc2a1b	1	20	2,91	2,44E-02
ENSDARG00000104139	atp1a3b	3	121	2,90	5,16E-03
ENSDARG00000013926	slc16a9a	2	42	2,90	6,74E-03
ENSDARG00000037455	ugt8	83	786	2,90	5,61E-04
ENSDARG00000044142	acss1	5	57	2,90	6,53E-03
ENSDARG00000043716	cldn5a	1	30	2,89	2,28E-02
ENSDARG00000041609	adarb1a	26	265	2,89	1,55E-03
ENSDARG00000006602	chrna2a	2	36	2,89	2,06E-02
ENSDARG00000094836	si:ch211-195b15.8	292	2628	2,89	2,29E-04
ENSDARG00000091821	smc6	2	40	2,88	2,04E-02
ENSDARG00000070487	zgc:110783	93	929	2,88	1,54E-03
ENSDARG00000069356	adgrl2a	2	38	2,88	3,01E-02
ENSDARG00000114920	TTC28	4	71	2,88	1,11E-02
ENSDARG00000074680	rims1a	13	215	2,87	1,77E-02
ENSDARG00000103026	p3h2	2	33	2,87	1,80E-02
ENSDARG00000076236	lrrc18b	18	228	2,86	1,89E-02
ENSDARG00000021948	tnc	156	1579	2,86	2,05E-03
ENSDARG00000098129	si:dkeyp-97a10.2	8	93	2,86	6,82E-03
ENSDARG00000017329	cdkl1	16	199	2,86	7,18E-03
ENSDARG00000052652	fermt1	1	20	2,86	3,38E-02
ENSDARG00000078671	cdk5r2b	81	721	2,86	4,19E-04
ENSDARG00000016598	ckmt1	8	104	2,86	6,95E-03
ENSDARG00000041998	zgc:113337	1	41	2,86	4,31E-02
ENSDARG00000010407	ppp4r4	13	146	2,85	4,81E-03
ENSDARG00000034268	slit3	11	152	2,84	1,71E-02
ENSDARG00000075557	apcdd1l	19	388	2,84	3,97E-02
ENSDARG00000002084	lamb2	151	1665	2,84	7,65E-03
ENSDARG00000057107	si:ch73-383g2.1	1	31	2,83	1,76E-02
ENSDARG00000016011	gpcpd1	119	1105	2,83	1,42E-03
ENSDARG00000070100	si:ch211-168d1.3	9	129	2,83	1,47E-02
ENSDARG00000101368	cngb3.2	1	29	2,83	3,72E-02
ENSDARG00000103311	FBLN1	4	149	2,83	1,72E-02
ENSDARG00000070857	si:dkey-32e6.6	1	35	2,82	3,33E-02
ENSDARG00000056101	kcnd3	42	563	2,82	1,24E-02
ENSDARG00000061177	mov10b.1	11	141	2,82	1,05E-02
ENSDARG00000059226	zbtb8a	3	30	2,82	3,00E-03
ENSDARG00000042725	cebpb	35	509	2,81	7,48E-03
ENSDARG00000076870	piezo1	5	86	2,80	1,83E-02
ENSDARG00000061682	bicc1a	7	91	2,80	1,18E-02
ENSDARG00000005112	cdh17	5	116	2,80	2,27E-02
ENSDARG00000013072	mmp15b	15	322	2,80	4,11E-02
ENSDARG00000060639	apba2b	34	297	2,79	8,15E-04
ENSDARG00000052336	ociad2	6	64	2,79	3,07E-03
ENSDARG00000062797	nell2b	13	122	2,79	7,46E-04
ENSDARG00000007788	atp2b1b	52	486	2,79	1,78E-03
ENSDARG00000090537	ifit11	8	120	2,79	1,57E-02
ENSDARG00000033466	tagln2	11	182	2,79	2,65E-02
ENSDARG00000025859	lmf2b	18	159	2,79	7,94E-04
ENSDARG00000034307	chrne	4	63	2,78	2,45E-02
ENSDARG00000043531	jun	485	4194	2,78	6,53E-04
ENSDARG00000076030	cacnb3b	3	40	2,77	2,45E-02
ENSDARG00000054619	fras1	2	32	2,77	1,99E-02
ENSDARG00000069262	erich3	9	75	2,76	6,73E-04
ENSDARG00000002787	tle3a	37	303	2,76	3,52E-04
ENSDARG00000031136	moxd1	0	22	2,76	3,33E-02
ENSDARG00000062707	plat	2	32	2,76	1,49E-02
ENSDARG00000008200	ugp2b	448	3460	2,76	9,50E-05
ENSDARG00000074201	flna	76	896	2,75	1,14E-02
ENSDARG00000013784	zgc:77158	320	2490	2,75	1,36E-04
ENSDARG00000110496	zgc:162989	13	107	2,75	8,47E-04
ENSDARG00000088828	paqr8	0	22	2,75	2,06E-02
ENSDARG00000023724	cdc42se1	175	1369	2,74	2,29E-04
ENSDARG00000080018	kif16bb	4	40	2,74	6,40E-03
ENSDARG00000013613	anxa13l	34	314	2,73	2,73E-03
ENSDARG00000044718	vav2	5	60	2,73	1,62E-02
ENSDARG00000100886	si:dkeyp-97a10.1	12	132	2,73	9,17E-03
ENSDARG00000087131	ifnlr1	1	24	2,73	3,70E-02
ENSDARG00000036846	anks4b	2	24	2,72	1,81E-02
ENSDARG00000015557	zgc:100829	1	20	2,72	2,51E-02

ENSDARG00000036427	slc3a2a	958	7025	2,72	3,26E-05
ENSDARG00000036816	pou2f2a	16	160	2,71	6,63E-03
ENSDARG00000056184	dvl2	2	25	2,71	2,44E-02
ENSDARG00000078618	inpp5kb	13	224	2,71	3,24E-02
ENSDARG00000067984	gas1b	9	98	2,71	1,23E-02
ENSDARG00000029689	tkta	5	73	2,70	2,16E-02
ENSDARG00000039900	si:ch73-168d20.1	0	25	2,70	7,37E-03
ENSDARG00000012555	ak5	54	700	2,70	2,02E-02
ENSDARG00000003421	igf2bp2a	5	55	2,70	8,93E-03
ENSDARG00000099758	igf2bp2b	3	27	2,70	6,60E-03
ENSDARG00000075126	TMEM8B	38	303	2,70	7,78E-04
ENSDARG00000078754	gra	1	24	2,69	3,36E-02
ENSDARG00000036942	gpd1c	2	38	2,69	1,83E-02
ENSDARG00000101363	ano2	16	215	2,68	3,66E-02
ENSDARG00000027222	adgrg1	2	28	2,68	1,90E-02
ENSDARG00000039352	pald1b	5	61	2,68	3,44E-02
ENSDARG00000013711	zgc:77486	2	26	2,68	3,57E-02
ENSDARG00000045442	cpb1	35	342	2,67	6,34E-03
ENSDARG00000076001	pip5k1ca	4	38	2,67	9,32E-03
ENSDARG00000017121	mafba	3	53	2,67	2,90E-02
ENSDARG00000062346	cacna1ea	18	159	2,66	1,72E-03
ENSDARG00000056909	LO018309.1	308	2848	2,66	1,44E-02
ENSDARG00000001733	gulp1a	0	23	2,66	2,64E-02
ENSDARG00000036105	si:dkeyp-92c9.2	9	97	2,65	1,37E-02
ENSDARG00000057706	si:ch211-137i24.10	4	144	2,65	9,96E-03
ENSDARG00000009553	gng3	43	414	2,64	1,29E-02
ENSDARG00000078094	lmf2a	50	415	2,63	4,88E-03
ENSDARG00000094557	nupr1	22	281	2,63	1,10E-02
ENSDARG00000100582	si:ch211-195b11.3	1	19	2,63	2,60E-02
ENSDARG00000056438	her9	0	23	2,63	1,28E-02
ENSDARG00000044254	anxa3b	4	42	2,63	1,73E-02
ENSDARG00000104497	pcdh1g29	27	241	2,63	7,08E-03
ENSDARG00000036985	plxnb2b	36	298	2,63	5,57E-03
ENSDARG00000007869	ehd3	49	374	2,62	1,42E-03
ENSDARG00000099003	cyp2aa1	7	89	2,62	2,49E-02
ENSDARG00000087981	slc6a11b	99	1249	2,62	3,97E-02
ENSDARG00000030006	zgc:158225	3	33	2,62	1,94E-02
ENSDARG00000077383	anxa11a	731	5148	2,62	2,29E-04
ENSDARG00000089645	si:ch1073-406l10.2	7	59	2,61	3,70E-03
ENSDARG00000007371	slc26a3.1	4	46	2,61	2,76E-02
ENSDARG00000033161	sst1.2	57613	533132	2,61	1,39E-02
ENSDARG00000062289	edaradd	9	78	2,61	9,64E-03
ENSDARG00000101457	cdc42ep4a	74	583	2,61	3,19E-03
ENSDARG00000087704	gfra3	13	141	2,60	1,57E-02
ENSDARG00000103687	sycn.2	0	50	2,60	1,38E-02
ENSDARG00000055226	slc7a7	922	6614	2,59	7,94E-04
ENSDARG00000010601	mtmr10	1	26	2,59	4,66E-02
ENSDARG00000045549	bik	6	46	2,59	2,02E-03
ENSDARG00000022045	map1ab	476	3302	2,59	3,63E-04
ENSDARG00000062350	synm	5	69	2,59	3,43E-02
ENSDARG00000100722	si:ch211-160f23.7	41	354	2,58	8,39E-03
ENSDARG00000078317	si:dkey-175m17.7	3	29	2,58	2,90E-02
ENSDARG00000005989	rgl1	39	310	2,58	4,57E-03
ENSDARG00000008329	asic1a	1	25	2,57	3,47E-02
ENSDARG00000074056	kctd1	0	51	2,57	1,27E-02
ENSDARG00000101171	FO704786.1	6	64	2,57	2,53E-02
ENSDARG00000099677	bambib	86	587	2,57	3,22E-04
ENSDARG00000040907	gcgb	695	8073	2,56	1,36E-02
ENSDARG00000060253	si:ch211-216b21.2	19	165	2,56	1,42E-02
ENSDARG00000102824	pcdh10b	17	133	2,55	7,18E-03
ENSDARG00000018944	hoga1	9	84	2,55	1,62E-02
ENSDARG00000101135	si:dkey-85k7.7	145	1039	2,55	1,99E-03
ENSDARG00000063264	pcdh9	147	1295	2,54	1,66E-02
ENSDARG00000074827	si:ch73-184c24.1	19	150	2,54	4,98E-03
ENSDARG00000076858	TCIM (1 of many)	1	20	2,54	2,50E-02
ENSDARG00000055518	pygma	23	187	2,54	1,27E-02
ENSDARG00000040282	zgc:92590	1	64	2,54	2,62E-02
ENSDARG00000103762	rln1	8	70	2,54	1,84E-02
ENSDARG00000037555	atoh8	24	188	2,53	6,54E-03
ENSDARG00000098834	sox4b	20	153	2,53	3,32E-03
ENSDARG00000032317	tox	368	2278	2,53	1,67E-05
ENSDARG00000099144	igfbp3	5	63	2,52	3,55E-02
ENSDARG00000086070	fgf20b	4	30	2,52	4,88E-03

ENSDARG00000086374	isg15	19	186	2,52	1,31E-02
ENSDARG00000022424	slc26a5	346	2279	2,51	5,42E-04
ENSDARG00000033655	stmn1b	649	4986	2,51	8,14E-03
ENSDARG00000057714	cmah	97	700	2,51	2,42E-03
ENSDARG00000103937	ndrg4	66	491	2,50	3,85E-03
ENSDARG00000069498	phkg1b	7	85	2,50	2,70E-02
ENSDARG00000032765	net1	9	81	2,50	1,12E-02
ENSDARG00000109360	FO704848.1	10	68	2,50	3,62E-03
ENSDARG00000079895	ehbp1l1b	2	29	2,50	2,45E-02
ENSDARG00000060457	pmp22b	286	1984	2,49	3,06E-03
ENSDARG00000004697	rxrgb	2	18	2,49	3,06E-02
ENSDARG00000069186	cyp27a1.2	5	36	2,48	3,75E-03
ENSDARG00000002656	stxbp5a	241	1548	2,48	5,93E-04
ENSDARG00000069139	grik1a	6	59	2,48	4,26E-02
ENSDARG00000073753	tacc1	16	120	2,48	8,57E-03
ENSDARG00000060368	syn1	18	130	2,47	7,33E-03
ENSDARG00000022378	mtmr1b	98	651	2,46	2,36E-03
ENSDARG00000007982	onecut1	2	22	2,45	4,79E-02
ENSDARG00000039034	marcksl1a	2	21	2,44	2,09E-02
ENSDARG00000104367	zgc:194469	21	133	2,43	1,37E-03
ENSDARG00000097639	si:ch73-265h17.4	5	49	2,43	3,89E-02
ENSDARG00000040198	fstl5	2	27	2,43	4,37E-02
ENSDARG00000038788	dnai1.2	12	90	2,43	1,52E-02
ENSDARG00000098113	CABZ01007222.1	5	59	2,43	4,27E-02
ENSDARG00000079064	CABZ01079080.1	4	43	2,42	4,84E-02
ENSDARG00000090292	ralyl	16	137	2,41	3,70E-02
ENSDARG00000068397	tns2b	9	72	2,40	1,75E-02
ENSDARG00000060496	cacna1ha	8	79	2,40	3,72E-02
ENSDARG00000062122	klhl5	15	114	2,39	1,37E-02
ENSDARG00000058179	ackr3b	22	221	2,39	3,75E-02
ENSDARG00000116871	BX284638.1	18	128	2,39	1,12E-02
ENSDARG00000008100	slc7a10a	71	418	2,38	4,43E-04
ENSDARG00000007461	srgap1a	5	37	2,38	1,42E-02
ENSDARG00000058538	alcamb	2480	15162	2,38	1,64E-03
ENSDARG00000089221	si:ch211-195o20.7	29	203	2,38	1,37E-02
ENSDARG00000076135	mmrn2a	3	32	2,37	4,95E-02
ENSDARG00000078468	fap	2	19	2,36	3,69E-02
ENSDARG00000001656	psmb13a	14	86	2,36	1,48E-03
ENSDARG00000074698	sdsl	10	78	2,35	2,51E-02
ENSDARG00000042978	cyp2p6	98	698	2,35	1,77E-02
ENSDARG00000069726	glis3	7	47	2,35	1,90E-02
ENSDARG00000100822	mapk15	75	426	2,34	4,94E-04
ENSDARG00000044039	stx11a	3	30	2,34	2,65E-02
ENSDARG00000092916	mlnl	28	177	2,34	7,48E-03
ENSDARG00000069464	cox7a1	17	117	2,34	1,43E-02
ENSDARG00000031600	rd3	6	45	2,33	2,54E-02
ENSDARG00000098204	CU984600.2	85	511	2,33	2,96E-03
ENSDARG00000045486	pawr	6	35	2,33	4,99E-03
ENSDARG00000075017	myzap	42	243	2,33	1,85E-03
ENSDARG00000013076	bmi1b	53	324	2,33	4,09E-03
ENSDARG00000074265	dzip1l	2	22	2,32	3,57E-02
ENSDARG00000062263	arhgap17b	27	158	2,31	2,02E-03
ENSDARG00000022650	cyp2ad3	10	78	2,31	3,21E-02
ENSDARG00000070423	zgc:153157	10	68	2,31	1,33E-02
ENSDARG00000069708	cdc34a	10	59	2,31	2,29E-03
ENSDARG00000001880	stx3a	59	348	2,30	3,25E-03
ENSDARG00000091531	carm1l	5	34	2,30	1,39E-02
ENSDARG00000103774	limch1b	52	315	2,29	6,75E-03
ENSDARG00000096703	si:dkey-1h6.8	17	120	2,29	3,08E-02
ENSDARG00000070734	dyrk4	7	58	2,29	4,25E-02
ENSDARG00000093549	selenop	4	77	2,28	2,34E-02
ENSDARG00000052400	gpr6	28	176	2,28	1,72E-02
ENSDARG00000024503	c6ast3	0	21	2,28	3,72E-02
ENSDARG00000103602	pcdh1a3	9	52	2,28	6,07E-03
ENSDARG00000018149	slc38a4	24	161	2,28	1,94E-02
ENSDARG00000091006	cobl	122	744	2,28	8,77E-03
ENSDARG00000060637	clstn2	3	30	2,27	4,16E-02
ENSDARG00000045447	slc35g2b	104	609	2,27	7,94E-03
ENSDARG00000103744	hacd1	8	54	2,27	2,64E-02
ENSDARG00000054208	phkg2	214	1165	2,26	1,34E-03
ENSDARG00000074547	si:ch211-240l19.8	2	70	2,26	4,38E-02
ENSDARG00000020693	sesn1	148	890	2,26	9,21E-03
ENSDARG00000079296	gcga	2295	18781	2,24	3,04E-02

ENSDARG00000074316	itga1	59	429	2,23	4,38E-02
ENSDARG00000069681	pcgf6	7	41	2,23	5,74E-03
ENSDARG00000033285	gsto2	29	178	2,23	1,55E-02
ENSDARG00000079977	nhsb	191	1156	2,23	1,89E-02
ENSDARG00000015805	cgnl1	20	117	2,23	1,63E-02
ENSDARG00000003219	bin2a	7	51	2,22	4,04E-02
ENSDARG00000079738	znf219	19	124	2,22	2,27E-02
ENSDARG00000107280	FO744833.1	2	19	2,22	4,53E-02
ENSDARG00000035544	etnppl	4	31	2,22	3,14E-02
ENSDARG00000011373	mknk2a	12	69	2,21	6,66E-03
ENSDARG00000015016	kif26ab	4	29	2,21	4,38E-02
ENSDARG00000038095	socs1a	6	42	2,21	3,14E-02
ENSDARG00000015374	st3gal3a	73	371	2,21	5,03E-04
ENSDARG00000009610	rgs12a	7	52	2,21	4,99E-02
ENSDARG00000075972	csrnp2	66	366	2,20	8,07E-03
ENSDARG00000068193	tmem44	5	29	2,19	1,37E-02
ENSDARG00000088911	cbln17	4	28	2,19	2,66E-02
ENSDARG00000054649	rac1l	3	20	2,18	1,82E-02
ENSDARG00000002196	bach1b	54	269	2,17	6,29E-04
ENSDARG00000079705	si:ch211-152p11.4	86	443	2,17	3,45E-03
ENSDARG00000003818	prkag1	245	1169	2,17	8,38E-05
ENSDARG00000071662	si:rp71-36a1.3	46	241	2,17	4,13E-03
ENSDARG00000024101	adra1d	312	1903	2,16	4,13E-02
ENSDARG00000075201	inpp4b	103	534	2,16	4,08E-03
ENSDARG00000017494	tgfbr1a	6	35	2,16	1,05E-02
ENSDARG00000058302	sh3bgrl	17	95	2,16	1,44E-02
ENSDARG00000018159	ano10b	18	112	2,15	3,47E-02
ENSDARG00000055158	prox1a	66	343	2,15	5,25E-03
ENSDARG00000070213	etnk2	188	951	2,15	2,93E-03
ENSDARG00000053857	ccdc187	23	116	2,14	1,42E-03
ENSDARG00000020133	jdp2b	12	80	2,14	3,75E-02
ENSDARG00000037852	zgc:101663	15	74	2,14	3,32E-03
ENSDARG00000089463	dhx58	16	95	2,13	2,31E-02
ENSDARG00000030844	klf11a	199	1131	2,12	2,53E-02
ENSDARG00000062152	chaf1a	17	94	2,12	1,21E-02
ENSDARG00000069160	fam19a5a	72	335	2,11	6,82E-04
ENSDARG00000032951	nrbp2a	114	648	2,10	4,05E-02
ENSDARG00000009534	wls	202	921	2,10	1,34E-04
ENSDARG00000042876	abracl	5	31	2,10	1,98E-02
ENSDARG00000025218	myo5ab	29	182	2,10	4,72E-02
ENSDARG00000059247	tmem54a	30	146	2,10	2,37E-03
ENSDARG00000034907	paqr7b	24	123	2,10	8,56E-03
ENSDARG00000101865	pcdh1g30	31	160	2,09	1,30E-02
ENSDARG00000046098	ebp	6	35	2,09	2,03E-02
ENSDARG00000101911	si:dkeyp-86d6.2	27	138	2,08	9,09E-03
ENSDARG00000075314	zgc:174906	31	150	2,08	4,26E-03
ENSDARG00000037655	pls3	65	295	2,08	3,82E-04
ENSDARG00000090062	rac3a	27	125	2,07	2,20E-03
ENSDARG00000101090	pappaa	9	133	2,07	3,98E-02
ENSDARG00000054087	irs1	111	545	2,06	9,24E-03
ENSDARG00000074363	TTC9	53	238	2,06	5,75E-04
ENSDARG00000075249	fam171a2a	26	140	2,06	3,87E-02
ENSDARG00000013649	casr	643	2926	2,05	1,29E-03
ENSDARG00000095830	serpinb1l1	7	39	2,05	2,89E-02
ENSDARG00000069171	gramd4a	35	171	2,05	1,03E-02
ENSDARG00000013628	cd164	1435	6435	2,04	7,84E-04
ENSDARG00000055463	lrit3a	20	93	2,04	1,96E-03
ENSDARG00000043406	slc8a1b	54	280	2,04	3,18E-02
ENSDARG00000019845	pdlim1	19	93	2,04	1,25E-02
ENSDARG00000001463	tdh2	38	175	2,04	2,52E-03
ENSDARG00000079659	wdr53	8	40	2,03	7,47E-03
ENSDARG00000006283	waslb	63	279	2,02	1,43E-03
ENSDARG00000055751	fosb	416	2023	2,02	1,69E-02
ENSDARG00000093714	artnb	70	320	2,02	4,47E-03
ENSDARG00000038222	tyw3	7	38	2,01	3,39E-02
ENSDARG00000035563	znf703	7	34	2,01	1,90E-02
ENSDARG00000095295	si:rp71-36a1.1	38	204	2,00	4,28E-02
ENSDARG00000095719	si:dkey-222h21.2	4	18	2,00	4,20E-02
ENSDARG00000003486	ppp1caa	1601	7003	2,00	1,48E-03
ENSDARG00000086856	stk35	72	339	2,00	1,49E-02
ENSDARG00000094210	zgc:109934	18	89	1,99	1,53E-02
ENSDARG00000071487	zgc:152948	5	25	1,98	3,90E-02
ENSDARG00000074812	fhdc1	31	144	1,98	1,34E-02

ENSDARG00000076716	palb2	4	20	1,98	3,02E-02
ENSDARG00000052604	cpeb2	28	139	1,98	3,77E-02
ENSDARG00000069595	si:ch211-214c7.4	35	158	1,98	7,96E-03
ENSDARG00000008494	myl6	159	669	1,98	5,55E-04
ENSDARG00000052690	arrdc3a	521	2381	1,98	1,14E-02
ENSDARG00000044319	fstl4	71	323	1,98	8,75E-03
ENSDARG00000002013	grb10a	77	372	1,97	2,47E-02
ENSDARG00000103551	rnf213b	37	176	1,97	2,25E-02
ENSDARG00000020623	baxa	50	204	1,95	1,86E-04
ENSDARG00000034168	serpini1	332	1433	1,95	5,35E-03
ENSDARG00000013082	uap1l1	16	78	1,95	3,53E-02
ENSDARG00000005897	dera	183	738	1,95	1,07E-04
ENSDARG00000019420	etnk1	490	1995	1,95	3,23E-04
ENSDARG00000016866	fam102ab	85	376	1,94	1,30E-02
ENSDARG00000043710	parvaa	17	76	1,94	6,88E-03
ENSDARG00000054300	dhrs1	769	3075	1,93	1,94E-04
ENSDARG00000079805	tagln3a	22	95	1,93	1,50E-02
ENSDARG00000031814	dhrs13b	12	58	1,93	3,80E-02
ENSDARG00000078536	si:ch211-158d24.2	50	214	1,92	9,71E-03
ENSDARG00000005780	npy8br	333	1381	1,92	3,19E-03
ENSDARG00000039852	ftr93	18	85	1,92	3,19E-02
ENSDARG00000095019	lmo2	33	152	1,92	2,98E-02
ENSDARG00000003835	stom	145	620	1,91	9,99E-03
ENSDARG00000089177	cyp46a1.3	10	45	1,90	2,34E-02
ENSDARG00000039422	fuom	15	64	1,89	1,05E-02
ENSDARG00000079397	cerkl	40	156	1,88	1,40E-03
ENSDARG00000068629	cd151l	88	346	1,87	9,26E-04
ENSDARG00000012600	si:dkey-97a13.6	25	106	1,87	2,53E-02
ENSDARG00000099379	ppp4r1l	138	546	1,87	2,73E-03
ENSDARG00000054588	cox6a2	159	673	1,87	1,46E-02
ENSDARG00000031348	mc5ra	355	1473	1,87	1,30E-02
ENSDARG00000069430	tp53i11a	42	184	1,87	3,48E-02
ENSDARG00000075163	cxcl20	92	376	1,87	9,32E-03
ENSDARG00000043856	amd1	3173	12174	1,87	4,28E-04
ENSDARG00000068732	spry4	636	2640	1,86	1,34E-02
ENSDARG00000093760	si:ch211-197h24.9	26	114	1,85	4,74E-02
ENSDARG00000055831	si:dkey-182g1.6	13	60	1,85	3,36E-02
ENSDARG00000101534	rab3db	450	1729	1,85	1,24E-03
ENSDARG00000069150	rit1	33	143	1,85	3,71E-02
ENSDARG00000009881	ier5	455	1950	1,85	3,22E-02
ENSDARG00000021987	plecb	265	1054	1,85	6,95E-03
ENSDARG00000077791	brsk2b	38	163	1,84	3,41E-02
ENSDARG00000075158	igdcc3	103	427	1,84	2,03E-02
ENSDARG00000086490	si:dkey-92f12.2	38	151	1,84	1,10E-02
ENSDARG00000038964	traf4b	5	20	1,84	4,94E-02
ENSDARG00000034181	esr2b	52	217	1,83	2,38E-02
ENSDARG00000074163	zgc:162879	8	32	1,83	2,49E-02
ENSDARG00000054454	epha4a	434	1693	1,83	5,81E-03
ENSDARG00000008310	ip6k2a	359	1418	1,83	9,91E-03
ENSDARG00000030723	cldn11b	5	22	1,82	4,94E-02
ENSDARG00000018524	midn	2470	9492	1,82	4,14E-03
ENSDARG00000021869	rcan2	82	321	1,82	8,19E-03
ENSDARG00000015709	hsd17b12a	139	520	1,82	1,11E-03
ENSDARG00000087413	bean1	151	579	1,81	4,68E-03
ENSDARG00000030803	zgc:110006	19	78	1,81	3,74E-02
ENSDARG00000045465	lancl2	111	421	1,81	3,87E-03
ENSDARG00000040815	tsku	25	104	1,80	4,71E-02
ENSDARG00000034262	pld2	84	318	1,80	3,50E-03
ENSDARG00000061817	kif1aa	109	422	1,80	8,84E-03
ENSDARG00000076752	tmem67	29	115	1,80	1,94E-02
ENSDARG00000039512	ap1m3	13	52	1,80	2,14E-02
ENSDARG00000026178	ube3d	11	45	1,80	2,91E-02
ENSDARG00000079124	si:dkeyp-94h10.5	25	99	1,80	1,33E-02
ENSDARG00000004115	mgat4b	117	442	1,80	5,32E-03
ENSDARG00000038655	ajap1	33	135	1,80	3,22E-02
ENSDARG00000036179	myo1ea	192	719	1,80	3,94E-03
ENSDARG00000012972	cfl1l	23	97	1,79	3,52E-02
ENSDARG00000031647	stat2	111	427	1,79	9,49E-03
ENSDARG00000006314	itgav	70	257	1,79	2,00E-03
ENSDARG00000004937	skp2	4	18	1,79	1,55E-02
ENSDARG00000030472	slc16a5a	35	138	1,78	2,37E-02
ENSDARG00000043210	nfic	75	295	1,78	1,94E-02
ENSDARG00000053205	ppfia4	220	832	1,78	1,10E-02

ENSDARG00000023958	rnf181	38	138	1,77	1,22E-03
ENSDARG00000008034	skib	90	338	1,77	1,23E-02
ENSDARG00000069101	napbb	648	2456	1,77	1,35E-02
ENSDARG00000062702	ankmy1	25	98	1,76	1,45E-02
ENSDARG00000101928	gorasp1b	9	36	1,76	2,85E-02
ENSDARG00000036493	si:ch73-174h16.4	18	69	1,76	1,10E-02
ENSDARG00000055092	pora	88	319	1,76	2,39E-03
ENSDARG00000091602	si:dkey-163f14.6	48	187	1,76	3,22E-02
ENSDARG00000009336	aif1l	96	341	1,76	6,34E-04
ENSDARG00000037884	rab30	164	591	1,75	3,36E-03
ENSDARG00000017489	zgc:123068	242	901	1,75	1,10E-02
ENSDARG00000056519	si:dkey-280e21.3	160	595	1,75	1,30E-02
ENSDARG00000015199	cblb	669	2487	1,74	1,46E-02
ENSDARG00000055099	fam184b	154	562	1,74	8,33E-03
ENSDARG00000074305	si:ch73-257c13.2	28	101	1,74	6,61E-03
ENSDARG00000032761	pde4d	41	153	1,74	1,77E-02
ENSDARG00000023498	gmppab	53	194	1,74	7,58E-03
ENSDARG00000037030	casz1	45	175	1,74	3,50E-02
ENSDARG00000041925	cryba2b	7	29	1,73	4,94E-02
ENSDARG00000088862	stxbp6	466	1721	1,73	1,84E-02
ENSDARG00000089066	nhsl2	293	1048	1,73	6,21E-03
ENSDARG00000094791	si:dkey-193n17.9	10	38	1,72	2,91E-02
ENSDARG00000002826	cep57l1	22	87	1,72	3,90E-02
ENSDARG00000078404	cdh26.1	21	79	1,72	3,26E-02
ENSDARG00000014871	syngr3a	28	102	1,72	7,58E-03
ENSDARG00000061579	myo1cb	125	450	1,72	1,05E-02
ENSDARG00000060309	srgap3	196	688	1,72	3,38E-03
ENSDARG00000043131	BX664625.1	10	40	1,71	4,84E-02
ENSDARG00000042122	acot11b	83	316	1,71	4,82E-02
ENSDARG00000051749	tigara	20	74	1,71	2,04E-02
ENSDARG00000063686	atp23	8	33	1,70	4,14E-02
ENSDARG00000087780	tiam2a	80	278	1,70	5,27E-03
ENSDARG00000011027	fgfr1a	554	1948	1,70	8,84E-03
ENSDARG00000021664	fzd3a	57	210	1,69	3,45E-02
ENSDARG00000068586	CDHR2	297	1017	1,69	2,52E-03
ENSDARG00000043416	tnfaip2a	311	1061	1,69	1,85E-03
ENSDARG00000079586	gabrb2	103	374	1,69	2,94E-02
ENSDARG00000028164	cst14a.1	10	36	1,68	4,31E-02
ENSDARG00000079236	si:dkey-253d23.11	5	18	1,67	2,15E-02
ENSDARG00000060338	trmt12	21	76	1,67	1,75E-02
ENSDARG00000021113	ptmaa	222	758	1,67	4,86E-03
ENSDARG00000033599	zgc:100906	143	485	1,67	3,89E-03
ENSDARG00000093687	si:dkey-193c22.1	106	359	1,67	3,43E-03
ENSDARG00000075858	fam155a	79	273	1,67	1,35E-02
ENSDARG00000005590	mtf2	6	24	1,66	3,77E-02
ENSDARG00000004402	elovl6	78	257	1,66	6,63E-04
ENSDARG00000070498	phyhiplb	138	510	1,66	4,42E-02
ENSDARG00000103848	apba1a	48	166	1,66	9,40E-03
ENSDARG00000056888	dnah5l	88	318	1,66	4,25E-02
ENSDARG00000093412	sertad2a	19	65	1,66	1,15E-02
ENSDARG00000086699	smim15	40	134	1,65	2,05E-03
ENSDARG00000016427	fignl1	22	76	1,65	1,42E-02
ENSDARG00000035918	bzw2	86	290	1,65	1,22E-02
ENSDARG00000074030	myt1a	156	511	1,65	1,51E-03
ENSDARG00000045661	irf7	59	206	1,64	3,52E-02
ENSDARG00000012040	rnf19a	348	1152	1,64	4,63E-03
ENSDARG00000007657	ccnh	37	126	1,64	8,76E-03
ENSDARG00000007975	fth1b	26	90	1,64	1,63E-02
ENSDARG00000104409	tnk2b	71	246	1,63	3,08E-02
ENSDARG00000000476	pms1	11	38	1,63	2,53E-02
ENSDARG00000104478	cap2	88	295	1,63	1,14E-02
ENSDARG00000035957	gmnn	27	94	1,63	2,73E-02
ENSDARG00000093864	wee1	26	91	1,63	3,17E-02
ENSDARG00000060215	cep85	37	120	1,62	7,13E-03
ENSDARG00000021239	apaf1	113	365	1,62	2,88E-03
ENSDARG00000093117	si:dkey-266m15.7	16	55	1,62	2,98E-02
ENSDARG00000023858	ccdc174	76	242	1,61	8,20E-04
ENSDARG00000078077	dph2	12	43	1,61	3,55E-02
ENSDARG00000069940	ppap2d	303	964	1,60	2,08E-03
ENSDARG00000076246	prune	27	90	1,59	2,15E-02
ENSDARG00000090882	si:rp71-36a1.5	69	240	1,59	4,95E-02
ENSDARG00000079884	trim107	26	86	1,59	1,60E-02
ENSDARG00000005675	sec61a1l	347	1089	1,59	1,66E-03

ENSDARG00000070261	crlf3	44	146	1,59	3,48E-02
ENSDARG00000009242	fev	832	2637	1,58	4,86E-03
ENSDARG00000060306	tfcp2	633	1978	1,58	1,52E-03
ENSDARG00000032188	lrrc8aa	76	244	1,58	1,12E-02
ENSDARG00000088967	wnk4b	956	3120	1,58	2,19E-02
ENSDARG00000112454	FO744833.2	19	61	1,58	1,44E-02
ENSDARG00000044001	lgals3b	22	73	1,57	1,94E-02
ENSDARG00000021607	negr1	68	216	1,57	1,11E-02
ENSDARG00000034001	tango6	13	43	1,57	1,77E-02
ENSDARG00000014017	rrm1	23	72	1,56	1,53E-02
ENSDARG00000013000	ppfia2	33	108	1,56	3,70E-02
ENSDARG00000088663	lman2lb	84	264	1,56	6,51E-03
ENSDARG00000034771	rab13	39	126	1,56	3,39E-02
ENSDARG00000003193	rassf7b	28	92	1,56	2,17E-02
ENSDARG00000076221	zgc:198419	148	473	1,56	1,86E-02
ENSDARG00000099106	afmid	12	39	1,55	4,18E-02
ENSDARG00000106873	LO018188.1	163	523	1,55	2,50E-02
ENSDARG00000020212	slc1a1	16	53	1,55	4,91E-02
ENSDARG00000114852	CR974456.1	65	212	1,55	3,98E-02
ENSDARG00000026090	adprm	38	121	1,54	3,09E-02
ENSDARG00000057625	dusp14	46	138	1,53	1,26E-03
ENSDARG00000079578	rbpms2b	31	101	1,53	4,33E-02
ENSDARG00000101348	plppr5b	75	233	1,53	1,64E-02
ENSDARG00000006754	mllt3	42	132	1,52	3,60E-02
ENSDARG00000062083	dph7	16	52	1,52	4,16E-02
ENSDARG00000059646	nt5dc2	298	939	1,52	3,43E-02
ENSDARG00000087402	TPM1 (1 of many)	153	475	1,52	2,39E-02
ENSDARG00000043821	klf7b	323	982	1,52	1,16E-02
ENSDARG00000070539	arf3a	81	246	1,51	9,08E-03
ENSDARG00000094343	si:dkey-20i20.8	13	41	1,51	2,04E-02
ENSDARG00000013950	pdf	10	33	1,51	3,71E-02
ENSDARG00000027887	zfyve21	187	547	1,50	6,39E-04
ENSDARG00000062054	cpt1ab	277	849	1,50	2,37E-02
ENSDARG00000102061	b3galt2	114	336	1,49	6,17E-03
ENSDARG00000089877	si:dkeyp-75h12.5	397	1162	1,49	2,44E-03
ENSDARG00000093003	acy3.1	9	27	1,49	2,41E-02
ENSDARG00000062790	CU639469.1	535	1549	1,48	1,54E-03
ENSDARG00000063646	lnpa	45	132	1,48	6,34E-03
ENSDARG00000090986	si:dkeyp-115e12.6	63	187	1,48	1,65E-02
ENSDARG00000060796	slc20a2	210	615	1,47	1,10E-02
ENSDARG00000058038	eml3	50	149	1,47	2,26E-02
ENSDARG00000090872	si:dkey-276j7.1	181	526	1,47	5,74E-03
ENSDARG00000076362	tmem260	18	54	1,47	3,36E-02
ENSDARG00000004325	casp9	69	199	1,47	5,78E-03
ENSDARG00000004262	cyp46a1.2	25	75	1,46	1,15E-02
ENSDARG00000007654	nsfa	474	1347	1,46	1,74E-03
ENSDARG00000003989	crhr1	58	169	1,45	2,17E-02
ENSDARG00000092228	si:dkey-27p18.3	58	168	1,45	1,28E-02
ENSDARG00000040505	yif1b	21	62	1,45	8,03E-03
ENSDARG00000021143	rtn1b	871	2531	1,45	1,90E-02
ENSDARG00000045087	cdk5r1b	87	258	1,45	4,94E-02
ENSDARG00000086808	ddhd1a	55	163	1,45	4,31E-02
ENSDARG00000101979	eps8l3a	47	136	1,45	1,49E-02
ENSDARG00000099336	mvda	20	59	1,45	2,80E-02
ENSDARG00000045485	rassf8b	20	58	1,44	4,56E-02
ENSDARG00000079438	pkp3b	72	206	1,44	1,24E-02
ENSDARG00000063711	trim3a	93	260	1,44	1,15E-03
ENSDARG00000103542	vegfaa	479	1334	1,44	1,04E-03
ENSDARG00000021209	kctd9a	174	497	1,43	1,57E-02
ENSDARG00000088072	usp43b	59	171	1,43	3,05E-02
ENSDARG00000002949	ppp6c	557	1542	1,43	1,56E-03
ENSDARG00000035578	hs3st1l2	96	270	1,42	7,27E-03
ENSDARG00000008487	dmd	116	325	1,42	1,03E-02
ENSDARG00000056152	fam3c	238	662	1,42	3,64E-03
ENSDARG00000014704	pgm2l1	167	462	1,42	2,23E-03
ENSDARG00000063169	ubn1	12	35	1,42	9,28E-03
ENSDARG00000054442	cbx1b	80	222	1,42	4,24E-03
ENSDARG00000061691	qpctla	65	177	1,41	2,29E-04
ENSDARG00000006567	dus4l	28	77	1,40	7,37E-03
ENSDARG00000061713	tpd52	289	792	1,40	3,29E-03
ENSDARG00000094345	si:dkey-261l7.2	34	95	1,40	3,01E-02
ENSDARG00000103720	zgc:162730	2100	5729	1,40	3,33E-03
ENSDARG00000030716	ing4	42	119	1,40	3,11E-02

ENSDARG00000033516	efr3ba	843	2353	1,40	2,23E-02
ENSDARG00000019503	zgc:103759	11	31	1,39	3,42E-02
ENSDARG00000004840	rassf1	190	511	1,39	1,11E-03
ENSDARG00000019335	hes6	87	235	1,38	6,81E-03
ENSDARG00000058219	tubd1	50	137	1,38	2,31E-02
ENSDARG00000037932	lin7b	117	318	1,38	7,37E-03
ENSDARG00000101367	si:ch73-193i22.1	121	340	1,38	3,75E-02
ENSDARG00000109820	NPAS3	44	122	1,38	2,50E-02
ENSDARG00000035433	arpc5lb	74	203	1,38	8,03E-03
ENSDARG00000018976	sptlc2a	214	575	1,37	3,96E-03
ENSDARG00000025855	camk2n1a	871	2379	1,37	2,06E-02
ENSDARG00000063446	iba57	84	229	1,37	2,57E-02
ENSDARG00000000837	snx9a	180	497	1,37	3,57E-02
ENSDARG00000091555	ostf1	84	229	1,37	2,61E-02
ENSDARG00000030479	hmgb1b	700	1848	1,36	1,27E-03
ENSDARG00000063075	soga1	203	546	1,35	2,59E-02
ENSDARG00000054864	aplp2	1509	3974	1,33	1,47E-02
ENSDARG00000104015	fgfr1bl	41	110	1,33	4,95E-02
ENSDARG00000033413	acot18	106	276	1,33	6,67E-03
ENSDARG00000045131	id4	210	542	1,33	3,33E-03
ENSDARG00000036102	ctdsp2	93	247	1,33	2,98E-02
ENSDARG00000035870	laptm4b	562	1443	1,32	2,41E-03
ENSDARG00000005772	tsr2	23	62	1,32	3,40E-02
ENSDARG00000104767	CR381531.2	66	174	1,32	2,83E-02
ENSDARG00000035329	capns1a	204	523	1,31	3,75E-03
ENSDARG00000055477	pelo	124	327	1,31	3,42E-02
ENSDARG00000020841	rbm7	21	54	1,30	2,08E-02
ENSDARG00000033539	paics	88	224	1,30	5,16E-03
ENSDARG00000053452	pop5	21	54	1,30	2,14E-02
ENSDARG00000018966	cyb5r1	272	689	1,30	2,68E-03
ENSDARG00000061314	ngrn	38	100	1,30	4,33E-02
ENSDARG00000068787	slc6a17	276	705	1,30	1,14E-02
ENSDARG00000030914	tmem120a	33	87	1,30	4,02E-02
ENSDARG00000061800	si:dkey-208k22.6	87	222	1,30	5,86E-03
ENSDARG00000087369	cep162	33	85	1,30	3,14E-02
ENSDARG00000037260	toporsa	40	103	1,30	1,32E-02
ENSDARG00000019236	gsr	90	229	1,30	6,90E-03
ENSDARG00000000151	thraa	84	213	1,29	6,27E-03
ENSDARG00000043548	atg4da	31	81	1,29	2,08E-02
ENSDARG00000029865	rassf2a	122	311	1,29	1,30E-02
ENSDARG00000060515	zdhhc9	120	307	1,29	1,76E-02
ENSDARG00000069279	elovl7a	25	65	1,29	1,89E-02
ENSDARG00000021309	rhoca	132	333	1,29	8,06E-03
ENSDARG00000102846	trmt13	16	42	1,28	2,60E-02
ENSDARG00000099511	CABZ01034698.2	32	82	1,28	3,70E-02
ENSDARG00000071594	TIMM21	42	107	1,28	3,72E-02
ENSDARG00000039270	vti1b	61	154	1,28	2,16E-02
ENSDARG00000088972	si:dkey-238d18.4	137	349	1,28	3,23E-02
ENSDARG00000092889	zgc:194246	29	73	1,27	7,38E-03
ENSDARG00000074628	si:dkey-10o6.2	61	157	1,27	4,74E-02
ENSDARG00000040971	zgc:92606	404	1004	1,26	8,60E-03
ENSDARG00000099226	PPP1CC	222	564	1,26	4,82E-02
ENSDARG00000021753	ccdc25	222	547	1,26	3,62E-03
ENSDARG00000092364	si:ch211-218c6.8	40	99	1,26	2,37E-02
ENSDARG00000057114	hpf1	123	301	1,26	2,48E-03
ENSDARG00000114038	ska2	74	183	1,25	7,87E-03
ENSDARG00000060445	elp6	41	103	1,24	4,46E-02
ENSDARG00000042732	tmem189	79	194	1,24	1,49E-02
ENSDARG00000041665	mkrn1	102	252	1,24	3,24E-02
ENSDARG00000040300	gtpbp10	42	105	1,24	4,31E-02
ENSDARG00000101936	tmem106ba	260	626	1,24	1,45E-03
ENSDARG00000045955	prlrb	48	120	1,24	4,20E-02
ENSDARG00000070266	spock3	937	2332	1,24	4,99E-02
ENSDARG00000053583	cers6	471	1141	1,23	7,94E-03
ENSDARG00000040123	zfpm2a	98	237	1,23	1,44E-02
ENSDARG00000011605	dennd6b	88	213	1,23	1,89E-02
ENSDARG00000075491	si:ch211-26b3.4	69	166	1,22	2,19E-02
ENSDARG00000001889	tuba1a	54	133	1,22	4,33E-02
ENSDARG00000098780	nsmce1	31	75	1,22	3,08E-02
ENSDARG00000011555	spag7	83	197	1,22	2,83E-03
ENSDARG00000046141	zgc:162025	64	155	1,22	2,43E-02
ENSDARG00000019117	parvb	71	170	1,22	7,94E-03
ENSDARG00000094265	si:dkey-183n20.15	50	122	1,21	2,91E-02

ENSDARG00000015472	gpc4	560	1343	1,21	2,24E-02
ENSDARG00000005651	hrasb	59	141	1,21	4,03E-03
ENSDARG00000000760	znf511	44	105	1,20	2,11E-02
ENSDARG00000057021	vps36	63	147	1,20	6,52E-04
ENSDARG00000058225	arpc4l	263	619	1,20	4,02E-03
ENSDARG00000020944	ezra	79	190	1,20	2,81E-02
ENSDARG00000090503	oma1	17	41	1,20	2,61E-02
ENSDARG00000101735	chn1	42	102	1,20	4,20E-02
ENSDARG00000033987	rsrc1	30	72	1,20	4,01E-02
ENSDARG00000031261	sap30bp	190	453	1,20	2,33E-02
ENSDARG00000091592	peli1a	223	529	1,19	2,71E-02
ENSDARG00000076532	si:ch211-222l21.1	575	1352	1,18	1,87E-02
ENSDARG00000012222	nup35	54	126	1,18	5,34E-03
ENSDARG00000104709	sprtn	24	58	1,18	4,96E-02
ENSDARG00000075564	fam13a	395	924	1,18	1,73E-02
ENSDARG00000041600	wdr83	47	109	1,18	1,46E-02
ENSDARG00000101996	cxxc1a	88	205	1,18	9,91E-03
ENSDARG00000028327	gmfb	139	321	1,18	1,77E-03
ENSDARG00000024818	rilpl2	91	211	1,17	1,40E-02
ENSDARG00000095538	senp3a	22	52	1,17	3,05E-02
ENSDARG00000035398	enc1	345	797	1,17	1,14E-02
ENSDARG00000014085	atp6v1e1a	88	203	1,17	8,69E-03
ENSDARG00000089720	CABZ01077218.1	59	136	1,17	1,87E-02
ENSDARG00000028088	galk1	70	161	1,17	1,38E-02
ENSDARG00000014333	ormdl1	225	519	1,17	1,32E-02
ENSDARG00000017261	gdpd1	193	445	1,16	1,54E-02
ENSDARG00000099439	serp1	690	1599	1,16	2,52E-02
ENSDARG00000059483	tead1b	281	647	1,16	1,56E-02
ENSDARG00000002994	itpkca	36	83	1,16	4,97E-02
ENSDARG00000062056	elmod1	127	292	1,16	1,38E-02
ENSDARG00000075129	mrps11	65	151	1,16	2,84E-02
ENSDARG00000045482	stk38l	108	248	1,16	1,13E-02
ENSDARG00000041237	med18	25	59	1,16	2,06E-02
ENSDARG00000034885	bet1	108	252	1,16	4,56E-02
ENSDARG00000001303	psmb8a	160	368	1,15	1,73E-02
ENSDARG00000041155	morf4l1	296	672	1,15	5,81E-03
ENSDARG00000011661	twf1b	92	207	1,15	2,04E-03
ENSDARG00000036147	ano5b	84	193	1,14	3,89E-02
ENSDARG00000044760	gnaia	232	524	1,14	6,15E-03
ENSDARG00000099510	cnn3a	459	1036	1,14	7,26E-03
ENSDARG00000002339	sgf29	53	122	1,14	4,15E-02
ENSDARG00000099799	si:dkey-11f4.16	69	158	1,14	4,70E-02
ENSDARG00000070061	gfer	63	145	1,14	4,27E-02
ENSDARG00000010052	ndrg3b	1285	2910	1,13	2,73E-02
ENSDARG00000062489	march8	990	2211	1,13	9,87E-03
ENSDARG00000041071	jagn1a	150	336	1,13	1,08E-02
ENSDARG00000070833	lin52	86	193	1,12	2,15E-02
ENSDARG00000079584	zgc:194930	231	509	1,12	8,17E-04
ENSDARG00000045843	apex1	47	105	1,12	1,72E-02
ENSDARG00000004023	isl1	2468	5493	1,12	1,20E-02
ENSDARG00000007025	ttc9c	19	44	1,12	4,16E-02
ENSDARG00000027986	slc35d1b	94	209	1,11	1,90E-02
ENSDARG00000044923	ube2w	249	550	1,11	1,54E-02
ENSDARG00000101446	PCP4L1	102	228	1,10	3,50E-02
ENSDARG00000029252	ssb	58	127	1,10	2,04E-02
ENSDARG00000018529	lipf	111	243	1,10	1,82E-02
ENSDARG00000070986	sergef	42	93	1,10	4,31E-02
ENSDARG00000029107	ube2d1a	62	137	1,10	4,51E-02
ENSDARG00000101063	CABZ01058650.1	78	173	1,10	3,32E-02
ENSDARG00000059906	sdc4	973	2144	1,10	3,02E-02
ENSDARG00000030871	siah1	109	239	1,09	1,75E-02
ENSDARG00000092746	si:ch211-150o23.2	105	229	1,09	1,92E-02
ENSDARG00000020777	cep85l	51	111	1,09	1,90E-02
ENSDARG00000098646	mthfd2	110	239	1,09	1,81E-02
ENSDARG00000017874	dnajc3b	141	305	1,09	7,27E-03
ENSDARG00000054540	imp4	31	69	1,08	4,29E-02
ENSDARG00000011498	si:ch211-212o1.2	152	327	1,08	1,30E-02
ENSDARG00000044642	sc5d	63	136	1,08	1,43E-02
ENSDARG00000035146	polr2j	57	125	1,08	2,91E-02
ENSDARG00000079994	AKAP13	583	1258	1,08	1,62E-02
ENSDARG00000045540	ppp6r2a	580	1242	1,07	7,47E-03
ENSDARG00000021582	dynlrb1	233	499	1,07	6,80E-03
ENSDARG00000010280	clasp1a	183	393	1,07	1,76E-02

ENSDARG00000076919	igdcc4	352	755	1,07	1,61E-02
ENSDARG00000062476	snapc5	112	242	1,07	4,25E-02
ENSDARG00000104637	fam219b	155	329	1,07	4,37E-03
ENSDARG00000056396	dusp22a	48	105	1,06	4,82E-02
ENSDARG00000057884	zgc:114174	54	117	1,06	2,74E-02
ENSDARG00000011488	sirt2	240	508	1,06	3,29E-03
ENSDARG00000003531	tcea1	174	372	1,06	1,14E-02
ENSDARG00000002549	eif3eb	77	164	1,06	3,24E-03
ENSDARG00000028295	mkrn4	73	155	1,05	3,37E-02
ENSDARG00000102998	taf6	147	310	1,05	6,56E-03
ENSDARG00000006878	phf21aa	188	397	1,05	1,01E-02
ENSDARG00000104030	taf12	481	1017	1,05	1,23E-02
ENSDARG00000104894	si:ch73-138e16.2	52	112	1,05	3,17E-02
ENSDARG00000077804	hgsnat	53	112	1,05	8,14E-03
ENSDARG00000069085	ints2	159	338	1,05	3,47E-02
ENSDARG00000092467	si:ch73-46j18.5	680	1452	1,05	4,95E-02
ENSDARG00000070600	DYRK3	335	709	1,05	2,08E-02
ENSDARG00000099749	ube2d2l	119	254	1,05	4,99E-02
ENSDARG00000012866	picalma	314	669	1,05	4,83E-02
ENSDARG00000003749	polb	85	180	1,04	2,38E-02
ENSDARG00000033738	zgc:153867	686	1432	1,04	4,84E-03
ENSDARG00000077253	alkbh6	40	85	1,04	4,30E-02
ENSDARG00000053070	gosr2	300	632	1,04	2,80E-02
ENSDARG00000055912	nipa2	151	321	1,04	4,35E-02
ENSDARG00000093240	GMEB2	230	485	1,04	4,60E-02
ENSDARG00000037009	banf1	155	326	1,03	2,12E-02
ENSDARG00000006719	specc1la	159	330	1,03	2,84E-03
ENSDARG00000069910	gtf2f2a	247	515	1,03	1,22E-02
ENSDARG00000069494	tmem223	28	59	1,03	4,33E-02
ENSDARG00000078315	zgc:110063	42	88	1,03	4,65E-02
ENSDARG00000070553	rnmt	117	244	1,03	1,44E-02
ENSDARG00000040584	eef1akmt1	71	149	1,03	4,34E-02
ENSDARG00000019530	hdgfl2	79	166	1,03	2,55E-02
ENSDARG00000014462	rab3c	146	302	1,03	7,16E-03
ENSDARG00000054858	tp53bp2b	105	218	1,02	1,45E-02
ENSDARG00000020834	si:dkey-230p4.1	84	175	1,02	3,08E-02
ENSDARG00000063572	perp	161	334	1,02	1,37E-02
ENSDARG00000095086	cfdp1	46	97	1,02	4,75E-02
ENSDARG00000039136	cox16	129	267	1,02	2,56E-02
ENSDARG00000045025	ift52	57	118	1,01	1,93E-02
ENSDARG00000068246	plcb3	754	1554	1,01	2,12E-02
ENSDARG00000076689	gramd1a	224	460	1,01	1,49E-02
ENSDARG00000033140	desi1a	190	395	1,01	4,78E-02
ENSDARG00000011597	atxn2l	435	212	-1,00	3,69E-02
ENSDARG00000045909	dynlt1	370	180	-1,00	2,28E-02
ENSDARG00000059925	usp24	493	242	-1,00	7,59E-03
ENSDARG00000016875	gys1	395	193	-1,01	2,08E-02
ENSDARG00000115547	CABZ01026681.1	207	101	-1,01	9,05E-03
ENSDARG00000006094	igf2r	595	288	-1,01	4,22E-02
ENSDARG00000055530	sertad2b	346	168	-1,01	2,75E-02
ENSDARG00000006019	tktb	456	221	-1,01	1,65E-02
ENSDARG00000053136	b2m	10309	5025	-1,02	4,70E-03
ENSDARG00000074903	itcha	357	172	-1,02	2,98E-02
ENSDARG00000022712	stat3	1051	506	-1,02	2,68E-02
ENSDARG00000033537	p4ha1a	235	112	-1,02	4,54E-02
ENSDARG00000069013	prdx4	4543	2185	-1,02	2,52E-02
ENSDARG00000040965	zgc:101731	1056	509	-1,02	1,81E-02
ENSDARG00000068894	nrip1b	873	424	-1,02	3,29E-03
ENSDARG00000032369	btbd6b	648	309	-1,02	4,76E-02
ENSDARG00000036687	bcl9	347	167	-1,03	1,94E-02
ENSDARG00000063457	wash1	87	41	-1,03	4,45E-02
ENSDARG00000100717	socs5a	119	57	-1,03	4,78E-02
ENSDARG00000100222	utrn	418	200	-1,03	3,43E-02
ENSDARG00000018997	cplx2l	1890	910	-1,03	1,32E-02
ENSDARG00000074229	prr12a	1272	607	-1,03	3,59E-02
ENSDARG00000062744	scn1lab	625	300	-1,03	2,09E-02
ENSDARG00000104435	si:ch73-217b7.1	372	178	-1,03	2,40E-02
ENSDARG00000075819	arhgef2	225	108	-1,03	1,59E-02
ENSDARG00000054930	tpra1	239	114	-1,03	1,55E-02
ENSDARG00000007727	rgl2	445	211	-1,03	3,91E-02
ENSDARG00000019702	aldocb	9526	4515	-1,03	4,45E-02
ENSDARG00000056156	npdc1b	459	218	-1,03	3,03E-02
ENSDARG00000068981	glceb	343	164	-1,04	1,03E-02

ENSDARG00000086678	ascc3	723	345	-1,04	1,05E-02
ENSDARG00000074696	zgc:194621	350	167	-1,04	1,19E-02
ENSDARG00000003789	dennd5b	304	145	-1,04	9,77E-03
ENSDARG00000056092	si:dkey-12h9.6	213	101	-1,04	4,74E-02
ENSDARG00000078222	aatka	932	445	-1,05	6,27E-03
ENSDARG00000062055	rnf38	366	172	-1,05	3,64E-02
ENSDARG00000076618	tm2d3	240	112	-1,05	4,35E-02
ENSDARG00000010437	fam46c	3541	1682	-1,05	9,11E-03
ENSDARG00000039319	vps37b	202	95	-1,05	1,06E-02
ENSDARG00000035285	ncor1	3094	1473	-1,05	3,73E-03
ENSDARG00000005867	gon4l	395	187	-1,05	7,57E-03
ENSDARG00000077901	herc1	637	301	-1,05	1,18E-02
ENSDARG00000062477	kiaa1549la	1066	500	-1,06	2,38E-02
ENSDARG00000075281	tbc1d30	606	284	-1,06	1,92E-02
ENSDARG00000062531	mapk8ip3	744	353	-1,06	2,95E-03
ENSDARG00000042551	mboat2b	446	210	-1,06	4,30E-03
ENSDARG00000055052	map2	1492	703	-1,06	3,55E-03
ENSDARG00000076796	dlg3	185	87	-1,06	1,15E-02
ENSDARG00000056331	ahcyl1	619	292	-1,07	1,10E-03
ENSDARG00000078014	pacsin2	161	75	-1,07	3,56E-02
ENSDARG00000069433	tnk1	266	123	-1,07	3,66E-02
ENSDARG00000044056	ahi1	299	140	-1,07	1,20E-02
ENSDARG00000018967	gabbr1a	426	197	-1,07	3,55E-02
ENSDARG00000042627	nhsl1b	476	221	-1,07	1,71E-02
ENSDARG00000062956	dagla	368	172	-1,07	3,94E-03
ENSDARG00000100374	txnrd3	1249	581	-1,08	9,88E-03
ENSDARG00000014571	ctnnb1	8682	4046	-1,08	5,60E-03
ENSDARG00000077095	si:ch211-160o17.4	1514	705	-1,08	5,95E-03
ENSDARG00000004763	hhla2a.2	560	261	-1,08	2,68E-03
ENSDARG00000098825	farsa	177	81	-1,08	4,38E-02
ENSDARG00000101047	rtcb	144	66	-1,08	4,95E-02
ENSDARG00000024694	myo1b	413	191	-1,08	2,39E-02
ENSDARG00000055377	gnb5b	216	99	-1,08	4,21E-02
ENSDARG00000024874	dock4b	679	316	-1,08	4,15E-03
ENSDARG00000005652	man1a1	312	143	-1,09	2,09E-02
ENSDARG00000032197	klf12b	317	146	-1,09	1,94E-02
ENSDARG00000013704	cers2a	1135	518	-1,09	2,10E-02
ENSDARG00000099547	nbeal1	325	146	-1,09	4,96E-02
ENSDARG00000011819	pcnx	438	199	-1,10	2,44E-02
ENSDARG00000043474	atp2b3a	787	360	-1,10	3,77E-03
ENSDARG00000058237	ssbp3a	640	292	-1,10	1,29E-02
ENSDARG00000000966	ncor2	4415	1993	-1,11	2,58E-02
ENSDARG00000078954	rxrba	169	76	-1,11	4,28E-02
ENSDARG00000086183	vkorc1	561	254	-1,11	1,06E-02
ENSDARG00000063253	hecw2b	77	34	-1,11	2,93E-02
ENSDARG00000036235	zbtb3	186	83	-1,11	2,99E-02
ENSDARG00000041572	zfpm1	229	102	-1,12	3,36E-02
ENSDARG00000018961	gtpbp4	2162	961	-1,12	3,52E-02
ENSDARG00000040334	mat2aa	1132	504	-1,12	2,97E-02
ENSDARG00000069763	etv5a	670	299	-1,12	1,73E-02
ENSDARG00000012788	foxa3	1953	861	-1,12	4,88E-02
ENSDARG00000077499	plekho1b	229	103	-1,12	1,16E-02
ENSDARG00000035264	riok2	266	117	-1,13	3,63E-02
ENSDARG00000076058	gba	551	248	-1,13	3,96E-03
ENSDARG00000055543	appb	1854	825	-1,13	1,64E-02
ENSDARG00000099256	pum1	785	351	-1,13	8,58E-03
ENSDARG00000104450	CR855996.2	1042	465	-1,13	1,03E-02
ENSDARG00000078348	tcf20	1290	577	-1,13	6,48E-03
ENSDARG00000013847	egfra	228	101	-1,13	2,11E-02
ENSDARG00000004246	slit2	405	178	-1,13	4,73E-02
ENSDARG00000060494	eprs	1100	491	-1,13	5,43E-03
ENSDARG00000099747	CABZ01111953.1	120	53	-1,13	2,41E-02
ENSDARG00000053405	sord	149	66	-1,14	2,44E-02
ENSDARG00000016132	keap1a	239	106	-1,14	2,15E-02
ENSDARG00000023600	sh3gl2a	1079	475	-1,14	2,00E-02
ENSDARG00000105503	fndc3a	1889	824	-1,14	3,72E-02
ENSDARG00000035471	si:dkey-220k22.1	511	227	-1,14	3,23E-03
ENSDARG00000060123	ca16b	512	224	-1,14	3,14E-02
ENSDARG00000079326	cabin1	329	143	-1,15	4,11E-02
ENSDARG00000058732	scgn	14591	6441	-1,15	8,02E-03
ENSDARG00000076170	pcsk1nl	12228	5432	-1,15	1,78E-03
ENSDARG00000005774	ddx3b	702	307	-1,15	1,98E-02
ENSDARG00000060484	selenoi	132	57	-1,15	4,79E-02

ENSDARG00000063101	man2a2	1211	536	-1,15	2,73E-03
ENSDARG00000000568	ell	417	184	-1,15	2,32E-03
ENSDARG00000086288	scg3	46790	20777	-1,15	6,01E-04
ENSDARG00000102128	eps8	226	98	-1,15	3,78E-02
ENSDARG00000105255	zbtb4	4803	2109	-1,16	5,75E-03
ENSDARG00000009031	tnikb	259	113	-1,16	9,41E-03
ENSDARG00000030161	ppp1r14bb	383	165	-1,16	1,83E-02
ENSDARG00000056050	kctd17	135	59	-1,17	4,26E-03
ENSDARG00000059939	dab1a	519	223	-1,17	2,75E-02
ENSDARG00000104130	rbm26	224	96	-1,17	2,27E-02
ENSDARG00000000019	ube2h	1013	439	-1,17	6,28E-03
ENSDARG00000071215	palm3	176	75	-1,18	1,66E-02
ENSDARG00000008829	chga	2236	945	-1,18	4,33E-02
ENSDARG00000074905	camk1da	2361	1006	-1,18	2,53E-02
ENSDARG00000112923	CABZ01088036.1	152	65	-1,18	3,48E-02
ENSDARG00000011170	ndrg2	691	295	-1,18	2,57E-02
ENSDARG00000013500	abca2	825	350	-1,18	3,29E-02
ENSDARG00000037018	gab1	978	425	-1,18	5,42E-04
ENSDARG00000003751	lats1	368	158	-1,18	7,22E-03
ENSDARG00000063219	ubap2l	1210	522	-1,18	2,48E-03
ENSDARG00000075230	tet1	321	135	-1,19	3,47E-02
ENSDARG00000060480	ubr1	154	64	-1,19	4,67E-02
ENSDARG00000052331	abcf2b	412	177	-1,19	1,43E-03
ENSDARG00000069360	ppp3r1b	1136	489	-1,19	6,20E-04
ENSDARG00000002037	pfkfb2b	320	137	-1,20	6,95E-03
ENSDARG00000068006	gck	7069	2990	-1,20	1,19E-02
ENSDARG00000069185	celsr1a	566	241	-1,20	4,75E-03
ENSDARG00000025269	pdcd6ip	2062	878	-1,20	3,53E-03
ENSDARG00000016830	rimkla	389	164	-1,20	1,62E-02
ENSDARG00000035187	abl1	213	89	-1,20	4,18E-02
ENSDARG00000020123	coq8a	550	233	-1,20	7,37E-03
ENSDARG00000003169	magi1b	514	218	-1,20	4,29E-03
ENSDARG00000054128	im:7136021	163	68	-1,21	2,90E-02
ENSDARG00000021647	gnai1	1327	563	-1,21	2,32E-03
ENSDARG00000100708	jakmip3	85	35	-1,21	4,78E-02
ENSDARG00000055429	kat6b	725	305	-1,21	7,51E-03
ENSDARG00000103658	hivep1	646	265	-1,22	3,89E-02
ENSDARG00000103288	PDPR	201	83	-1,22	1,55E-02
ENSDARG00000077188	atrnl1a	137	57	-1,22	2,52E-02
ENSDARG00000004386	si:dkey-153k10.9	2837	1179	-1,22	9,36E-03
ENSDARG00000017835	brf1a	98	40	-1,22	4,25E-02
ENSDARG00000070961	lepr	2930	1200	-1,23	2,79E-02
ENSDARG00000055787	dzank1	65	27	-1,23	4,91E-02
ENSDARG00000100524	abca3b	1442	603	-1,23	2,56E-03
ENSDARG00000035808	clcn4	304	126	-1,23	9,14E-03
ENSDARG00000073944	si:ch73-386h18.1	826	340	-1,23	1,33E-02
ENSDARG00000062177	dcbld2	743	303	-1,23	2,65E-02
ENSDARG00000077650	tnksa	1008	415	-1,23	1,28E-02
ENSDARG00000019588	chico	512	207	-1,23	3,97E-02
ENSDARG00000079872	rapgef4	3418	1376	-1,24	4,66E-02
ENSDARG00000059809	caska	166	67	-1,24	4,99E-02
ENSDARG00000014101	pyroxd2	394	163	-1,24	6,15E-03
ENSDARG00000076943	lhfpl4b	250	101	-1,24	3,47E-02
ENSDARG00000001710	flot1b	1610	664	-1,24	6,73E-03
ENSDARG00000069970	lingo2b	478	197	-1,24	1,01E-02
ENSDARG00000032083	dpysl2b	564	233	-1,24	4,86E-03
ENSDARG00000079891	dnajc6	723	297	-1,25	5,10E-03
ENSDARG00000061654	wwc3	87	35	-1,25	3,33E-02
ENSDARG00000062220	plekha7b	131	53	-1,25	2,54E-02
ENSDARG00000101949	numbl	228	93	-1,25	6,27E-03
ENSDARG00000074702	arfgef2	483	196	-1,25	8,76E-03
ENSDARG00000100227	SLC7A1 (1 of many)	166	66	-1,26	2,16E-02
ENSDARG00000101858	DST	391	159	-1,26	8,45E-03
ENSDARG00000102730	mapk10	556	224	-1,26	8,13E-03
ENSDARG00000017880	kcnip3b	615	248	-1,27	1,05E-02
ENSDARG00000004954	grna	2953	1181	-1,27	1,35E-02
ENSDARG00000014891	robo2	1701	678	-1,27	1,74E-02
ENSDARG00000077536	snrnp200	1547	617	-1,27	1,25E-02
ENSDARG00000101986	irf6	328	131	-1,27	1,98E-02
ENSDARG00000043643	ehbp1	1049	424	-1,27	2,05E-03
ENSDARG00000103792	CABZ01076737.1	143	56	-1,27	3,83E-02
ENSDARG00000113609	TMEM164	184	72	-1,27	4,78E-02
ENSDARG00000061732	raph1b	424	168	-1,28	1,43E-02

ENSDARG00000008287	fam114a1	416	165	-1,28	8,84E-03
ENSDARG00000006892	myo3b	391	155	-1,29	9,76E-03
ENSDARG00000058207	abi2a	580	232	-1,29	2,02E-03
ENSDARG00000041702	rfx6	2696	1064	-1,29	1,05E-02
ENSDARG00000076005	piezo2a.2	185	73	-1,29	1,13E-02
ENSDARG00000003008	prkcea	1997	792	-1,29	5,40E-03
ENSDARG00000062008	hs2st1b	637	252	-1,29	4,98E-03
ENSDARG00000104094	grk3	175	68	-1,30	2,82E-02
ENSDARG00000002916	sec31b	308	119	-1,30	2,18E-02
ENSDARG00000063007	apc2	666	259	-1,30	1,36E-02
ENSDARG00000068217	stx2b	666	259	-1,31	1,11E-02
ENSDARG00000087018	FO704748.1	91	35	-1,31	3,43E-02
ENSDARG00000062909	furina	7202	2777	-1,31	1,83E-02
ENSDARG00000091293	dmxl2	1568	608	-1,31	9,40E-03
ENSDARG00000060849	dlgap4a	255	98	-1,32	1,24E-02
ENSDARG00000078311	dcaf6	160	61	-1,32	4,28E-02
ENSDARG00000021242	mvp	1130	430	-1,32	2,02E-02
ENSDARG00000098954	stard13b	185	71	-1,32	1,37E-02
ENSDARG00000031587	flvcr1	1353	526	-1,33	2,23E-03
ENSDARG00000101813	nap1l1	1723	668	-1,33	1,64E-03
ENSDARG00000011855	aak1a	439	163	-1,33	4,83E-02
ENSDARG00000104225	ndel1b	474	183	-1,34	2,26E-03
ENSDARG00000007918	ttc27	185	70	-1,34	1,57E-02
ENSDARG00000060504	pfkla	57	21	-1,34	3,34E-02
ENSDARG00000052658	pptc7b	176	66	-1,34	2,52E-02
ENSDARG00000034443	mpped2	650	247	-1,34	7,80E-03
ENSDARG00000055054	bcl9l	200	76	-1,34	1,06E-02
ENSDARG00000068421	ttc9b	77	29	-1,34	3,64E-02
ENSDARG00000098822	tcerg1a	98	37	-1,34	1,21E-02
ENSDARG00000020845	tns1b	1300	482	-1,35	3,35E-02
ENSDARG00000086990	carmil3	731	275	-1,35	1,51E-02
ENSDARG00000028793	nlk2	511	194	-1,35	7,48E-03
ENSDARG00000036442	atp11c	332	128	-1,35	2,39E-03
ENSDARG00000002131	celf2	244	91	-1,35	2,25E-02
ENSDARG00000012823	dlgap4b	213	78	-1,35	4,88E-02
ENSDARG00000095912	si:dkey-229b18.3	337	129	-1,35	7,05E-04
ENSDARG00000029955	glb1l	159	60	-1,35	1,30E-02
ENSDARG00000004871	mst1rb	1867	696	-1,35	1,69E-02
ENSDARG00000107385	aars2	179	67	-1,35	8,07E-03
ENSDARG00000057032	ppm1nb	197	72	-1,36	2,17E-02
ENSDARG00000006923	cacna1ab	363	132	-1,36	3,90E-02
ENSDARG00000001803	kcnh6a	1251	472	-1,36	2,98E-03
ENSDARG00000115805	GGT7	742	273	-1,37	2,01E-02
ENSDARG00000053279	apln	731	272	-1,37	7,94E-03
ENSDARG00000037320	fam131c	156	57	-1,37	3,57E-02
ENSDARG00000098108	dusp2	7513	2738	-1,37	2,60E-02
ENSDARG00000103428	ogdhb	142	51	-1,37	4,92E-02
ENSDARG00000101270	klhl8	190	70	-1,37	1,64E-02
ENSDARG00000056642	hdac9b	1884	703	-1,37	3,88E-03
ENSDARG00000004592	gpr22a	1345	486	-1,38	2,79E-02
ENSDARG00000060257	zranb1a	504	189	-1,38	6,95E-04
ENSDARG00000100456	ptk2aa	162	59	-1,38	2,55E-02
ENSDARG00000034714	esyt1a	713	259	-1,38	2,12E-02
ENSDARG00000089009	frmpd1b	175	62	-1,38	3,14E-02
ENSDARG00000098375	polr1a	765	283	-1,39	2,88E-03
ENSDARG00000062479	vcam1b	333	119	-1,39	3,17E-02
ENSDARG00000104306	LO018474.1	430	159	-1,40	1,20E-03
ENSDARG00000099425	CABZ01073265.1	3934	1400	-1,40	2,64E-02
ENSDARG00000021833	ahr2	2437	904	-1,40	6,16E-04
ENSDARG00000104676	prkd2	461	170	-1,40	1,76E-03
ENSDARG00000061282	glg1a	1704	617	-1,40	1,05E-02
ENSDARG00000075110	dab2ipb	1180	430	-1,40	5,94E-03
ENSDARG00000062082	hipk3b	1080	396	-1,40	2,60E-03
ENSDARG00000079616	cramp1	309	111	-1,40	1,25E-02
ENSDARG00000069440	dachd	295	106	-1,40	1,56E-02
ENSDARG00000098526	gpr75	221	79	-1,40	1,55E-02
ENSDARG00000042833	rffl	260	94	-1,40	9,28E-03
ENSDARG00000104014	mark1	85	30	-1,40	2,39E-02
ENSDARG00000013063	oxct1a	186	65	-1,41	4,93E-02
ENSDARG00000039486	bag3	466	163	-1,41	4,01E-02
ENSDARG00000074481	ulk1b	1244	448	-1,41	9,91E-03
ENSDARG00000060070	adcy7	568	207	-1,41	2,44E-03
ENSDARG00000037593	prickle2b	1070	388	-1,41	4,28E-03

ENSDARG00000063332	shank3a	311	109	-1,41	2,70E-02
ENSDARG00000088789	mast1b	1220	432	-1,41	1,81E-02
ENSDARG00000074469	amigo3	439	154	-1,41	2,85E-02
ENSDARG00000018206	nck2a	76	26	-1,41	4,71E-02
ENSDARG00000044436	rps6ka4	83	30	-1,41	3,03E-02
ENSDARG00000068143	slc39a6	770	278	-1,41	4,26E-03
ENSDARG00000058421	lgi1b	194	68	-1,42	2,61E-02
ENSDARG00000062485	dock11	379	136	-1,42	6,07E-03
ENSDARG00000021539	ephb2b	545	196	-1,42	5,16E-03
ENSDARG00000007774	vash2	213	76	-1,42	6,78E-03
ENSDARG00000002968	a1cf	3613	1318	-1,42	4,14E-04
ENSDARG00000101478	plppr2a	118	41	-1,42	3,80E-02
ENSDARG00000020718	slc25a22a	652	230	-1,43	1,26E-02
ENSDARG00000029668	crim1	759	268	-1,43	1,02E-02
ENSDARG00000045006	ptprga	1117	403	-1,43	1,15E-03
ENSDARG00000101280	vat1l	226	80	-1,43	9,71E-03
ENSDARG00000100624	CABZ01066312.1	193	67	-1,43	1,55E-02
ENSDARG00000073741	RAP1GDS1	235	82	-1,44	1,82E-02
ENSDARG00000077608	gpr137bb	1263	442	-1,44	1,22E-02
ENSDARG00000054874	gpsm1b	108	37	-1,44	4,50E-02
ENSDARG00000063570	dyrk1aa	480	164	-1,44	3,49E-02
ENSDARG00000102380	cnnm4a	103	35	-1,44	4,53E-02
ENSDARG00000037121	mat2ab	218	76	-1,44	1,99E-02
ENSDARG00000006983	celf3b	339	119	-1,44	9,41E-03
ENSDARG00000024827	rnf150a	157	53	-1,45	4,30E-02
ENSDARG00000009607	ccnc	1049	369	-1,45	3,79E-03
ENSDARG00000044954	slit1a	533	184	-1,45	1,64E-02
ENSDARG00000011545	arrb2b	556	198	-1,45	4,19E-04
ENSDARG00000067916	lyn	221	77	-1,45	8,93E-03
ENSDARG00000020235	sept9a	974	346	-1,45	6,63E-04
ENSDARG00000079291	rapgef3	127	42	-1,45	4,30E-02
ENSDARG00000038754	plk3	794	272	-1,46	1,65E-02
ENSDARG00000078026	zcchc24	104	34	-1,46	3,50E-02
ENSDARG00000099555	foxo1a	3833	1317	-1,46	1,05E-02
ENSDARG00000028322	pex14	782	277	-1,46	3,63E-04
ENSDARG00000114503	phtf2	103	34	-1,46	4,95E-02
ENSDARG00000061862	myo18ab	467	159	-1,46	1,44E-02
ENSDARG00000099221	ppp2r2bb	273	93	-1,46	1,41E-02
ENSDARG00000023448	galnt14	240	82	-1,46	2,28E-02
ENSDARG00000060841	pik3c2a	862	307	-1,47	9,50E-05
ENSDARG00000077710	nlgn1	261	89	-1,47	1,48E-02
ENSDARG00000076974	pdzd7a	114	37	-1,47	4,53E-02
ENSDARG00000071377	hsd11b1la	163	56	-1,47	7,23E-03
ENSDARG00000031075	cadm1a	210	70	-1,47	3,42E-02
ENSDARG00000099483	vdra	78	26	-1,47	4,06E-02
ENSDARG00000011533	sema6dl	195	67	-1,48	7,38E-03
ENSDARG00000060862	atxn1b	678	237	-1,48	5,99E-04
ENSDARG00000013415	lmna	964	327	-1,48	9,09E-03
ENSDARG00000035132	rgs3b	348	118	-1,48	9,13E-03
ENSDARG00000102975	nsg2	3035	1028	-1,48	1,09E-02
ENSDARG00000075733	zyx	136	46	-1,48	7,65E-03
ENSDARG00000030933	ksr1b	700	232	-1,48	2,15E-02
ENSDARG00000098666	si:dkey-93h22.8	105	36	-1,49	7,51E-03
ENSDARG00000079926	syt15	122	40	-1,49	2,23E-02
ENSDARG00000086172	ACVR1C	1152	393	-1,49	3,56E-03
ENSDARG00000061635	myo5aa	522	180	-1,49	1,11E-03
ENSDARG00000004754	hspa4a	415	139	-1,49	1,04E-02
ENSDARG00000051989	tmem187	78	26	-1,49	3,92E-02
ENSDARG00000036107	txnipa	2767	893	-1,49	3,55E-02
ENSDARG00000028000	pfkpa	683	232	-1,49	3,06E-03
ENSDARG00000019743	dctn1a	216	69	-1,49	4,51E-02
ENSDARG00000040177	rgs16	638	212	-1,49	1,50E-02
ENSDARG00000058603	jph1a	141	46	-1,50	3,10E-02
ENSDARG00000042010	pklr	1073	356	-1,50	1,03E-02
ENSDARG00000005343	ccdc85al	163	52	-1,50	4,06E-02
ENSDARG00000031768	roraa	465	152	-1,51	1,99E-02
ENSDARG00000116751	BX537263.4	239	3	-1,51	3,92E-02
ENSDARG00000021059	alas1	815	272	-1,51	5,49E-03
ENSDARG00000103962	add1	641	215	-1,51	3,50E-03
ENSDARG00000032049	enah	258	86	-1,51	4,88E-03
ENSDARG00000055565	cacnb2b	172	55	-1,51	3,51E-02
ENSDARG00000063310	oxr1b	638	204	-1,51	4,08E-02
ENSDARG00000103158	ldb1b	402	128	-1,52	3,59E-02

ENSDARG00000015053	grip1	98	31	-1,52	4,82E-02
ENSDARG00000061203	trpc1	415	134	-1,52	2,50E-02
ENSDARG00000054323	pparab	68	22	-1,52	3,13E-02
ENSDARG00000070230	aldh1l2	113	36	-1,52	1,90E-02
ENSDARG00000020853	galr2a	695	229	-1,53	6,48E-03
ENSDARG00000063539	slc25a15a	129	40	-1,53	3,71E-02
ENSDARG00000018270	olfm1a	128	41	-1,53	2,89E-02
ENSDARG00000059900	tbc1d9	291	95	-1,54	7,16E-03
ENSDARG00000010181	asap2a	158	49	-1,54	4,28E-02
ENSDARG00000031200	ppp2r5cb	1868	621	-1,54	6,53E-04
ENSDARG00000078865	cxxc5a	345	113	-1,54	2,42E-03
ENSDARG00000003449	pde10a	474	154	-1,55	5,62E-03
ENSDARG00000058606	sik1	12091	3896	-1,55	7,81E-03
ENSDARG00000061335	galnt1	641	214	-1,55	2,23E-04
ENSDARG00000029472	afdna	574	187	-1,55	2,78E-03
ENSDARG00000079889	phldb2a	119	37	-1,55	2,65E-02
ENSDARG00000003142	dachc	499	156	-1,56	2,09E-02
ENSDARG00000063594	hipk1a	317	101	-1,56	8,39E-03
ENSDARG00000015824	lemd3	218	71	-1,56	2,54E-03
ENSDARG00000010002	gna11a	183	58	-1,56	7,58E-03
ENSDARG00000103413	zgc:109949	832	256	-1,56	3,08E-02
ENSDARG00000055999	BRD8	102	32	-1,57	2,09E-02
ENSDARG00000015915	slc25a36b	2318	752	-1,57	1,17E-03
ENSDARG00000053311	tespa1	254	82	-1,57	8,69E-04
ENSDARG00000058969	cntnap2a	1173	359	-1,57	2,49E-02
ENSDARG00000101687	CU929259.1	515	162	-1,58	7,96E-03
ENSDARG00000021846	hid1a	476	154	-1,58	9,59E-04
ENSDARG00000093253	si:dkey-229d2.4	45	13	-1,58	3,62E-02
ENSDARG00000069795	zgc:154058	918	288	-1,58	8,14E-03
ENSDARG00000078676	myrf	100	30	-1,58	3,59E-02
ENSDARG00000039881	cemip	107	33	-1,58	1,50E-02
ENSDARG00000086034	nectin1b	196	60	-1,59	1,33E-02
ENSDARG00000104358	FP016005.1	28	8	-1,59	4,55E-02
ENSDARG00000007220	ncam1b	1485	474	-1,59	1,51E-03
ENSDARG00000075904	rfx1b	38	11	-1,60	3,05E-02
ENSDARG00000008859	mylipa	1198	378	-1,60	2,60E-03
ENSDARG00000096701	si:dkey-21e13.3	47	14	-1,60	4,38E-02
ENSDARG00000052113	hexa	1034	325	-1,60	2,57E-03
ENSDARG00000022372	kng1	655	199	-1,60	1,85E-02
ENSDARG00000063167	chkb	1035	322	-1,60	4,54E-03
ENSDARG00000115428	CABZ01101813.1	434	137	-1,60	1,44E-03
ENSDARG00000001154	rimbp2	477	143	-1,60	2,04E-02
ENSDARG00000036548	s1pr2	292	87	-1,60	3,05E-02
ENSDARG00000090997	vegfba	534	166	-1,60	4,55E-03
ENSDARG00000087843	cntn1a	168	51	-1,60	2,26E-02
ENSDARG00000027290	nrp1b	709	211	-1,60	2,73E-02
ENSDARG00000086790	gucy1b1	1203	381	-1,61	5,75E-04
ENSDARG00000074149	itpr1b	598	183	-1,61	8,57E-03
ENSDARG00000101722	larp1	124	36	-1,61	3,83E-02
ENSDARG00000062154	dip2ca	825	256	-1,61	3,79E-03
ENSDARG00000088581	f10	556	171	-1,61	5,21E-03
ENSDARG00000011065	camk2b1	882	274	-1,61	3,06E-03
ENSDARG00000100274	WDR7	421	127	-1,61	1,59E-02
ENSDARG00000010957	mpp2b	372	115	-1,62	2,93E-03
ENSDARG00000098405	map3k1	488	147	-1,62	1,44E-02
ENSDARG00000040380	arhgef1a	697	208	-1,62	1,53E-02
ENSDARG00000059064	rassf3	173	52	-1,62	1,41E-02
ENSDARG00000075831	slc7a8a	406	116	-1,62	3,95E-02
ENSDARG00000057007	ctbp1	3435	1078	-1,63	3,12E-04
ENSDARG00000090787	klhdc8b	184	53	-1,63	3,13E-02
ENSDARG00000079742	mcf2l2	393	121	-1,63	2,57E-03
ENSDARG00000004176	ulk4	171	53	-1,63	3,07E-03
ENSDARG00000116666	CABZ01078320.1	28	8	-1,63	4,22E-02
ENSDARG00000052468	GSK3B (1 of many)	340	102	-1,63	1,33E-02
ENSDARG00000017294	gnai2b	1158	352	-1,63	4,81E-03
ENSDARG00000040064	acp6	563	170	-1,63	5,91E-03
ENSDARG00000073688	nav2a	1089	330	-1,63	6,41E-03
ENSDARG00000091326	cep120	92	27	-1,64	1,51E-02
ENSDARG00000010385	sept4a	501	142	-1,64	3,78E-02
ENSDARG00000074752	hlfa	626	188	-1,64	8,02E-03
ENSDARG00000079634	filip1b	51	15	-1,64	1,42E-02
ENSDARG00000053636	cracr2b	117	33	-1,64	3,18E-02
ENSDARG00000059747	kcnj19a	526	153	-1,64	2,01E-02

ENSDARG00000079283	si:ch211-247j9.1	446	132	-1,64	1,22E-02
ENSDARG00000010318	srpx	41	12	-1,65	3,17E-02
ENSDARG00000038106	slc37a4a	490	148	-1,65	2,41E-03
ENSDARG00000012496	GPR62 (1 of many)	299	89	-1,65	6,48E-03
ENSDARG00000043055	hiat1b	1057	328	-1,65	8,55E-05
ENSDARG00000093093	adam17b	458	136	-1,66	6,27E-03
ENSDARG00000057419	slc44a5b	1354	407	-1,66	2,27E-03
ENSDARG00000035859	angptl4	1562	444	-1,66	1,86E-02
ENSDARG00000034093	dclk2a	290	86	-1,67	3,79E-03
ENSDARG00000079396	lrfn5b	113	33	-1,67	1,43E-02
ENSDARG00000110940	ptprsa	249	70	-1,67	3,02E-02
ENSDARG00000102636	trmt9b	1151	317	-1,67	4,04E-02
ENSDARG00000056196	slc2a2	2018	574	-1,67	1,53E-02
ENSDARG00000077839	dhx30	107	30	-1,68	1,87E-02
ENSDARG00000078560	slc9a5	199	55	-1,69	2,44E-02
ENSDARG00000079835	man2c1	1756	495	-1,69	1,30E-02
ENSDARG00000036826	ankrd52a	157	45	-1,69	1,38E-02
ENSDARG00000045803	mfge8b	5393	1539	-1,69	8,17E-03
ENSDARG00000102125	iqsec2a	501	136	-1,70	3,06E-02
ENSDARG00000021735	cacna1ba	389	112	-1,70	5,81E-03
ENSDARG00000104549	arhgap44	264	75	-1,70	1,15E-02
ENSDARG00000102610	R3HDM2	181	51	-1,70	8,93E-03
ENSDARG00000054749	lmo4b	218	58	-1,70	3,11E-02
ENSDARG00000032990	plxnb3	58	15	-1,70	3,59E-02
ENSDARG00000113657	LO018363.2	101	28	-1,71	1,99E-02
ENSDARG00000028857	sgsm1a	2425	685	-1,71	7,47E-03
ENSDARG00000018716	dgkh	647	187	-1,71	1,42E-03
ENSDARG00000087299	armc9	166	48	-1,71	2,20E-03
ENSDARG00000111110	CABZ01086611.1	797	232	-1,71	1,08E-03
ENSDARG00000036058	gnao1b	843	244	-1,71	1,21E-03
ENSDARG00000023659	gabpb1	39	10	-1,72	3,08E-02
ENSDARG00000056874	lygl1	734	209	-1,72	3,56E-03
ENSDARG00000029952	ampd2b	787	232	-1,72	1,75E-04
ENSDARG00000075334	arhgap32a	132	36	-1,72	1,42E-02
ENSDARG00000098683	zmiz2	581	164	-1,72	3,96E-03
ENSDARG00000102056	si:dkeyp-94b4.1	225	64	-1,72	2,73E-03
ENSDARG00000073952	slc4a7	618	172	-1,73	7,96E-03
ENSDARG00000063149	tmtc1	818	224	-1,73	1,15E-02
ENSDARG00000014081	fam184a	533	151	-1,73	2,52E-03
ENSDARG00000104101	si:ch73-287m6.1	643	184	-1,73	1,97E-03
ENSDARG00000077228	ntrk3a	442	117	-1,73	2,42E-02
ENSDARG00000063578	CABZ01020835.1	152	42	-1,73	8,77E-03
ENSDARG00000042859	slc5a6a	154	43	-1,73	8,50E-03
ENSDARG00000104573	slc12a2	624	179	-1,73	7,23E-04
ENSDARG00000006257	vldlr	576	163	-1,73	1,58E-03
ENSDARG00000010267	dpydb	130	35	-1,74	1,50E-02
ENSDARG00000062812	glsa	1103	305	-1,74	5,60E-03
ENSDARG00000002791	atp1a1a.1	1857	528	-1,74	7,63E-04
ENSDARG00000018259	atp1a3a	2852	802	-1,74	1,67E-03
ENSDARG00000058108	eps8l2	240	66	-1,75	7,37E-03
ENSDARG00000099802	pnp5b	134	37	-1,75	6,63E-03
ENSDARG00000021938	smad9	147	41	-1,75	4,19E-03
ENSDARG00000007494	st3gal8	733	200	-1,75	6,73E-03
ENSDARG00000002731	sdc2	1627	463	-1,75	3,29E-04
ENSDARG00000101606	rims2a	3667	986	-1,75	8,66E-03
ENSDARG00000102765	lonp1	175	48	-1,75	8,01E-03
ENSDARG00000096637	si:dkey-11c5.11	100	26	-1,76	2,12E-02
ENSDARG00000017391	unc13ba	138	37	-1,76	1,05E-02
ENSDARG00000009677	dlg1	957	262	-1,76	3,76E-03
ENSDARG00000044295	pip5k1ba	199	54	-1,76	4,61E-03
ENSDARG00000070903	met	64	16	-1,76	3,49E-02
ENSDARG00000094696	si:dkey-201c13.2	34	9	-1,76	3,38E-02
ENSDARG00000087921	plce1	1181	322	-1,77	3,50E-03
ENSDARG00000101210	glg1b	220	60	-1,77	3,39E-03
ENSDARG00000057227	si:dkey-222b8.4	17	4	-1,77	2,83E-02
ENSDARG00000076386	epdl1	982	242	-1,78	3,77E-02
ENSDARG00000012914	arhgef4	446	121	-1,78	2,41E-03
ENSDARG00000054448	kif1c	106	27	-1,78	2,43E-02
ENSDARG00000055540	pfkfb4a	2144	575	-1,79	4,30E-03
ENSDARG00000075083	prkdc	1239	326	-1,79	6,63E-03
ENSDARG00000026376	aco1	414	112	-1,79	1,76E-03
ENSDARG00000037495	rtn4rl2b	3396	794	-1,80	4,64E-02
ENSDARG00000017127	pdzk1ip1	947	255	-1,80	2,32E-03

ENSDARG00000111069	lgi2a	940	225	-1,80	4,94E-02
ENSDARG00000061099	nfasca	1744	478	-1,80	4,19E-04
ENSDARG00000036008	caly	2096	526	-1,80	1,79E-02
ENSDARG00000043342	gpx3	10911	2959	-1,80	8,43E-04
ENSDARG00000020387	nipsnap3a	275	71	-1,80	9,25E-03
ENSDARG00000030357	zgc:66313	899	228	-1,81	1,32E-02
ENSDARG00000029859	si:ch211-241d21.5	65	16	-1,81	3,36E-02
ENSDARG00000058232	fbrsl1	433	118	-1,81	3,03E-04
ENSDARG00000017929	ncoa2	2490	669	-1,82	7,46E-04
ENSDARG00000062190	PDE3B	1188	319	-1,82	6,00E-04
ENSDARG00000001634	kirrel1a	58	15	-1,83	1,16E-02
ENSDARG00000102583	il4r.1	209	48	-1,83	3,37E-02
ENSDARG00000028507	itgb4	76	18	-1,83	3,50E-02
ENSDARG00000007753	cpne2	629	156	-1,83	1,19E-02
ENSDARG00000076416	shroom2a	584	158	-1,83	1,13E-04
ENSDARG00000062352	sema4ab	152	37	-1,83	2,44E-02
ENSDARG00000078748	si:ch211-137a8.4	632	167	-1,84	7,54E-04
ENSDARG00000009493	MAP3K13	185	44	-1,84	2,44E-02
ENSDARG00000054186	ncs1b	158	41	-1,84	2,94E-03
ENSDARG00000071005	ppp1r3ca	289	65	-1,84	4,74E-02
ENSDARG00000026028	ankrd44	85	21	-1,84	1,37E-02
ENSDARG00000036424	pcdh20	1497	399	-1,85	1,76E-04
ENSDARG00000079251	nlgn2b	207	52	-1,85	1,17E-02
ENSDARG00000102051	mtbl	1495	383	-1,85	2,80E-03
ENSDARG00000112870	CABZ01021599.1	20	5	-1,86	3,67E-02
ENSDARG00000112961	LO017848.1	39	9	-1,86	2,65E-02
ENSDARG00000043154	ucp2	5212	1314	-1,86	3,78E-03
ENSDARG00000058646	ptprna	9392	2479	-1,86	1,34E-04
ENSDARG00000063307	sgsm2	251	64	-1,86	2,41E-03
ENSDARG00000068194	klf9	736	176	-1,87	1,37E-02
ENSDARG00000075821	plxnb1a	1246	316	-1,87	2,07E-03
ENSDARG00000023694	spon1b	6256	1663	-1,87	1,42E-05
ENSDARG00000051861	pkp3a	305	74	-1,87	9,27E-03
ENSDARG00000016774	plekho2	355	78	-1,88	4,01E-02
ENSDARG00000008026	fam129bb	193	48	-1,88	3,33E-03
ENSDARG00000101413	dusp7	1258	321	-1,88	6,82E-04
ENSDARG00000070809	znf516	67	16	-1,89	9,12E-03
ENSDARG00000104068	gstp1	500	119	-1,89	9,00E-03
ENSDARG00000078425	oat	238	56	-1,89	1,30E-02
ENSDARG00000040633	PRMT8	77	19	-1,89	2,89E-03
ENSDARG00000035660	si:ch211-193k19.1	703	176	-1,90	1,43E-03
ENSDARG00000061976	sema6bb	326	78	-1,90	7,13E-03
ENSDARG00000014556	serpinb1l3	879	213	-1,90	4,26E-03
ENSDARG00000079453	atp8b4	415	104	-1,90	7,56E-04
ENSDARG00000010946	cbsb	223	48	-1,91	3,05E-02
ENSDARG00000057708	zgc:175264	2586	665	-1,91	6,58E-05
ENSDARG00000079962	doc2d	455	105	-1,91	1,49E-02
ENSDARG00000062135	sytl5	167	41	-1,91	2,44E-03
ENSDARG00000069028	rxfp3.3a1	369	81	-1,91	3,29E-02
ENSDARG00000021611	si:dkey-151g10.3	368	92	-1,91	7,78E-04
ENSDARG00000090617	ctif	108	23	-1,91	3,70E-02
ENSDARG00000111920	cplx1	56	12	-1,91	2,12E-02
ENSDARG00000102715	UST (1 of many)	56	12	-1,91	2,42E-02
ENSDARG00000080021	zmat3	28	6	-1,91	4,47E-02
ENSDARG00000077920	CSMD3 (1 of many)	729	168	-1,92	1,39E-02
ENSDARG00000009930	cadm2a	4102	981	-1,92	4,15E-03
ENSDARG00000002779	pdx1	17171	3619	-1,93	3,44E-02
ENSDARG00000096304	btr31	107	26	-1,93	2,29E-03
ENSDARG00000107858	CABZ01073424.1	154	36	-1,94	8,04E-03
ENSDARG00000089458	rp1l1a	37	8	-1,94	2,67E-02
ENSDARG00000101482	hk2	46	9	-1,94	4,05E-02
ENSDARG00000006474	saxo2	45	9	-1,95	3,80E-02
ENSDARG00000059202	tspan2b	47	10	-1,95	3,26E-02
ENSDARG00000079869	si:ch211-218o21.4	91	19	-1,95	2,64E-02
ENSDARG00000031317	ppdpfb	28857	6643	-1,95	4,16E-03
ENSDARG00000014587	slc38a5b	398	91	-1,95	7,71E-03
ENSDARG00000045911	tulp4a	660	151	-1,95	7,18E-03
ENSDARG00000078729	ackr4a	95	21	-1,95	1,22E-02
ENSDARG00000070683	dkk3b	132721	29215	-1,95	1,37E-02
ENSDARG00000060113	znf395a	901	207	-1,96	4,81E-03
ENSDARG00000034086	zgc:65895	434	101	-1,96	4,54E-03
ENSDARG00000075188	adamts10	32	7	-1,96	3,88E-02
ENSDARG00000052765	gria2b	995	245	-1,97	3,44E-05

ENSDARG00000069407	zgc:194990	1426	324	-1,97	4,92E-03
ENSDARG00000017365	slc23a2	1761	433	-1,97	2,36E-05
ENSDARG00000031422	igfbp2b	2610	613	-1,98	9,30E-04
ENSDARG00000061497	thsd7bb	122	25	-1,98	2,34E-02
ENSDARG00000002607	unm_sa1614	1123	257	-1,99	2,78E-03
ENSDARG00000004634	osbp	512	113	-1,99	7,83E-03
ENSDARG00000107441	CABZ01063170.1	251	56	-1,99	7,56E-03
ENSDARG00000042677	cadm1b	493	112	-1,99	3,25E-03
ENSDARG00000052734	hmgcra	303	61	-1,99	2,15E-02
ENSDARG00000014967	g6pcb	8208	1085	-1,99	4,16E-02
ENSDARG00000006065	znf385b	79	17	-1,99	1,81E-02
ENSDARG00000001912	drd4-rs	333	62	-1,99	4,67E-02
ENSDARG00000077709	iqsec2b	78	16	-2,00	3,66E-02
ENSDARG00000045644	ca12	3468	750	-2,00	8,77E-03
ENSDARG00000114329	HOOK3	41	8	-2,00	3,01E-02
ENSDARG00000090914	si:ch211-117k10.3	73	16	-2,01	7,71E-03
ENSDARG00000077083	avpr1aa	4343	904	-2,01	1,73E-02
ENSDARG00000077523	tdrd7b	114	25	-2,01	6,61E-03
ENSDARG00000069765	syngap1b	84	17	-2,02	3,64E-02
ENSDARG00000038574	scg2b	13267	3094	-2,02	1,90E-04
ENSDARG00000074184	arhgap32b	713	163	-2,02	6,52E-04
ENSDARG00000063649	tead3b	131	30	-2,02	1,72E-03
ENSDARG00000021924	hsp70.3	3483	682	-2,02	3,08E-02
ENSDARG00000112868	CABZ01073424.2	74	16	-2,02	6,61E-03
ENSDARG00000102888	gpr39	72	15	-2,02	1,02E-02
ENSDARG00000099985	cyr61l2	458	98	-2,02	7,03E-03
ENSDARG00000061011	hlfb	233	49	-2,03	9,44E-03
ENSDARG00000054318	stk33	327	70	-2,03	6,07E-03
ENSDARG00000067859	scospondin	91	18	-2,03	3,40E-02
ENSDARG00000000472	cntn2	215	46	-2,03	4,87E-03
ENSDARG00000115832	LO018490.1	26	6	-2,05	4,98E-03
ENSDARG00000063371	rasa3	44	8	-2,06	2,36E-02
ENSDARG00000052000	cav2	1117	242	-2,06	2,01E-03
ENSDARG00000104480	hcn1	205	41	-2,06	1,53E-02
ENSDARG00000100170	pam	15650	3575	-2,07	3,66E-05
ENSDARG00000088168	ablim3	53	10	-2,07	1,99E-02
ENSDARG00000009949	fndc4b	857	175	-2,07	7,78E-03
ENSDARG00000077022	fam131a	101	22	-2,07	2,60E-03
ENSDARG00000018896	mmp24	39	7	-2,07	2,86E-02
ENSDARG00000022668	grapb	712	134	-2,08	1,75E-02
ENSDARG00000096739	si:dkey-219e21.2	76	15	-2,08	1,08E-02
ENSDARG00000077656	arfgef3	3627	780	-2,08	1,11E-03
ENSDARG00000103554	notch1a	118	25	-2,08	2,89E-03
ENSDARG00000015495	klf3	337	65	-2,09	1,91E-02
ENSDARG00000075851	oafa	159	31	-2,09	9,91E-03
ENSDARG00000005541	wif1	3501	694	-2,09	8,39E-03
ENSDARG00000060601	rgs7bpa	63	11	-2,09	4,27E-02
ENSDARG00000020759	elf1	1747	386	-2,10	1,12E-04
ENSDARG00000061549	foxo1b	42	7	-2,10	2,67E-02
ENSDARG00000061140	colec12	55	9	-2,11	2,97E-02
ENSDARG00000063726	cdk12	892	194	-2,11	1,86E-04
ENSDARG00000100606	BX539336.1	484	101	-2,11	1,84E-03
ENSDARG00000074319	sall1a	3009	600	-2,11	4,81E-03
ENSDARG00000062204	sigirr	1066	221	-2,11	1,76E-03
ENSDARG00000092660	cyp27c1	178	31	-2,11	2,15E-02
ENSDARG00000077569	lrrn3a	214	45	-2,11	9,29E-04
ENSDARG00000024365	crlf1a	105	21	-2,12	3,94E-03
ENSDARG00000039500	LRFN3	101	20	-2,12	5,96E-03
ENSDARG00000103479	ptprfa	636	127	-2,12	3,84E-03
ENSDARG00000088083	sik3	539	102	-2,12	1,29E-02
ENSDARG00000070047	rgs4	828	172	-2,12	9,64E-04
ENSDARG00000060222	scn1ba	38	6	-2,13	4,91E-02
ENSDARG00000035832	pyyb	8838	1689	-2,13	8,24E-03
ENSDARG00000056978	letm1	2238	475	-2,13	2,29E-04
ENSDARG00000025024	pnoca	20643	3844	-2,14	9,79E-03
ENSDARG00000033916	si:ch211-243j20.2	1928	391	-2,14	1,53E-03
ENSDARG00000002644	rgs5a	77783	14972	-2,15	5,25E-03
ENSDARG00000074182	prokr1a	353	70	-2,15	2,39E-03
ENSDARG00000021806	zfp36l2	136	25	-2,15	1,15E-02
ENSDARG00000042282	itga6a	496	102	-2,15	4,52E-04
ENSDARG00000045139	ca7	1365	250	-2,16	1,07E-02
ENSDARG00000063133	slc4a10a	585	120	-2,16	5,03E-04
ENSDARG00000063433	atp2b2	174	34	-2,16	2,69E-03

ENSDARG00000102687	CABZ01085139.1	535	73	-2,16	2,52E-02
ENSDARG00000061096	dapk2a	159	30	-2,16	6,75E-03
ENSDARG00000052011	rrad	292	48	-2,16	2,02E-02
ENSDARG00000095743	sox11b	636	115	-2,17	1,27E-02
ENSDARG00000109663	LO016987.2	51	10	-2,17	6,65E-03
ENSDARG00000018393	kank2	143	30	-2,17	9,98E-05
ENSDARG00000102972	CABZ01075125.1	95	16	-2,17	2,27E-02
ENSDARG00000035009	trim35-27	38	7	-2,18	2,23E-02
ENSDARG00000032206	cthl	506	73	-2,18	4,31E-02
ENSDARG00000088709	tnfaip8l3	211	41	-2,18	1,84E-03
ENSDARG00000101703	zgc:158260	35	5	-2,18	3,43E-02
ENSDARG00000060415	arhgef28	1119	227	-2,18	3,77E-04
ENSDARG00000071042	cx28.8	319	60	-2,19	6,59E-03
ENSDARG00000015003	arhgap4b	31	5	-2,19	4,06E-02
ENSDARG00000042529	gnat2	38	6	-2,19	3,14E-02
ENSDARG00000016858	smad7	220	43	-2,19	1,11E-03
ENSDARG00000033840	fat3a	111	17	-2,20	4,08E-02
ENSDARG00000067912	MAN1C1	65	11	-2,20	1,30E-02
ENSDARG00000045594	shisal1a	1711	345	-2,20	1,75E-04
ENSDARG00000023797	ryr1b	40	6	-2,20	3,56E-02
ENSDARG00000091548	stard9	51	8	-2,21	2,28E-02
ENSDARG00000059616	hs3st2	93	16	-2,21	1,79E-02
ENSDARG00000060678	ndst2b	268	52	-2,21	8,47E-04
ENSDARG00000092638	ptprua	1512	291	-2,22	1,11E-03
ENSDARG00000033327	unc5b	123	21	-2,22	1,15E-02
ENSDARG00000099096	gabrb4	175	33	-2,22	2,23E-03
ENSDARG00000044415	tlr5a	151	26	-2,22	1,24E-02
ENSDARG00000105657	si:cabz01080528.1	62	11	-2,22	9,34E-03
ENSDARG00000008127	pcdh15b	335	64	-2,22	8,47E-04
ENSDARG00000057826	si:ch73-61d6.3	112	20	-2,23	6,49E-03
ENSDARG00000069014	nphp4	311	61	-2,23	5,69E-04
ENSDARG00000100594	sez6l	206	32	-2,23	2,07E-02
ENSDARG00000088100	KCNIP4	343	62	-2,24	3,88E-03
ENSDARG00000061328	cdon	82	14	-2,24	3,60E-03
ENSDARG00000103205	si:ch211-161f7.2	22	3	-2,24	3,35E-02
ENSDARG00000074921	fam117ba	87	14	-2,24	1,73E-02
ENSDARG00000004576	plk4	84	11	-2,25	3,33E-02
ENSDARG00000092362	hsp70.2	1993	329	-2,25	1,63E-02
ENSDARG00000014320	gucy2c	782	148	-2,25	6,01E-04
ENSDARG00000086214	ntrk3b	438	77	-2,25	4,14E-03
ENSDARG00000099079	aclya	3419	670	-2,25	9,98E-05
ENSDARG00000021909	VSTM2A	145	27	-2,25	3,27E-03
ENSDARG00000075817	mettl24	129	22	-2,25	9,64E-03
ENSDARG00000079618	sik2a	211	39	-2,26	1,08E-03
ENSDARG00000062621	satb1b	23	3	-2,26	3,59E-02
ENSDARG00000076998	si:ch73-92i20.1	34	5	-2,26	1,18E-02
ENSDARG00000067824	cntnap3	25	3	-2,26	4,82E-02
ENSDARG00000030376	grin2bb	295	54	-2,27	1,60E-03
ENSDARG00000024654	pgm3	1746	317	-2,28	9,59E-04
ENSDARG00000061248	xylt1	424	74	-2,28	3,48E-03
ENSDARG00000044161	grid1b	632	108	-2,28	4,23E-03
ENSDARG00000100043	CABZ01111454.1	26	4	-2,28	3,06E-02
ENSDARG00000079414	sez6b	848	136	-2,29	1,94E-02
ENSDARG00000086057	plxnd1	100	11	-2,29	3,47E-02
ENSDARG00000098408	stbd1	56	9	-2,29	1,40E-02
ENSDARG00000054321	ngs	29	3	-2,29	3,72E-02
ENSDARG00000079253	plch1	71	10	-2,30	2,12E-02
ENSDARG00000077047	ptprnb	16370	3081	-2,30	1,29E-04
ENSDARG00000025902	plekhg5a	466	86	-2,30	4,52E-04
ENSDARG00000098578	pdgfab	298	51	-2,30	3,10E-03
ENSDARG00000018065	ntm	158	26	-2,31	8,14E-03
ENSDARG00000087753	CR388166.1	48	6	-2,32	3,88E-02
ENSDARG00000015515	mc4r	444	75	-2,32	3,50E-03
ENSDARG00000077596	phf24	145	22	-2,32	1,55E-02
ENSDARG00000062415	ctnnd2a	71	11	-2,32	9,32E-03
ENSDARG00000100128	CU861477.1	192	30	-2,32	2,43E-02
ENSDARG00000035875	tmem54b	523	83	-2,32	1,32E-02
ENSDARG00000019945	ptprdb	975	181	-2,33	9,31E-05
ENSDARG00000041616	b3gnt9	38	5	-2,33	1,62E-02
ENSDARG00000055854	nr4a3	241	39	-2,33	6,25E-03
ENSDARG00000071230	lrfn5a	167	29	-2,33	1,15E-03
ENSDARG00000042368	kif26aa	155	26	-2,33	4,89E-03
ENSDARG00000043317	kita	263	40	-2,34	9,13E-03

ENSDARG00000092199	si:ch211-250c4.4	543	83	-2,34	7,33E-03
ENSDARG00000103390	cacna2d2a	1930	346	-2,34	3,37E-04
ENSDARG00000028824	slc34a1a	428	72	-2,34	2,60E-03
ENSDARG00000060372	plxna2	97	15	-2,34	8,31E-03
ENSDARG00000071772	si:ch211-253p2.2	110	19	-2,35	8,69E-04
ENSDARG00000096327	cd164l2	137	20	-2,35	8,19E-03
ENSDARG00000039453	dhrs7ca	181	24	-2,35	3,51E-02
ENSDARG00000071493	olfm3a	103	11	-2,35	3,02E-02
ENSDARG00000019418	kcnj2a	1372	244	-2,35	2,29E-04
ENSDARG00000089172	si:ch1073-391i24.1	144	23	-2,35	5,88E-03
ENSDARG00000100166	si:dkeyp-9d4.3	2789	484	-2,36	4,76E-04
ENSDARG00000061081	arpp21	949	155	-2,36	2,18E-03
ENSDARG00000019096	myl7	87	11	-2,36	2,31E-02
ENSDARG00000104296	zgc:165508	321	52	-2,36	3,22E-03
ENSDARG00000041051	mid1ip1a	28	2	-2,37	2,74E-02
ENSDARG00000036541	rhbdf1a	86	14	-2,37	1,59E-03
ENSDARG00000104574	pcsk6	680	103	-2,37	7,16E-03
ENSDARG00000067545	adam19b	370	53	-2,37	1,38E-02
ENSDARG00000079858	tmem163a	189	27	-2,37	1,53E-02
ENSDARG00000078169	cacna2d2b	693	122	-2,37	1,94E-04
ENSDARG00000074317	fam20ca	92	14	-2,38	7,59E-03
ENSDARG00000062199	si:dkey-217l24.1	142	23	-2,38	1,89E-03
ENSDARG00000043009	fam43a	87	12	-2,38	9,34E-03
ENSDARG00000055539	epdl2	38	1	-2,38	4,32E-02
ENSDARG00000093081	si:ch73-92i20.1	68	10	-2,38	9,97E-03
ENSDARG00000024017	mdga2a	168	28	-2,39	1,11E-03
ENSDARG00000023302	gpbar1	219	31	-2,40	1,14E-02
ENSDARG00000037142	zgc:153146	22	3	-2,40	3,79E-02
ENSDARG00000005754	ptprfb	1424	252	-2,40	2,08E-05
ENSDARG00000100313	pip5k1cb	114	17	-2,41	2,68E-03
ENSDARG00000034105	mtus1b	823	130	-2,41	2,29E-03
ENSDARG00000060010	iqgap2	458	76	-2,42	4,76E-04
ENSDARG00000074335	boc	1192	173	-2,42	7,47E-03
ENSDARG00000101660	sntg2	870	142	-2,42	7,32E-04
ENSDARG00000089858	cobll1a	133	19	-2,42	4,80E-03
ENSDARG00000051836	si:dkeyp-19e1.3	33	4	-2,42	2,40E-02
ENSDARG00000016868	rhobtb2a	492	84	-2,42	1,07E-04
ENSDARG00000029177	lnx2a	2896	483	-2,43	2,95E-04
ENSDARG00000098070	CABZ01085140.1	160	21	-2,43	8,33E-03
ENSDARG00000114159	TMEM196 (1 of ma	36	4	-2,43	1,32E-02
ENSDARG00000076294	klb	1499	221	-2,43	4,30E-03
ENSDARG00000069978	cd28l	37	4	-2,43	3,62E-02
ENSDARG00000014680	znf710a	65	8	-2,44	1,99E-02
ENSDARG00000104100	bmpr1ba	62	8	-2,44	1,83E-02
ENSDARG00000027724	dgkb	110	16	-2,44	4,98E-03
ENSDARG00000038489	b3gnt7	84	12	-2,44	4,52E-03
ENSDARG00000024744	dpp6b	440	71	-2,45	4,19E-04
ENSDARG00000016936	hmcn1	597	97	-2,45	3,63E-04
ENSDARG00000089444	omgb	93	12	-2,46	2,04E-02
ENSDARG00000103396	znf804b	23	3	-2,46	1,57E-02
ENSDARG00000116487	CABZ01039304.1	49	7	-2,46	1,04E-02
ENSDARG00000029439	atp2a2a	1163	189	-2,46	2,49E-04
ENSDARG00000041926	dlg4a	266	40	-2,46	2,00E-03
ENSDARG00000025522	sgk1	207	26	-2,47	8,39E-03
ENSDARG00000102491	gpr142	692	109	-2,47	4,88E-04
ENSDARG00000074212	SLC5A10	21710	3251	-2,47	1,71E-03
ENSDARG00000010654	arhgap42b	417	66	-2,47	6,34E-04
ENSDARG00000012078	meis1b	1553	246	-2,47	3,63E-04
ENSDARG00000022254	prkcbb	554	81	-2,47	2,30E-03
ENSDARG00000079985	nrip2	557	84	-2,48	1,68E-03
ENSDARG00000059854	CABZ01041604.1	35	4	-2,49	2,65E-02
ENSDARG00000030107	si:ch211-207i1.2	414	63	-2,49	7,37E-04
ENSDARG00000030411	crygn2	28	4	-2,49	7,12E-03
ENSDARG00000061031	dner	38	4	-2,49	2,39E-02
ENSDARG00000042128	pacsin1b	76	11	-2,50	1,48E-03
ENSDARG00000052648	hs3st4	61	7	-2,50	1,43E-02
ENSDARG00000059368	gria4b	542	84	-2,51	2,65E-04
ENSDARG00000074219	map3k20	155	23	-2,52	8,45E-04
ENSDARG00000031426	csrnp1a	2616	392	-2,52	6,73E-04
ENSDARG00000099133	si:ch73-72b7.1	70	10	-2,52	8,24E-03
ENSDARG00000104930	si:dkey-225f23.5	357	52	-2,52	1,42E-03
ENSDARG00000039959	gdnfa	130	15	-2,52	9,63E-03
ENSDARG00000103535	SORCS3	102	14	-2,53	3,64E-03

ENSDARG00000074919	BFSP1	26	2	-2,54	3,66E-02
ENSDARG00000076702	CABZ01068356.1	303	45	-2,54	4,19E-04
ENSDARG00000006093	cdk15	315	46	-2,54	8,48E-04
ENSDARG00000059945	sv2a	958	147	-2,54	1,09E-04
ENSDARG00000077864	rasgrp3	246	33	-2,55	2,02E-03
ENSDARG00000090844	CABZ01054965.1	211	31	-2,55	3,33E-04
ENSDARG00000098312	scarb2a	75	4	-2,55	1,85E-02
ENSDARG00000073866	tdp1	135	18	-2,56	1,90E-03
ENSDARG00000104326	si:dkey-183i3.9	677	95	-2,56	1,03E-03
ENSDARG00000095223	si:ch211-242f23.3	82	10	-2,57	8,77E-03
ENSDARG00000101178	sh3rf2	173	25	-2,57	4,33E-04
ENSDARG00000113572	si:dkey-102c8.3	80	3	-2,57	3,13E-02
ENSDARG00000011190	fgfr1b	1308	176	-2,58	2,20E-03
ENSDARG00000058508	cfap70	30	4	-2,58	1,27E-02
ENSDARG00000006760	slc24a3	72	10	-2,58	2,78E-03
ENSDARG00000041791	mgat4c	105	14	-2,58	2,31E-03
ENSDARG00000116937	BX927333.1	2102	301	-2,58	4,19E-04
ENSDARG00000070338	hoxc4a	70	7	-2,59	8,84E-03
ENSDARG00000115651	LO017917.1	65	8	-2,60	4,14E-03
ENSDARG00000037639	nkx3.2	300	33	-2,60	6,17E-03
ENSDARG00000060103	cpeb3	245	35	-2,60	3,71E-04
ENSDARG00000069995	dpy19l1l	357	50	-2,61	5,03E-04
ENSDARG00000032849	ndrg1a	1493	207	-2,62	4,34E-04
ENSDARG00000071009	kif20ba	359	46	-2,62	2,31E-03
ENSDARG00000103903	LO017650.1	60	7	-2,64	1,19E-02
ENSDARG00000045979	zgc:153704	225	32	-2,64	2,29E-04
ENSDARG00000100529	sh3bp5a	297	40	-2,64	5,57E-04
ENSDARG00000008723	prkcba	42	4	-2,64	3,67E-02
ENSDARG00000099732	gfra1a	4513	472	-2,64	1,49E-02
ENSDARG00000101471	fgd4a	664	82	-2,64	2,29E-03
ENSDARG00000007398	lrrk1	1018	133	-2,65	1,20E-03
ENSDARG00000101508	nr6a1a	122	14	-2,66	4,66E-03
ENSDARG00000074915	etaa1	249	31	-2,66	8,47E-04
ENSDARG00000098641	nbeal2	395	53	-2,66	4,89E-04
ENSDARG00000099708	cacna1hb	20	0	-2,66	1,90E-02
ENSDARG00000078145	si:ch211-218g4.2	544	69	-2,66	1,18E-03
ENSDARG00000115504	CABZ01080056.1	19	1	-2,67	2,75E-02
ENSDARG00000099038	kcng1	100	10	-2,67	1,31E-02
ENSDARG00000063158	CABZ01081780.1	256	31	-2,68	1,42E-03
ENSDARG00000059340	dlgap2a	233	32	-2,69	1,36E-04
ENSDARG00000062712	tmem264	39	4	-2,69	3,26E-02
ENSDARG00000055305	ret	10653	1498	-2,70	2,18E-05
ENSDARG00000021305	slc30a2	1650	214	-2,70	3,84E-04
ENSDARG00000091277	adra2da	675	81	-2,70	2,02E-03
ENSDARG00000070522	cacna1i	575	74	-2,70	5,42E-04
ENSDARG00000009693	llgl1	207	27	-2,71	2,49E-04
ENSDARG00000016551	iqsec1b	1087	153	-2,71	6,22E-06
ENSDARG00000076697	zmp:0000001168	86	9	-2,71	1,40E-02
ENSDARG00000058585	chst2b	55	6	-2,72	5,16E-03
ENSDARG00000055075	svila	385	44	-2,72	2,00E-03
ENSDARG00000062864	gk5	176	22	-2,72	8,47E-04
ENSDARG00000025299	tspan9a	185	21	-2,73	3,00E-03
ENSDARG00000069946	itga6b	4400	521	-2,74	1,24E-03
ENSDARG00000025108	magixa	138	15	-2,74	3,70E-03
ENSDARG00000078733	cnnm2b	140	15	-2,75	2,29E-03
ENSDARG00000105040	fam69c	650	77	-2,75	8,58E-04
ENSDARG00000040799	sst1.1	5248275	438229	-2,76	1,26E-02
ENSDARG00000106473	CABZ01076968.1	174	22	-2,76	1,99E-04
ENSDARG00000038981	zgc:153615	402	47	-2,76	8,06E-04
ENSDARG00000026979	krt1-c5	23	2	-2,77	3,88E-02
ENSDARG00000021494	hnf4a	316	40	-2,77	1,07E-04
ENSDARG00000031777	pparaa	139	15	-2,78	1,67E-03
ENSDARG00000004377	ppp1r13ba	1658	175	-2,78	2,98E-03
ENSDARG00000102279	zmiz1b	499	54	-2,79	2,39E-03
ENSDARG00000090164	si:ch73-362m14.2	225	21	-2,79	9,13E-03
ENSDARG00000055843	cdh10a	1800	169	-2,79	2,36E-02
ENSDARG00000056922	ltbp1	168	18	-2,79	2,50E-03
ENSDARG00000043843	akap7	45	4	-2,80	1,05E-02
ENSDARG00000018817	bdnf	1758	189	-2,80	1,87E-03
ENSDARG00000010712	npffr1l3	1488	180	-2,80	3,29E-04
ENSDARG00000006832	galnt9	100	10	-2,80	5,86E-03
ENSDARG00000015476	iqch	39	2	-2,80	4,16E-02
ENSDARG00000020080	ek1	1744	189	-2,80	1,34E-03

ENSDARG00000095458	si:dkey-240h12.3	328	36	-2,81	1,16E-03
ENSDARG00000027602	lrfn1	123	13	-2,81	1,53E-03
ENSDARG00000111945	CABZ01069040.1	245	21	-2,82	4,62E-03
ENSDARG00000102335	ano1	218	18	-2,82	1,30E-02
ENSDARG00000075793	prex1	195	22	-2,83	6,34E-04
ENSDARG00000075159	meltf	156	7	-2,83	1,73E-02
ENSDARG00000101095	cpne4a	3891	403	-2,83	1,99E-03
ENSDARG00000073883	clstn3	2585	305	-2,84	1,63E-04
ENSDARG00000069692	col7a1l	117	12	-2,84	2,08E-03
ENSDARG00000036036	mdka	265	26	-2,84	2,93E-03
ENSDARG00000101054	syn2b	40	2	-2,84	3,21E-02
ENSDARG00000102336	FQ377903.2	34	0	-2,84	8,57E-03
ENSDARG00000111701	LT631684.2	278	33	-2,85	1,13E-04
ENSDARG00000062687	kcnh7	275	30	-2,85	6,67E-04
ENSDARG00000103295	cyp3a65	4761	479	-2,85	2,28E-03
ENSDARG00000096603	bmb	47	4	-2,86	1,37E-03
ENSDARG00000115070	si:dkeyp-74b6.2	156	15	-2,87	1,17E-03
ENSDARG00000104315	fhl3b	84	6	-2,87	7,47E-03
ENSDARG00000074004	stac	37	2	-2,88	3,36E-02
ENSDARG00000069698	lrguk	1247	133	-2,88	8,67E-04
ENSDARG00000055874	cpe	38713	4901	-2,89	1,01E-07
ENSDARG00000103576	LO016987.1	300	29	-2,89	2,31E-03
ENSDARG00000054786	faah2b	51	5	-2,89	4,11E-03
ENSDARG00000023537	ahr1b	287	32	-2,89	2,90E-04
ENSDARG00000100019	arhgap11a	40	3	-2,89	1,74E-02
ENSDARG00000073963	si:ch211-258f14.2	78	4	-2,91	7,21E-03
ENSDARG00000100739	CABZ01052573.1	23	1	-2,92	2,53E-02
ENSDARG00000059680	fscn1a	518	58	-2,92	1,07E-04
ENSDARG00000069134	vwc2l	39	3	-2,93	5,86E-03
ENSDARG00000036968	si:ch1073-416d2.3	1069	103	-2,93	1,12E-03
ENSDARG00000003991	fhl2b	104	5	-2,93	2,58E-02
ENSDARG00000002937	meis1a	6382	720	-2,94	4,75E-05
ENSDARG00000089292	adgrl1a	206	23	-2,94	1,53E-04
ENSDARG00000079166	ace	3673	335	-2,96	1,61E-03
ENSDARG00000004451	tnfrsfa	131	12	-2,97	7,92E-04
ENSDARG00000027903	adamtsl3	188	17	-3,00	8,47E-04
ENSDARG00000042545	sema3ga	147	16	-3,00	5,87E-05
ENSDARG00000098810	zgc:194678	756	80	-3,00	7,67E-05
ENSDARG00000031763	smad6b	348	37	-3,00	4,89E-05
ENSDARG00000104054	mlxipl	3048	320	-3,00	9,27E-05
ENSDARG00000113674	CABZ01058222.1	457	41	-3,01	1,41E-03
ENSDARG00000058115	fgfr2	269	25	-3,01	6,14E-04
ENSDARG00000074082	CU467828.1	56	4	-3,02	2,14E-02
ENSDARG00000059951	pld6	22	1	-3,03	3,25E-02
ENSDARG00000073752	CACNA2D1	55	3	-3,03	1,58E-02
ENSDARG00000077134	gpr158a	1351	137	-3,03	1,07E-04
ENSDARG00000086665	si:dkey-175g6.2	716	46	-3,04	2,78E-03
ENSDARG00000056633	fgf13b	123	10	-3,05	7,63E-04
ENSDARG00000017579	si:ch211-195b15.7	23	2	-3,06	1,16E-02
ENSDARG00000052405	pak6b	134	11	-3,06	2,83E-03
ENSDARG00000099101	gch2	1019	49	-3,07	2,78E-03
ENSDARG00000076404	mctp1a	94	8	-3,07	1,72E-03
ENSDARG00000078335	amot	154	9	-3,08	6,95E-03
ENSDARG00000013522	pck1	217	4	-3,09	7,71E-03
ENSDARG00000032630	neb	48	2	-3,09	1,78E-02
ENSDARG00000090889	si:ch211-132p1.3	650	35	-3,10	5,80E-03
ENSDARG00000102340	ptn	15430	1455	-3,10	1,29E-04
ENSDARG00000103359	slitrk5a	49	2	-3,10	4,32E-03
ENSDARG00000078322	col12a1a	144	9	-3,11	6,37E-03
ENSDARG00000040466	vil1	537	49	-3,13	2,29E-04
ENSDARG00000091061	slc38a3b	612	51	-3,13	4,19E-04
ENSDARG00000058996	jam2a	29	1	-3,16	2,08E-02
ENSDARG00000029431	si:ch211-147k10.5	31	1	-3,16	1,11E-02
ENSDARG00000079907	ptger4c	147	1	-3,17	2,58E-03
ENSDARG00000089640	si:dkey-117n7.5	39	2	-3,17	2,49E-03
ENSDARG00000002533	adora2b	100	5	-3,17	2,73E-03
ENSDARG00000086746	prodha	2376	213	-3,17	9,63E-05
ENSDARG00000036414	si:ch73-335l21.1	514	46	-3,18	1,07E-04
ENSDARG00000116442	FAM46A	987	84	-3,19	1,86E-04
ENSDARG00000044808	slc4a4b	214	16	-3,19	1,10E-03
ENSDARG00000100772	si:ch211-154c21.1	26	1	-3,19	1,01E-02
ENSDARG00000088593	chst1	115	9	-3,20	3,80E-04
ENSDARG00000036483	c1ql3b	48	1	-3,20	4,58E-03

ENSDARG00000019541	gpt2l	9696	853	-3,21	5,87E-05
ENSDARG00000087646	runx1	55	1	-3,21	6,23E-03
ENSDARG00000045878	nudt4b	277	23	-3,21	2,19E-04
ENSDARG00000015110	slc18a2	1857	151	-3,22	2,17E-04
ENSDARG00000090624	ADGRL3	789	59	-3,23	6,53E-04
ENSDARG00000075956	faxca	348	29	-3,24	1,34E-04
ENSDARG00000098925	prdm1b	66	4	-3,24	1,48E-03
ENSDARG00000069254	chrm4a	2516	181	-3,26	9,30E-04
ENSDARG00000099729	pcdh10a	10502	886	-3,27	5,70E-05
ENSDARG00000115315	CU633857.1	23	1	-3,28	1,21E-02
ENSDARG00000007436	avpr2aa	2385	165	-3,29	7,67E-04
ENSDARG00000103747	cav1	771	60	-3,29	1,34E-04
ENSDARG00000013721	g6pca.2	90	6	-3,31	6,82E-04
ENSDARG00000099979	tgfbr3	125	8	-3,32	1,48E-03
ENSDARG00000024865	dscama	4213	337	-3,32	6,58E-05
ENSDARG00000114823	LO018148.1	42	1	-3,32	4,75E-03
ENSDARG00000067781	urp2	822	42	-3,33	3,76E-03
ENSDARG00000053487	osbp2	543	45	-3,34	6,76E-06
ENSDARG00000002600	pcsk1	38882	3275	-3,35	4,48E-06
ENSDARG00000091073	alkal2a	83	4	-3,36	3,06E-03
ENSDARG00000011171	si:ch211-12m10.1	213	14	-3,37	6,63E-04
ENSDARG00000109715	sncgb	393	27	-3,37	2,29E-04
ENSDARG00000089107	mlxipl	1604	118	-3,38	9,98E-05
ENSDARG00000090883	gabra3	365	27	-3,38	1,14E-04
ENSDARG00000042646	zgc:77784	202	13	-3,39	5,99E-04
ENSDARG00000105281	p2ry1	66	1	-3,40	1,10E-02
ENSDARG00000112637	ANOS1	122	7	-3,40	9,71E-04
ENSDARG00000029493	f9b	27	0	-3,42	1,22E-02
ENSDARG00000021866	upk1a	808	52	-3,44	3,28E-04
ENSDARG00000074329	arvcfa	262	10	-3,47	2,31E-03
ENSDARG00000102180	shroom3	500	36	-3,49	2,24E-05
ENSDARG00000023316	sim1a	1242	77	-3,52	1,14E-04
ENSDARG00000043323	lnx1	111	7	-3,54	2,23E-04
ENSDARG00000074443	gas7a	336	17	-3,57	4,52E-04
ENSDARG00000062447	hecw2a	119	6	-3,58	3,38E-04
ENSDARG00000004227	pde3a	897	54	-3,59	9,50E-05
ENSDARG00000062277	ppp2r2cb	44	1	-3,62	6,70E-03
ENSDARG00000079581	nyap2a	105	5	-3,65	5,93E-04
ENSDARG00000055123	kcnk2a	72	3	-3,69	2,82E-03
ENSDARG00000055373	sema3fb	33	0	-3,74	8,16E-03
ENSDARG00000042816	mmp9	502	24	-3,74	1,22E-04
ENSDARG00000056080	si:dkey-191g9.5	113	3	-3,74	1,84E-03
ENSDARG00000077367	ntng2a	311	13	-3,78	4,73E-04
ENSDARG00000077761	nlgn4b	183	7	-3,79	3,59E-04
ENSDARG00000032482	si:dkey-40c11.2	185	9	-3,80	1,29E-04
ENSDARG00000109233	bmp10l	32	0	-3,84	3,56E-03
ENSDARG00000101641	trpm2	845	34	-3,85	2,34E-04
ENSDARG00000020289	pif1	117	2	-3,87	1,00E-03
ENSDARG00000060025	nsmfa	44	1	-3,88	2,05E-03
ENSDARG00000093453	ahdc1	43	1	-3,89	1,37E-02
ENSDARG00000099446	slit1b	213	1	-3,89	3,01E-03
ENSDARG00000077818	nrg2a	24	0	-3,92	2,57E-03
ENSDARG00000075352	brinp3b	41	0	-3,97	2,00E-02
ENSDARG00000087241	ucn3l	2686	81	-4,00	1,50E-04
ENSDARG00000014420	elavl3	198	5	-4,07	4,97E-04
ENSDARG00000018066	ptchd1	116	4	-4,09	4,41E-05
ENSDARG00000098161	cntn4	314	4	-4,10	4,88E-04
ENSDARG00000055459	dlgap3	91	3	-4,15	6,20E-04
ENSDARG00000090686	slc35f2l	59	1	-4,15	9,30E-04
ENSDARG00000019451	pcsk2	132972	4229	-4,31	2,36E-05
ENSDARG00000035198	gcnt4a	199	2	-4,34	3,42E-04
ENSDARG00000045265	gsg1l2b	565	17	-4,40	1,35E-05
ENSDARG00000012968	rhoub	131	2	-4,40	2,05E-04
ENSDARG00000098240	meis2a	474	2	-4,42	1,86E-04
ENSDARG00000095788	ghrhrl	154	2	-4,49	6,52E-04
ENSDARG00000040261	runx2a	79	0	-4,70	8,47E-04
ENSDARG00000076351	brinp3a.1	373	8	-4,72	4,41E-05
ENSDARG00000011797	fam46bb	317	4	-5,12	4,75E-05
ENSDARG00000105113	galr2b	333	0	-5,14	4,73E-04
ENSDARG00000009014	col11a1b	529	4	-5,46	2,08E-05
ENSDARG00000013607	sema3gb	433	2	-5,85	2,08E-05
