# Supplementary material for: A δ-cell subpopulation with a pro-β-cell identity contributes to efficient age-independent recovery in a zebrafish model of diabetes
Source: eLife. 2022 Jan 21;11:e67576. doi: 10.7554/eLife.67576 (PMC8820734; doi:10.7554/eLife.67576)
Supplement: Figure 5—source data 2. [file elife-67576-fig5-data2.pdf]

Figure 5-Source Data 2
Overlap between genes overexpressed in GFPhigh and beta-cell markers
Overlap between genes overexpressed in GFPlow and sst2 delta-cell markers

184 "sst2 delta cell markers" from Tarifeño-Saldivia 2017	142 common genes between "GFPlow" and "sst2 delta markers"	36 common genes between "GFPhigh" and "beta cell markers"
sst2	mapk14a	wif1
sst1.2	lamb2	srpx
ghrl	ppp1caa	fgfr1b
kctd12.2	slc26a3.2	g6pcb
map2k6	pfas	kcnj2a
zgc:195023	stom	prkcbb
aebp1	adgb	grapb
amd1	npy8br	dpp6b
ppp1caa	tbx2b	grin2bb
anxa11a	nrcama	ppdpfb
mapk14a	hmx4	plxnb3
pitpnaa	itga11b	nkx3.2
oaz2a	ank2a	runx2a
ckbb	spegb	ca7
ptger1b	si:ch73-265h17.1	ca12
slc7a7	fgfr1a	rrad
cldni	sema3fa	tmtc1
map3k15	col2a1b	zgc:154058
nmba	ak5	arvcfa
carhsp1	map3k15	prkdc
stmn1b	sst2	klb
kcnk3b	arl3l2	iqsec2b
map1ab	ddc	nlgn4b
slc26a3.2	mmp2	CSMD3 (1 of many)
marcksl1b	sp7	ace
si:ch211-195b15.8	atp1a1b	man2c1
atp1a1b	si:ch211-254n4.3	tmem163a
kl	mdkb	ucn3l
fabp6	slmapb	si:ch211-242f23.3
gulp1b	adgrv1	cntn4
olfm2a	cdh11	gabrb4
serpini1	map1ab	pcdh10a
LO018309.1	adra1d	zgc:158260
mdkb	fgf12a	CABZ01085139.1
TMEM179 (1 of many)	prkg1b	LO017650.1
HHEX	rxfp2a	hcn1
CABZ01079192.1	nrbp2a	
lamc2	sst1.2	
stxbp6	zgc:100906	
cpamd8	stmn1b	
cdx1b	ncf1	
slmapb	serpini1	
mrap2a	marcksl1b	
tbx2b	si:dkeyp-92c9.2	
spegb	anks4b	
srd5a2b	plxnb2b	
si:dkey-206p8.1	casz1	
nacc2	rab30	
edil3a	pald1b	
sema3fa	pitpnaa	
hopx	GALNTL6	
nrcama	adarb1a	
ncf1	slc8a1b	
nrbp2a	amd1	
fgfr1a	loxl2a	
phkg2	sall4	
slc7a14a	fabp6	
si:dkey-85k7.7	myo7ab	

cdh11	tmcc3
maptb	cntn1b
lamb2	kcnk3b
si:dkey-85k7.7	zgc:195023
zgc:100906	carhsp1
si:dkey-280e21.3	si:dkey-166d12.2
loxl2a	kctd12.2
adra1d	irs1
prkg1b	phkg2
tshz3a	ghrl
srpx2	cldni
rxfp2a	slc7a7
napbb	pknox2
pygma	pygma
pfas	celsr3
yjefn3	ca6
stom	si:dkey-280e21.3
npy8br	cpamd8
DUT	LO018309.1
ddc	stoml3b
nhsb	plppr5a
tmcc3	gulp1b
ak5	cavin1b
wnk4b	CU019662.1
rab30	si:ch211-216b21.2
ank2a	pear1
ca6	trpc5b
kcnt1	yjefn3
arl3l2	bmp7b
gpr4	syngap1a
irs1	nmba
ifitm5	lamc2
mertka	napbb
mmp2	slc26a4
syt10	si:ch211-168d1.3
sgcd	hpcal4
chgb	lrrc39
CABZ01088484.1	tgfb2l
znf1003	cntnap5l
plppr5a	mertka
tub	mrap2a
si:dkey-166d12.2	chgb
myo7ab	anxa11a
nr0b1	lypd6b
adcyap1r1a	pdgfd
adgrv1	olfm2a
bmp7b	lmf2a
CU019662.1	pacs2
hmx4	kcnt1
slc8a1b	si:ch211-152p11.4
lrrc39	kl
prss23	nhsb
lmf2a	bahcc1b
itga11b	reep6
si:ch211-152p11.4	tshz3a
hpcal4	maptb
ttc6	FAT3 (1 of many)
pear1	si:dkey-118k5.3
GALNTL6	stxbp6
reep6	wnk4b
axdnd1	hopx
cavin1b	TMEM179 (1 of many)
bahcc1b	fam20cb
lrrc7	aebp1
col2a1b	zmat4a
trpc5b	edil3a
si:dkey-118k5.3	artnb

si:ch211-286o17.1
cntnap5l
col14a1a
casz1
FAM135B
anks4b
stoml3b
fgf12a
adarb1a
chrnb4
si:ch211-254n4.3
syne1b
celsr3
artnb
casp8l1
evla
si:ch211-216b21.2
lrrc4.1
pkd1l2b
si:ch211-186e20.7
pacs2
bmp4
plxnb2b
slc26a4
ino80e
lypd6b
pdgfd
tox2
alx3
ppp4r1l
si:ch211-168d1.3
ano2
fam20cb
cep152
arrb1
cntn1b
sall4
capn10
tgfb2l
fkbp10a
pknox2
adgb
zgc:158328
si:ch211-150i13.1
col4a4
nadl1.2
ntn2
FAT3 (1 of many)
sp7
si:dkeyp-106c3.1
adrb2a
pald1b
syngap1a
zmat4a
si:dkeyp-92c9.2
sdk1b
CABZ01043499.1
si:ch73-265h17.1
RASGRP2

si:ch211-195b15.8
si:dkey-206p8.1
sgcd
fkbp10a
map2k6
ppp4r1l
tox2
si:ch211-286o17.1
si:dkey-85k7.7
ano2
CABZ01079192.1
chrnb4
ttc6
CABZ01088484.1
ptger1b
ifitm5
cdx1b
