# Supplementary material for: A δ-cell subpopulation with a pro-β-cell identity contributes to efficient age-independent recovery in a zebrafish model of diabetes
Source: eLife. 2022 Jan 21;11:e67576. doi: 10.7554/eLife.67576 (PMC8820734; doi:10.7554/eLife.67576)
Supplement: Figure 5—source data 3. [file elife-67576-fig5-data3.pdf]

Figure 5-Source Data 3. sst1.1 δ-cell (GFPhigh) and updated β-cell markers

Gene expression enrichment >4-fold

sst1.1 delta cell markers enriched >4x versus
alpha, beta and sst2 delta cells (from Tarifeño-Saldivia 2017)

sst1.1
FO704772.2
mfge8b
cyp3a65
itga6b
gfra1a
cpne4a
ca4a
ace
ucn3l
c9
chrm4a
avpr2aa
bdnf
cdh10a
mst1rb
ppp1r13ba
mtbl
lrguk
boc
atp2a2a
cacna1bb
iqsec1b
insl5b
lrrk1
urp2
dlc
mtus1b
dnajc6
upk1a
nrp1b
ADGRL3
sez6b
pcsk6
arhgap32b
adra2da
slc38a3b
slc4a7
xylt1
l1cama
ephb2b
syt7a
dgat2
slit1a
igf1ra
sik3
mc4r
col11a1b
si:ch211-153b23.5
slc34a1a
si:ch211-207i1.2
rxfp3.3a1
si:ch211-264f5.6
arhgap42b
sema3gb
vav3b
gabra3
cdk15
KCNIP4
si:dkey-240h12.3
comtb
klf3
faxca
kif20ba
GSK3B (1 of many)
brinp3a.1
ntng2a
asip2b
nudt4b
mdka
galr2b
shank2

updated beta cell markers enriched >4x versus
alpha, sst1.1 delta and sst2 delta cells

ins
nkx6.2
fstl1a
esr1
zgc:158654
gcgra
abhd15a
ngfra
otc
srpx
fstl1b
rab3c
cacng3b
pnocb
si:ch211-246m6.5
ndufa4l2a
si:ch211-236d3.4
chrm5a
rasd4
si:ch211-246m6.5
ppox
colgalt2
clstn2
raly
efnb3b
adra2b
si:ch211-260e23.9
aplnra
iqsec2b
zgc:91818
tmem196b
zgc:162160
itga9
rnd1b
rasa4
twist1b
cabp5a
nostrin
rhbdf1b
elf3
eif4ebp3l
grip2b
olfml2ba
ptprt
cacng5b
tagln3b
thbs3a
angptl1a
AL935128.1
panx1b
cart3
sdsl
CABZ01072077.1
CABZ01020840.1
elfn1b
gypc
mthfd1l
gstt1b
slc6a15
rorcb

gpbar1
CABZ01063170.1
cpeb3
vstm2l
cpne7
gas2l3
ptprsa
dhrs7ca
zgc:77784
slc4a4b
ano1
elavl3
cacnb2b
slit1b
gk5
dgkzb
ptger4c
ano5a
VSTM2A
slc8a4a
mbd5
ghrhrl
si:ch1073-391i24.1
ltbp1
kif26aa
phf24
amot
col7a1l
atxn7l1
sox9a
lrfn1
tnfrsfa
si:dkey-63b1.1
alkal2a
nr6a1a
larp1
pif1
myl7
hecw2a
lnx1
adora2b
adam10b
st8sia3
ptch1
hey2
crlf1a
plxna2
kif1c
hs3st2
fam20ca
si:dkey-191g9.5
scospondin
nyap2a
avpr2ab
CABZ01075125.1
b3gnt7
omgb
dlgap3
lrp6
BACE1
zmp:0000001168
col11a1a
prdm1b
znf710a
si:ch73-72b7.1
si:ch211-129o18.4
kcnk2a
p2ry1
BX548011.1
runx1
ptch2
crybb1l2
atn1
cplx1
fam167b
stard9
ntrk1
CU467828.1
CR388166.1
si:ch211-119o8.4
