# Supplementary material for: A δ-cell subpopulation with a pro-β-cell identity contributes to efficient age-independent recovery in a zebrafish model of diabetes
Source: eLife. 2022 Jan 21;11:e67576. doi: 10.7554/eLife.67576 (PMC8820734; doi:10.7554/eLife.67576)
Supplement: Figure 5—source data 4. [file elife-67576-fig5-data4.pdf]

Figure 5-Source Data 4. Biological processes and pathways overrepresented in GFPlow

GO identified by WebGestalt (FDR<0.25)
among genes overexpressed in sst1.1:GFPlow (versus sst1.1:GFPhigh delta cells) (DESeq FC>2x, Padj<0.05)

Biological Process (non redundant)

geneSet	description	enrichmentRatio	pValue	FDR	userId
GO:0030182	neuron differentiation	1,753845509	5,91E-05	0,016775464	mapk14a;isl1;casp9;tbx2b;skib;sema3fa;slc26a2;twf1b;glra1;eya4;arl3l2;mdkb;cdh11;fzd3a;tnc;map1ab;ntn1b;zgc:110006;stmn1b;slit3;rab13;ctdsp2;pls3;lfng;pitpnaa;atg4da;cdk5r1b;id4;hgfa;sema4e;kctd12.2;prox1a;kif17;id2a;alcamb;sema5a;ngfa;wnt9a;wnt7bb;wnt6b;cerkl;rbpms2b;sema4c;dscaml1;slitrk6;wnt5b;vegfaa;ndrg4;efnb3a;lrrc4c
GO:0032989	cellular component morphogenesis	1,731943829	3,08E-04	0,043695496	mapk14a;isl1;casp9;sema3fa;arl3l2;flncb;parvb;rhoj;rhoca;cdh11;fzd3a;tnc;map1ab;ntn1b;cdc42se1;rilpl2;adgrg1;slit3;pls3;pitpnaa;parvaa;sema4e;kctd12.2;prox1a;alcamb;sema5a;ngfa;rnd2;lrrc39;col6a1;rbpms2b;sema4c;rac3a;dscaml1;slitrk6;wnt5b;rbm24a;vegfaa;wtip;cap2;efnb3a;lrrc4c
GO:0051093	negative regulation of developmental process	2,415295815	5,94E-04	0,0561947	skib;fgfr1a;sema3fa;sirt2;tnc;slit3;lfng;loxl2a;id4;irf7;sema4e;id2a;her9;sema5a;yjefn3;rspo3;sema4c
GO:0040011	locomotion	1,561697294	0,001533786	0,07380697	tbx2b;cass4;srgap1a;sema3fa;sst2;arl3l2;ptges;rhoca;cdh11;fzd3a;tnc;ntn1b;hmgb1b;podxl;sst1.2;rab13;arpc5lb;znf703;pls3;socs1a;ptk2bb;jagn1a;cftr;loxl2a;anxa3b;hgfa;sema4e;prox1a;ackr3b;alcamb;sema5a;rxfp3.3b;srgap3;elp6;rnd2;cxcl20;cxcr3.1;rbpms2b;sema4c;rac3a;ccl20b;dscaml1;foxj1a;wnt5b;vegfaa;efnb3a
GO:0045595	regulation of cell differentiation	1,734058534	0,00155598	0,07380697	mapk14a;casp9;skib;ndrg1b;fgfr1a;sema3fa;sirt2;twf1b;glra1;arl3l2;mdkb;tnc;ntn1b;adgrg1;zgc:110006;slit3;atoh8;lfng;jun;loxl2a;id4;irf7;sema4e;kctd12.2;id2a;her9;sema5a;ngfa;rbpms2b;sema4c;rbm24a;lrrc4c
GO:0120036	plasma membrane bounded cell projection organization	1,568697282	0,00157548	0,07380697	mapk14a;isl1;casp9;ttc9c;ehd3;aif1l;sema3fa;sirt2;twf1b;ak5;arl3l2;flncb;cdh11;fzd3a;tnc;map1ab;ntn1b;rilpl2;zgc:110006;stmn1b;slit3;rab13;znf703;hs3st1l2;pls3;dnai1.2;pitpnaa;sema4e;kctd12.2;alcamb;sema5a;ngfa;dzip1l;tmem67;rbpms2b;sema4c;cep162;dscaml1;slitrk6;foxj1a;eps8l3a;vegfaa;wtip;efnb3a;lrrc4c
GO:0009719	response to endogenous stimulus	1,70101626	0,001819186	0,07380697	thraa;crhr1;rxrgb;skib;fgfr1a;glra1;sesn1;dynlrb1;rxfp2a;esr2b;rab13;paqr7b;sfrp1a;znf703;oprd1b;socs1a;oprm1;jun;anxa3b;agtr1b;prlrb;irs1;dusp22a;ngfa;bmp7b;ar;chrm3a;inpp5kb;kl;fgf20b;paqr8;igfbp3;bambib
GO:0051241	negative regulation of multicellular organismal process	2,217320748	0,002139146	0,075939694	fgfr1a;sema3fa;sirt2;tnc;slit3;lfng;loxl2a;id4;irf7;sema4e;id2a;her9;sema5a;yjefn3;rspo3;sema4c
GO:0021675	nerve development	3,145501527	0,003271022	0,100946806	tuba1a;fgfr1a;tnc;adgrg1;klf7b;alcamb;ngfa;chn1
GO:0022603	regulation of anatomical structure morphogenesis	1,737485527	0,004055252	0,100946806	mapk14a;casp9;fgfr1a;sema3fa;sirt2;arl3l2;rhoj;rhoca;tnc;ntn1b;cdc42se1;slit3;ptk2bb;sema4e;kctd12.2;id2a;sema5a;yjefn3;rspo3;rnd2;rbpms2b;sema4c;rbm24a;vegfaa;wtip;lrrc4c
GO:0051674	localization of cell	1,555614254	0,004139596	0,100946806	tbx2b;cass4;srgap1a;sema3fa;sst2;ptges;rhoca;tnc;hmgb1b;podxl;sst1.2;rab13;arpc5lb;znf703;socs1a;ptk2bb;jagn1a;cftr;loxl2a;anxa3b;hgfa;sema4e;prox1a;ackr3b;alcamb;sema5a;rxfp3.3b;srgap3;elp6;rnd2;cxcl20;cxcr3.1;sema4c;rac3a;ccl20b;foxj1a;wnt5b;vegfaa
GO:0032879	regulation of localization	1,523494283	0,004265358	0,100946806	stxbp5a;srgap1a;cldnb;sema3fa;sst2;rab3c;panx1b;arl3l2;rhoca;hmgb1b;cacng2a;sst1.2;unc119a;znf703;socs1a;ptk2bb;cftr;anxa3b;sema4e;prox1a;kcnd3;sema5a;srgap3;elp6;yjefn3;kcnj19b;kcng4a;plcb3;rnd2;dzip1l;cxcr3.1;inpp5kb;kcna4;cerkl;rbpms2b;sema4c;wnk4b;rab3db;foxj1a;wnt5b;vegfaa

GO:0071310	cellular response to organic substance	1,485558514	0,005984187	0,130731462	thraa;mapk14a;crhr1;rxrgb;skib;fgfr1a;glra1;sesn1;dynlrb1;rxfp2a;esr2b;rab13;paqr7b;sfrp1a;znf703;oprd1b;socs1a;oprm1;jagn1a;cftr;jun;ube2w;agtr1b;prlrb;il12rb2;irs1;dusp22a;ackr3b;ngfa;rxfp3.3b;bmp7b;ar;chrm3a;cxcl20;cxcr3.1;inpp5kb;kl;fgf20b;ccl20b;igfbp3;bambib;vegfaa
GO:0051128	regulation of cellular component organization	1,449177489	0,009097203	0,184543266	mapk14a;rassf7b;casp9;rassf1;sema3fa;twf1b;lrrc4bb;arl3l2;rhoj;rhoca;tnc;ntn1b;cdc42se1;si:ch211-106h11.3;gmfb;zgc:110006;stmn1b;slit3;unc119a;arpc5lb;znf703;ptk2bb;sema4e;kctd12.2;sema5a;clstn2;arhgap17b;lnpa;rnd2;tmem67;inpp5kb;cerkl;rbpms2b;sema4c;rac3a;igfbp3;ccnd1;eps8l3a;rbm24a;vegfaa;wtip;lrrc4c
GO:2000026	regulation of multicellular organismal development	1,478869069	0,01029116	0,187158236	mapk14a;casp9;ndrg1b;fgfr1a;sema3fa;sirt2;twf1b;glra1;lrrc4bb;arl3l2;mdkb;tnc;ntn1b;adgrg1;zgc:110006;slit3;atoh8;lfng;ptk2bb;loxl2a;id4;irf7;sema4e;kctd12.2;id2a;her9;sema5a;ngfa;clstn2;yjefn3;rspo3;rbpms2b;sema4c;ccnd1;rbm24a;vegfaa;lrrc4c
GO:0009887	animal organ morphogenesis	1,451250704	0,010544126	0,187158236	thraa;tbx2b;ttc9c;skib;wls;fgfr1a;slc26a2;eya4;veph1;sfrp1a;znf703;toporsa;atoh8;pls3;socs1a;adarb1a;cdk5r1b;fras1;ncs1a;kif17;id2a;edaradd;wnt9a;plcb3;rit1;lrrc39;col6a1;cerkl;fgf20b;reep6;fam20cb;igfbp3;pbx1a;ccnd1;foxj1a;wnt5b;rbm24a;vegfaa;wtip;ndrg4
GO:1902531	regulation of intracellular signal transduction	1,493091959	0,011884115	0,198534626	map4k2;epha7;fgfr1a;ror1;pdia2;sesn1;rcan2;epha2b;mcf2a;epha6;cdc42se1;hmgb1b;paqr7b;socs1a;traf4b;vav2;fgd6;si:dkey-166d12.2;tp53bp2b;dusp22a;bmp7b;mertka;mrap2a;f2rl1.2;inpp5kb;arhgef19;cerkl;tiam2a;ccl20b;si:ch211-195b15.8;map2k6;chn1;eps8l3a;wtip
GO:0007267	cell-cell signaling	1,453924999	0,015763888	0,215806131	stx3a;stx2a;chrna2a;wls;glra1;panx1b;fzd3a;grm3;cacng2a;chrne;sfrp1a;znf703;jun;id4;zgc:162025;ngfa;syn1;clstn2;apba2b;sv2bb;wnt9a;htr5ab;grik1a;rspo3;chrm3a;wnt7bb;wnt6b;mertka;pdyn;gabra2a;bambib;chrnb4;wnt5b;apba1a;notum1b
GO:0008283	cell proliferation	1,591253714	0,015899646	0,215806131	tbx2b;wls;ndrg1b;fgfr1a;sirt2;pdf;tnc;s100a10b;adgrg1;ing4;npr3;ptk2bb;loxl2a;id2a;dusp22a;tacc1;areg;usp13;lmo2;ccnd1;vegfaa;ndrg4;tnk2b;cdx1b
GO:0042391	regulation of membrane potential	2,167573168	0,016083647	0,215806131	slc26a3.2;chrna2a;slc26a3.1;slc26a2;glra1;slc26a5;slc26a1;chrne;gabra2a;chrnb4
GO:0040007	growth	1,600257721	0,016950816	0,215806131	mapk14a;isl1;casp9;fgfr1a;sema3fa;arl3l2;cldnc;tnc;ntn1b;si:ch211-106h11.3;slit3;npr3;lfng;sema4e;sema5a;mrap2a;col6a1;tmem67;sema4c;lmo2;igfbp3;wnt5b;ndrg4
GO:0048568	embryonic organ development	1,484756918	0,017076757	0,215806131	tbx2b;ttc9c;skib;wls;slc26a2;eya4;veph1;sfrp1a;etnppl;znf703;atoh8;pls3;lfng;socs1a;adarb1a;fras1;ncs1a;cavin1b;edaradd;wnt9a;plcb3;rit1;znf219;fgf20b;lmo2;igfbp3;foxj1a;wnt5b;rbm24a;wtip;ndrg4
GO:0035270	endocrine system development	2,140135533	0,017477257	0,215806131	thraa;isl1;tbx2b;cdx4;insm1b;fhl1b;glis3;gcga;wnt5b;vegfaa

KEGG pathways

geneSet	description	enrichmentRatio	pValue	FDR	userId
dre04514	Cell adhesion molecules (CAMs)	2,507829031	2,56E-04	0,041440345	itgav;nrcama;nectin3b;cldnb;lrrc4bb;cldnc;cldn15la;cldn11a;negr1;cldn11b;itga9;cldn5a;cd99;sdc4;zmp:0000001082;si:ch211-286o17.1;cldn15a;lrrc4c
dre04310	Wnt signaling pathway	2,213258637	6,30E-04	0,050998498	tle3a;tcf7l2;camk2d2;gpc4;fzd3a;siah1;sfrp1a;rac2;jun;rac1l;dvl2;wnt9a;plcb3;wnt7bb;wnt6b;cxxc4;rac3a;bambib;ccnd1;wnt5b
dre04510	Focal adhesion	1,912692649	0,001539694	0,08314346	lamb2;ppp1caa;hrasb;itgav;col2a1b;flncb;parvb;tnc;itga9;itga3a;rac2;jun;parvaa;vav2;hgfa;rac1l;flna;itga1;col6a1;pdgfd;rac3a;ccnd1;vegfaa;pdgfaa
dre04512	ECM-receptor interaction	2,566580046	0,003351413	0,135732231	lamb2;itgav;col2a1b;tnc;itga9;itga3a;sdc4;sv2bb;itga1;col6a1;hspg2
dre04115	p53 signaling pathway	2,45139406	0,007036602	0,227985895	casp9;baxa;sesn1;apaf1;siah1;rprma;perp;bida;igfbp3;ccnd1
