# Supplementary material for: A δ-cell subpopulation with a pro-β-cell identity contributes to efficient age-independent recovery in a zebrafish model of diabetes
Source: eLife. 2022 Jan 21;11:e67576. doi: 10.7554/eLife.67576 (PMC8820734; doi:10.7554/eLife.67576)
Supplement: Figure 5—source data 5. [file elife-67576-fig5-data5.pdf]

Figure 5-Source Data 5. Biological processes and pathways overrepresented in GFPhigh

GO identified by WebGestalt (FDR<0.25)
GO from genes overexpressed in sst1.1:GFPhigh (sst1.1 delta cells) versus sst1.1:GFPlow (DESeq FC>2x, Padj<0.05)

Biological Process (non redudant)

geneSet	description	enrichmentRatio	pValue	FDR	userId
GO:0048585	negative regulation of response to stimulus	2,238675993	6,89E-05	0,009197199	pde3a;wif1;mylipa;sema6dl;sema3gb;lemd3;chico;ek1;ephb2b;sema3ga;kita;ret;sema3fb;sik1;rgs7bpa;cdon;sigirr;sema4ab;rasa3;dkk3b;met;dab2ipb;sik2a;ntrk3b;dusp2;dusp7;numbl
GO:0051336	regulation of hydrolase activity	2,130937538	9,03E-05	0,009197199	llgl1;asap2a;serpinb1l3;arhgap4b;iqsec1b;si:ch211-195b15.7;ek1;crim1;ppp1r14bb;ppp2r5cb;plxnb3;si:dkeyp-19e1.3;si:dkey-191g9.5;tbc1d9;iqgap2;plxna2;ppp2r2cb;rasa3;syngap1b;ppp1r3ca;arhgap32b;dab2ipb;arhgap32a;pcsk1nl;si:ch211-218g4.2;si:dkey-117n7.5;si:dkey-21e13.3;ppp2r2bb;ndel1b
GO:0010648	negative regulation of cell communication	2,332553743	1,30E-04	0,009197199	pde3a;wif1;chga;mylipa;lemd3;chico;ek1;ephb2b;kita;ret;sik1;pfkla;rgs7bpa;cdon;rasa3;dkk3b;met;dab2ipb;sik2a;ntrk3b;dusp2;dusp7;numbl
GO:0023057	negative regulation of signaling	2,332553743	1,30E-04	0,009197199	pde3a;wif1;chga;mylipa;lemd3;chico;ek1;ephb2b;kita;ret;sik1;pfkla;rgs7bpa;cdon;rasa3;dkk3b;met;dab2ipb;sik2a;ntrk3b;dusp2;dusp7;numbl
GO:0071526	semaphorin-plexin signaling pathway	5,368676686	2,44E-04	0,013876777	sema6dl;sema3gb;plxnb3;sema3ga;sema3fb;plxna2;sema4ab
GO:0022610	biological adhesion	2,10091628	7,77E-04	0,036758344	adora2b;ptprfb;dlg1;ctnnb1;ek1;plxnb3;fat3a;itga6a;pkp3a;ret;epdl2;cdh10a;plxna2;ctnnd2a;vcam1b;celsr1a;epdl1;nlgn1;nlgn2b;runx1;cntn4;pcdh10a
GO:0048568	embryonic organ development	1,877190068	0,00116332	0,047197574	atp1a1a.1;gpr22a;gon4l;ncoa2;prkcbb;atp2a2a;crim1;csrnp1a;s1pr2;nkx3.2;pacsin1b;ngs;hdac9b;plxna2;furina;etv5a;hoxc4a;cxxc5a;runx1;scarb2a;polr1a;vdra;numbl;irf6;notch1a;cav1;grk3
GO:0016311	dephosphorylation	2,148884414	0,001343289	0,047686777	pfkfb2b;ptprfb;g6pca.2;g6pcb;ptprdb;ppp1r14bb;ppp2r5cb;ptprga;ppm1nb;ptprna;ca16b;ppp2r2cb;ppp1r3ca;ptprnb;ptprua;dusp2;ppp2r2bb;dusp7;plppr2a
GO:0032879	regulation of localization	1,726067481	0,001693151	0,053428331	chga;sema6dl;arrb2b;sema3gb;arhgap4b;cplx2l;kcnj2a;slc30a2;cacna1ba;mdga2a;pparaa;plxnb3;rhbdf1a;s1pr2;sema3ga;rrad;pparab;sema3fb;letm1;jph1a;kcnj19a;scn1ba;plxna2;pfkla;sema4ab;scn1lab;gck;amot;vegfba;kcng1;notch1a;rbm26
GO:0005975	carbohydrate metabolic process	2,103644742	0,002260426	0,064196101	pfkfb2b;g6pca.2;g6pcb;gys1;slc23a2;aldocb;pfkpa;chst2b;pfkla;ndst2b;hs2st1b;scn1lab;man2a2;gck;ppp1r3ca;klb;man2c1;chst1
GO:0007267	cell-cell signaling	1,688429032	0,004441315	0,114666672	wif1;chga;mylipa;dlg1;ndrg2;bdnf;cplx2l;chico;cacna1ba;nlk2;grin2bb;bcl9;zgc:101731;grid1b;bcl9l;scgn;sv2a;pfkla;scn1lab;gck;chrm4a;dkk3b;met;nlgn1;nlgn2b;gucy1b1;gabrb4;syn2b

GO:0051674	localization of cell	1,663899796	0,005423833	0,12836405	atp1a1a.1;sema6dl;arrb2b;sema3gb;arhgap4b;ek1;stat3;mdga2a; plekhg5a;plxnb3;rhbdf1a;s1pr2;sema3ga;nhsl1b;kita;apln;ret; sema3fb;plxna2;sema4ab;rxfp3.3a1;celsr1a;met;amot;vegfba; nr6a1a;ndel1b;zbtb4
GO:0040011	locomotion	1,577029526	0,006725155	0,146918761	atp1a1a.1;ptprfb;sema6dl;arrb2b;sema3gb;arhgap4b;ek1;stat3; mdga2a;plekhg5a;plxnb3;rhbdf1a;s1pr2;sema3ga;nhsl1b;kita;apln; ret;sema3fb;plxna2;sema4ab;rxfp3.3a1;celsr1a;chrm4a;met;gba; amot;vegfba;cntn4;nr6a1a;ndel1b;zbtb4
GO:0072358	cardiovascular system development	1,725033495	0,008771799	0,17611069	vash2;lemd3;iqsec1b;plekhg5a;crim1;s1pr2;mat2aa;apln;hdac9b; colec12;foxo1b;vcam1b;rasa3;dab2ipb;nlgn1;amot;cxxc5a;runx1; vegfba;foxo1a;mtbl;notch1a
GO:0048598	embryonic morphogenesis	1,594409782	0,009521346	0,17611069	atp1a1a.1;lats1;gpr22a;gon4l;mylipa;stat3;plekhg5a;atp2a2a;crim1; s1pr2;nkx3.2;pacsin1b;apln;ngs;plxna2;cdon;furina;celsr1a;etv5a; hoxc4a;sall1a;cxxc5a;polr1a;vdra;irf6;notch1a;grk3;zbtb4
GO:0060485	mesenchyme development	2,149709956	0,009921729	0,17611069	gpr22a;sema6dl;sema3gb;ek1;stat3;sema3ga;bcl9l;ret;sema3fb; sema4ab;furina;polr1a
GO:0071310	cellular response to organic substance	1,540325648	0,011744623	0,196204287	lemd3;keap1a;ncoa2;bdnf;hsp70.3;smad9;crlf1a;smad6b;pparaa;s i:ch73-335l21.1;kita;pparab;nr4a3;foxo1b;rxfp3.3a1;chrm4a;sall1a; dab2ipb;tet1;brinp3b;avpr1aa;rxrba;ntrk3b;vegfba;vdra;foxo1a; nr6a1a;mapk10;cav1;bmpr1ba
GO:1902531	regulation of intracellular signal transduction	1,593670259	0,013989696	0,220710669	pde3a;arhgef4;lemd3;iqsec1b;kank2;ek1;ephb2b;prkcbb;plekhg5a; arhgef1a;kita;pak6b;ret;eps8l2;sik1;rasa3;met;arfgef2;prex1;iqsec2b; sik2a;ntrk3b;dusp2;sh3rf2;dusp7
GO:0007498	mesoderm development	2,684338343	0,01482677	0,220710669	gpr22a;apln;bcl9l;furina;etv5a;zmiz2;notch1a
GO:0045595	regulation of cell differentiation	1,651900519	0,016229692	0,220710669	adora2b;pde3a;sema6dl;sema3gb;elavl3;bdnf;ek1;stat3;mdga2a; plxnb3;pacsin1b;sema3ga;sema3fb;plxna2;cdon;sema4ab;brinp3b; plekho1b;runx1;notch1a;add1
GO:0051240	positive regulation of multicellular organismal p	1,945443817	0,016320155	0,220710669	pde3a;olfm1a;myl7;stat3;plxnb3;pacsin1b;apln;plxna2;brinp3b; avpr1aa;runx1;vegfba;notch1a
GO:0051094	positive regulation of developmental process	1,976578953	0,018387923	0,237371372	pde3a;olfm1a;stat3;plxnb3;pacsin1b;plxna2;cdon;brinp3b;runx1; vegfba;notch1a;add1
GO:0033993	response to lipid	2,029832646	0,019640816	0,242521376	igf2r;keap1a;pparaa;slc37a4a;pparab;nr4a3;tet1;brinp3b;rxrba; vdra;nr6a1a

KEGG pathways

geneSet	description	enrichmentRatio	pValue	FDR	
dre04514	Cell adhesion molecules (CAMs)	3,404163361	6,33E-07	1,03E-04	cntn2;sdc2;ptprfb;ncam1b;cadm1a;itga6a;cadm1b;si:ch73-61d6.3; cntnap2a;jam2a;nfasca;glg1a;vcam1b;itga6b;cd28l;ntng2a;nlgn1; nlgn2b;nectin1b;cntn1a;ptprfa
dre00051	Fructose and mannose metabolism	3,668051994	0,002556591	0,156903699	pfkfb2b;aldocb;pfkpa;sord;pfkfb4a;pfkla;hk2
dre00052	Galactose metabolism	4,096785344	0,002905624	0,156903699	g6pca.2;g6pcb;pfkpa;pfkla;gck;hk2
dre00500	Starch and sucrose metabolism	3,755386565	0,004571532	0,171692908	g6pca.2;g6pcb;gys1;zgc:66313;gck;hk2
dre01230	Biosynthesis of amino acids	2,56049084	0,005299164	0,171692908	tktb;cbsb;gpt2l;aldocb;aco1;pfkpa;mat2ab;mat2aa;pklr;pfkla
dre00010	Glycolysis / Gluconeogenesis	2,633647721	0,006670297	0,180098029	pck1;g6pca.2;g6pcb;aldocb;pfkpa;pklr;pfkla;gck;hk2
dre00534	Glycosaminoglycan biosynthesis	3,755386565	0,009490054	0,219626956	hs3st2;ndst2b;xylt1;hs2st1b;glceb
