# Supplementary material for: A δ-cell subpopulation with a pro-β-cell identity contributes to efficient age-independent recovery in a zebrafish model of diabetes
Source: eLife. 2022 Jan 21;11:e67576. doi: 10.7554/eLife.67576 (PMC8820734; doi:10.7554/eLife.67576)
Supplement: Figure 6—source data 1. [file elife-67576-fig6-data1.pdf]

Figure 6-Source Data 1

sst1.1GFP^{high} cell number

Adult pancreas (FACS)

CTL	3dpt	20dpt
708	115	56
1014	565	719
1628	373	123
804	399	472
1383	242	708
792	532	337
1078	639	54
938	697	50
933		
704		
788		
