# Supplementary material for: A δ-cell subpopulation with a pro-β-cell identity contributes to efficient age-independent recovery in a zebrafish model of diabetes
Source: eLife. 2022 Jan 21;11:e67576. doi: 10.7554/eLife.67576 (PMC8820734; doi:10.7554/eLife.67576)
Supplement: Figure 6—source data 2. [file elife-67576-fig6-data2.pdf]

Figure 6-Source Data 2. Significant DE genes between sst1.1 δ-cells and 20 dpt bihormonal cells.

DESeq comparison (Padj<0.05)

Ensembl_ID	Gene_Name	Mean_ CTL_sst1.1_delta	Mean_ 20dpt_bihormonal	log2FC	Padj
ENSDARG00000035350	ins	2769	398302	5,76	3,20E-07
ENSDARG00000114192	BX548011.5	4	400	5,10	7,20E-03
ENSDARG00000022569	nkx6.1	0	62	5,07	6,82E-04
ENSDARG00000101481	rbp5	1	76	4,74	2,13E-04
ENSDARG00000094132	igf1	0	43	4,57	9,02E-03
ENSDARG00000015559	fstl1a	11	479	4,53	4,70E-05
ENSDARG00000061240	cbln4	0	304	4,32	1,69E-03
ENSDARG00000024032	coch	1	93	4,24	2,36E-02
ENSDARG00000062538	si:dkey-215k6.1	0	23	4,03	5,64E-03
ENSDARG00000101637	ccnd1	7	181	3,95	6,74E-06
ENSDARG00000007682	ppdpfa	802	16778	3,85	1,06E-03
ENSDARG00000068892	sostdc1a	6	151	3,78	1,99E-02
ENSDARG00000038066	kpna2	2	122	3,69	1,27E-02
ENSDARG00000056590	calca	0	67	3,64	2,35E-02
ENSDARG00000044691	ppp1r3b	28	568	3,63	7,94E-03
ENSDARG00000102483	wu:fb44b02	0	24	3,56	1,51E-02
ENSDARG00000059798	kcnq1	0	23	3,49	4,69E-02
ENSDARG00000039657	fank1	1	28	3,43	2,85E-02
ENSDARG00000087804	tnfrsf11a	1	43	3,35	2,83E-02
ENSDARG00000002172	aplnra	1	41	3,33	2,56E-02
ENSDARG00000035899	lingo1b	1	57	3,33	2,83E-02
ENSDARG00000092057	si:dkey-90l23.1	387	4570	3,21	3,64E-02
ENSDARG00000014910	panx1b	3	48	3,21	2,37E-03
ENSDARG00000043137	cdca8	0	26	3,18	3,06E-02
ENSDARG00000060259	zgc:162160	7	80	3,18	4,29E-04
ENSDARG00000051923	ccnb1	0	24	3,14	4,12E-02
ENSDARG00000034344	pcdh19	22	296	3,10	1,60E-02
ENSDARG00000070000	txnipb	1	111	3,10	4,40E-02
ENSDARG00000099211	si:dkey-220o5.5	0	42	3,04	3,75E-02
ENSDARG00000004713	mad2l1	1	32	2,99	4,89E-02
ENSDARG00000059956	ptk6b	6	89	2,97	2,46E-02
ENSDARG00000006044	ust	1	35	2,93	2,92E-02
ENSDARG00000057714	cmah	97	920	2,93	1,25E-03
ENSDARG00000102687	CABZ01085139.1	535	5046	2,88	2,21E-02
ENSDARG00000104501	FP236812.1	1	34	2,87	3,88E-02
ENSDARG00000071410	cks2	2	28	2,82	2,78E-02
ENSDARG00000012071	angptl1a	26	489	2,78	4,25E-02
ENSDARG00000074310	rbfox3b	18	192	2,73	2,36E-02
ENSDARG00000014646	aoc2	151	1529	2,73	2,52E-02
ENSDARG00000088140	hsd17b7	2	28	2,72	2,51E-02
ENSDARG00000062797	nell2b	13	108	2,66	3,42E-03
ENSDARG00000007971	cks1b	3	37	2,65	4,23E-02
ENSDARG00000057610	cdkn2c	2	23	2,64	2,69E-02
ENSDARG00000091234	si:ch73-335l21.4	0	40	2,63	8,17E-11
ENSDARG00000057706	si:ch211-137i24.10	4	124	2,61	2,56E-02
ENSDARG00000061249	myom1a	19	164	2,59	3,13E-02
ENSDARG00000078828	npb	29	292	2,55	2,36E-02
ENSDARG00000062346	cacna1ea	18	145	2,54	6,91E-03
ENSDARG00000024967	fancg	4	36	2,51	1,77E-02
ENSDARG00000040907	gcgb	695	7351	2,51	2,81E-02
ENSDARG00000090401	CABZ01020840.1	11	86	2,50	4,40E-02
ENSDARG00000045834	si:dkey-14d8.7	221	1653	2,49	4,40E-02
ENSDARG00000069752	ckba	28	234	2,47	3,16E-02
ENSDARG00000025667	adgrb2	1	22	2,47	3,41E-02
ENSDARG00000014967	g6pcb	8208	61158	2,45	4,68E-02
ENSDARG00000069681	pcgf6	7	45	2,42	1,05E-02

ENSDARG00000039034	marcksl1a	2	20	2,39	4,12E-02
ENSDARG00000079296	gcga	2295	20588	2,39	3,38E-02
ENSDARG00000096908	si:ch73-185c24.2	5	48	2,37	4,69E-02
ENSDARG00000074597	lzts2a	4	25	2,37	4,43E-02
ENSDARG00000038222	tyw3	7	48	2,35	2,76E-02
ENSDARG00000093549	selenop	4	78	2,32	3,47E-02
ENSDARG00000075027	fgfr1bl	6	37	2,26	3,30E-02
ENSDARG00000117089	CKS2	3	20	2,25	4,32E-02
ENSDARG00000014499	nutf2l	14	73	2,23	3,68E-03
ENSDARG00000038812	e2f5	18	107	2,22	3,69E-02
ENSDARG00000037097	slc7a2	37	235	2,20	3,15E-02
ENSDARG00000094557	nupr1	22	182	2,14	4,40E-02
ENSDARG00000067848	nmrk2	20	119	2,13	4,72E-02
ENSDARG00000096603	bmb	47	228	2,12	2,36E-02
ENSDARG00000014804	cacna2d1a	2	19	2,11	4,32E-02
ENSDARG00000019302	mid1ip1b	312	1468	2,08	8,19E-03
ENSDARG00000032799	kcnd2	76	340	2,08	1,13E-02
ENSDARG00000075706	si:ch211-106h4.9	11	63	2,07	4,59E-02
ENSDARG00000031317	ppdpfb	28857	126990	2,04	9,38E-03
ENSDARG00000100513	CABZ01079011.1	555	2441	2,04	1,69E-03
ENSDARG00000054177	adgra1a	8	44	2,02	3,19E-02
ENSDARG00000033971	prrx1a	18	89	1,99	4,79E-02
ENSDARG00000032765	net1	9	52	1,97	4,98E-02
ENSDARG00000002988	tnnt2d	16	68	1,95	2,35E-02
ENSDARG00000076010	twist1b	19	83	1,94	2,69E-02
ENSDARG00000043131	BX664625.1	10	46	1,94	4,12E-02
ENSDARG00000058117	snap25b	181	763	1,94	1,63E-02
ENSDARG00000014053	olfm1b	760	3258	1,93	2,35E-02
ENSDARG00000041411	rad51	7	31	1,93	4,38E-02
ENSDARG00000003635	mogat3b	14	58	1,91	2,52E-02
ENSDARG00000079236	si:dkey-253d23.11	5	21	1,91	2,44E-02
ENSDARG00000093008	adgrf3b	6	28	1,90	4,69E-02
ENSDARG00000042876	abracl	5	26	1,88	4,64E-02
ENSDARG00000019417	gadd45ga	967	3960	1,86	4,12E-02
ENSDARG00000061994	acacb	46	179	1,83	1,24E-02
ENSDARG00000076419	si:dkeyp-117b11.2	7	29	1,83	2,65E-02
ENSDARG00000105112	ntn1a	4	18	1,79	4,32E-02
ENSDARG00000099186	slc1a5	22	87	1,79	2,57E-02
ENSDARG00000100686	si:ch211-241b2.4	7	29	1,78	4,23E-02
ENSDARG00000079659	wdr53	8	33	1,78	3,30E-02
ENSDARG00000062152	chaf1a	17	72	1,77	4,41E-02
ENSDARG00000103712	plpp7	10	43	1,74	4,23E-02
ENSDARG00000013950	pdf	10	39	1,74	2,84E-02
ENSDARG00000079783	isg20	59	212	1,72	2,01E-02
ENSDARG00000006782	rb1	6	23	1,72	4,32E-02
ENSDARG00000042977	nfil3	11	41	1,72	4,64E-02
ENSDARG00000036775	dtd2	32	118	1,71	2,72E-02
ENSDARG00000078077	dph2	12	45	1,69	4,06E-02
ENSDARG00000069708	cdc34a	10	37	1,69	3,84E-02
ENSDARG00000079815	lyrm7	42	144	1,66	2,69E-02
ENSDARG00000004274	zgc:112496	13	47	1,63	4,40E-02
ENSDARG00000012044	polr3gla	10	37	1,63	4,59E-02
ENSDARG00000092780	si:ch1073-170o4.1	14	48	1,63	4,79E-02
ENSDARG00000060126	acyp1	62	210	1,63	4,12E-02
ENSDARG00000062702	ankmy1	25	88	1,61	3,76E-02
ENSDARG00000042659	thyn1	33	109	1,61	4,32E-02
ENSDARG00000114589	jam3a	210	655	1,60	4,44E-03
ENSDARG00000104181	tmsb1	5059	16286	1,60	4,12E-02
ENSDARG00000060445	elp6	41	132	1,59	2,17E-02
ENSDARG00000101367	si:ch73-193i22.1	121	393	1,59	2,76E-02
ENSDARG00000078683	RNF14 (1 of many)	39	128	1,59	2,81E-02
ENSDARG00000015050	calm3a	3052	9638	1,58	2,21E-02
ENSDARG00000036878	sh3bgrl2	156	516	1,58	4,64E-02

ENSDARG00000088178	tp53inp2	13	42	1,57	4,99E-02
ENSDARG00000070012	sesn2	116	361	1,57	2,22E-02
ENSDARG00000024311	nsmce4a	9	30	1,57	4,64E-02
ENSDARG00000030805	znhit2	14	45	1,56	1,94E-02
ENSDARG00000054540	imp4	31	95	1,54	1,06E-02
ENSDARG00000035427	surf6	11	35	1,52	3,63E-02
ENSDARG00000051749	tigara	20	63	1,52	4,79E-02
ENSDARG00000054696	psmb4	182	549	1,52	1,83E-02
ENSDARG00000061314	ngrn	38	115	1,51	3,00E-02
ENSDARG00000037188	rpa2	39	117	1,50	2,68E-02
ENSDARG00000101110	LYRM4	34	100	1,50	1,31E-02
ENSDARG00000068629	cd151l	88	261	1,49	1,60E-02
ENSDARG00000076667	ccng1	2624	7540	1,49	1,25E-03
ENSDARG00000102822	pam16	47	141	1,49	3,30E-02
ENSDARG00000060338	trmt12	21	65	1,48	4,59E-02
ENSDARG00000045155	phf5a	36	107	1,48	2,72E-02
ENSDARG00000026577	cdk2	22	66	1,47	3,88E-02
ENSDARG00000043728	srek1ip1	27	78	1,45	1,98E-02
ENSDARG00000063474	arfgef1	274	771	1,44	1,97E-02
ENSDARG00000075616	polr2k	50	148	1,44	4,41E-02
ENSDARG00000053452	pop5	21	59	1,43	2,44E-02
ENSDARG00000003941	rrs1	57	160	1,42	2,76E-02
ENSDARG00000103484	si:ch211-215k15.5	39	110	1,41	3,16E-02
ENSDARG00000058394	tagln3b	280	789	1,41	3,79E-02
ENSDARG00000006434	polr1e	40	112	1,41	2,12E-02
ENSDARG00000099439	serp1	690	1905	1,41	1,51E-02
ENSDARG00000011360	nfatc2ip	26	73	1,41	4,41E-02
ENSDARG00000112476	CABZ01048143.1	55	153	1,41	2,22E-02
ENSDARG00000014017	rrm1	23	63	1,40	4,32E-02
ENSDARG00000076568	sec61b	1064	2942	1,40	2,78E-02
ENSDARG00000074305	si:ch73-257c13.2	28	78	1,39	4,24E-02
ENSDARG00000017728	phb2b	119	326	1,39	2,46E-02
ENSDARG00000018953	gclm	41	111	1,39	1,47E-02
ENSDARG00000071594	TIMM21	42	116	1,38	3,58E-02
ENSDARG00000071403	oaz1a	1089	2995	1,38	3,88E-02
ENSDARG00000097630	si:ch211-282b22.1	45	122	1,38	2,68E-02
ENSDARG00000002613	rpa3	26	71	1,37	3,69E-02
ENSDARG00000039347	rps24	1025	2721	1,36	1,51E-02
ENSDARG00000060041	lig1	35	94	1,35	4,38E-02
ENSDARG00000036924	tatdn1	30	80	1,35	4,98E-02
ENSDARG00000094300	si:ch211-160e1.5	243	641	1,33	3,30E-02
ENSDARG00000037108	pfdn6	135	351	1,33	2,18E-02
ENSDARG00000020623	baxa	50	131	1,33	1,60E-02
ENSDARG00000090872	si:dkey-276j7.1	181	473	1,32	2,36E-02
ENSDARG00000020895	bag1	141	367	1,32	2,44E-02
ENSDARG00000069082	mrm3b	21	55	1,32	4,12E-02
ENSDARG00000005772	tsr2	23	62	1,32	4,32E-02
ENSDARG00000013938	psmb3	544	1425	1,32	3,70E-02
ENSDARG00000023852	lsm4	114	293	1,30	2,61E-02
ENSDARG00000111785	trappc2l	162	425	1,30	4,79E-02
ENSDARG00000057805	mrpl28	96	243	1,30	1,55E-02
ENSDARG00000038288	cd151	70	180	1,30	3,15E-02
ENSDARG00000086699	smim15	40	103	1,30	2,65E-02
ENSDARG00000039719	mrps26	29	76	1,30	2,81E-02
ENSDARG00000101979	eps8l3a	47	121	1,29	4,20E-02
ENSDARG00000037835	polr3k	86	222	1,29	3,69E-02
ENSDARG00000113969	supt4h1	137	356	1,29	4,79E-02
ENSDARG00000090386	cd3eap	66	170	1,28	4,38E-02
ENSDARG00000099997	cox20	70	177	1,28	2,34E-02
ENSDARG00000104709	sprtn	24	62	1,28	4,32E-02
ENSDARG00000042660	fam32a	241	605	1,28	2,81E-02
ENSDARG00000076518	mettl1	60	154	1,28	4,64E-02
ENSDARG00000063665	mat2al	389	956	1,27	2,22E-02

ENSDARG00000017093	emc8	307	757	1,27	1,15E-02
ENSDARG00000042708	tuba8l	224	567	1,26	4,54E-02
ENSDARG00000039400	polr1c	110	273	1,26	4,23E-02
ENSDARG00000071733	si:ch211-207i20.3	36	89	1,25	4,41E-02
ENSDARG00000003846	mrpl21	35	89	1,25	4,69E-02
ENSDARG00000045159	zdhhc4	43	108	1,25	4,88E-02
ENSDARG00000104227	nop10	67	165	1,24	4,47E-02
ENSDARG00000071500	fam207a	36	89	1,24	4,98E-02
ENSDARG00000094833	coa5	91	226	1,24	4,69E-02
ENSDARG00000071306	gip	1	483	1,23	1,51E-02
ENSDARG00000092677	coa6	70	171	1,23	3,15E-02
ENSDARG00000013966	psma6b	112	277	1,23	4,98E-02
ENSDARG00000021753	ccdc25	222	533	1,23	1,12E-02
ENSDARG00000013478	rrp15	43	105	1,22	3,58E-02
ENSDARG00000009640	psmb1	707	1714	1,22	3,47E-02
ENSDARG00000028198	ube2v2	723	1752	1,22	3,96E-02
ENSDARG00000102846	trmt13	16	40	1,21	4,64E-02
ENSDARG00000104058	si:dkey-31c13.1	26	62	1,20	3,15E-02
ENSDARG00000099667	snrpg	106	256	1,20	4,32E-02
ENSDARG00000012222	nup35	54	127	1,19	1,30E-02
ENSDARG00000045797	si:ch211-68a17.7	115	273	1,19	4,62E-02
ENSDARG00000006567	dus4l	28	66	1,19	3,61E-02
ENSDARG00000000760	znf511	44	104	1,19	3,58E-02
ENSDARG00000067777	zgc:158640	37	89	1,19	4,40E-02
ENSDARG00000011272	uqcc2	131	311	1,18	4,32E-02
ENSDARG00000025326	myl12.2	762	1784	1,18	4,32E-02
ENSDARG00000070985	mrps21	82	194	1,18	4,24E-02
ENSDARG00000059725	hypk	185	433	1,18	3,69E-02
ENSDARG00000042825	rnf169	78	182	1,17	2,48E-02
ENSDARG00000068305	mrps14	238	555	1,17	3,58E-02
ENSDARG00000030537	psmc1a	319	740	1,17	2,83E-02
ENSDARG00000033171	trappc6bl	62	142	1,17	2,91E-02
ENSDARG00000100585	coa3a	143	336	1,17	4,97E-02
ENSDARG00000042345	si:dkeyp-55f12.3	88	208	1,17	4,98E-02
ENSDARG00000024920	tmem136a	42	99	1,17	2,81E-02
ENSDARG00000103101	NDUFC1	251	581	1,17	3,30E-02
ENSDARG00000071013	arl6ip6	120	275	1,17	2,36E-02
ENSDARG00000020841	rbm7	21	48	1,16	4,88E-02
ENSDARG00000070824	ndufb5	224	519	1,16	4,50E-02
ENSDARG00000075129	mrps11	65	150	1,15	4,01E-02
ENSDARG00000031981	pcbd1	439	996	1,15	2,76E-02
ENSDARG00000006074	uck2a	120	274	1,15	2,81E-02
ENSDARG00000027899	sem1	485	1105	1,15	2,81E-02
ENSDARG00000008020	med31	100	226	1,15	1,12E-02
ENSDARG00000052020	cmtm4	141	321	1,15	3,88E-02
ENSDARG00000070037	rgs8	365	826	1,15	4,64E-02
ENSDARG00000002967	pdap1b	137	312	1,15	3,38E-02
ENSDARG00000003757	cnpy1	884	2016	1,14	4,32E-02
ENSDARG00000039958	utp11l	64	147	1,14	3,93E-02
ENSDARG00000071468	mrpl54	76	172	1,14	3,45E-02
ENSDARG00000014165	ssr3	811	1838	1,13	4,23E-02
ENSDARG00000111714	zgc:112271	91	206	1,13	4,59E-02
ENSDARG00000045843	apex1	47	106	1,13	2,81E-02
ENSDARG00000069671	maip1	71	159	1,13	3,69E-02
ENSDARG00000012603	eif3ja	92	206	1,11	4,64E-02
ENSDARG00000037932	lin7b	117	259	1,11	4,24E-02
ENSDARG00000024465	mrps18a	144	319	1,11	3,64E-02
ENSDARG00000035678	zgc:91910	341	751	1,11	2,81E-02
ENSDARG00000014536	pfdn1	190	422	1,11	4,32E-02
ENSDARG00000063924	mt-cyb	14766	32251	1,10	2,44E-02
ENSDARG00000043857	ufm1	629	1398	1,10	4,69E-02
ENSDARG00000099309	si:zfos-169g10.3	153	336	1,10	4,11E-02
ENSDARG00000091001	mycbp	179	392	1,10	3,30E-02

ENSDARG00000043865	timm9	44	99	1,10	4,98E-02
ENSDARG00000104901	ostc	572	1257	1,10	3,41E-02
ENSDARG00000011841	atp5l	730	1619	1,10	4,97E-02
ENSDARG00000026482	arhgap12b	320	697	1,09	4,29E-02
ENSDARG00000012450	vmp1	280	605	1,09	2,05E-03
ENSDARG00000068436	h3f3b.1	305	671	1,09	4,64E-02
ENSDARG00000037962	psmb7	603	1318	1,09	4,12E-02
ENSDARG00000028379	trub2	62	136	1,09	4,99E-02
ENSDARG00000004527	pin4	106	230	1,09	3,69E-02
ENSDARG00000070916	dnajc27	27	59	1,08	4,99E-02
ENSDARG00000018562	cope	1308	2846	1,08	3,83E-02
ENSDARG00000037260	toporsa	40	89	1,08	4,71E-02
ENSDARG00000069241	ttc32	56	122	1,07	4,96E-02
ENSDARG00000005251	rpe	219	469	1,07	4,23E-02
ENSDARG00000036704	tmem18	154	328	1,07	1,12E-02
ENSDARG00000094191	nup37	48	103	1,07	4,59E-02
ENSDARG00000035146	polr2j	57	124	1,07	4,22E-02
ENSDARG00000071426	lrrc59	122	265	1,07	4,97E-02
ENSDARG00000067593	sco1	94	200	1,06	4,29E-02
ENSDARG00000063341	si:ch211-284b7.3	209	447	1,05	4,69E-02
ENSDARG00000097696	si:ch73-71c20.5	151	323	1,05	4,68E-02
ENSDARG00000053070	gosr2	300	636	1,05	3,69E-02
ENSDARG00000095086	cfdp1	46	100	1,05	4,64E-02
ENSDARG00000095273	cox8a	437	932	1,05	4,32E-02
ENSDARG00000032369	btbd6b	648	1364	1,05	4,74E-02
ENSDARG00000098355	atp5pd	976	2069	1,05	4,40E-02
ENSDARG00000002690	rabif	107	226	1,05	4,40E-02
ENSDARG00000098942	rp9	85	180	1,04	2,85E-02
ENSDARG00000039872	lamtor2	246	519	1,04	4,12E-02
ENSDARG00000035985	fam210aa	235	490	1,04	8,02E-03
ENSDARG00000040966	zgc:113149	47	99	1,04	4,99E-02
ENSDARG00000035663	zgc:103438	125	261	1,03	4,32E-02
ENSDARG00000022795	golt1ba	77	162	1,03	4,69E-02
ENSDARG00000035860	rps28	747	1563	1,03	4,64E-02
ENSDARG00000092873	zgc:193541	545	1137	1,03	4,68E-02
ENSDARG00000116835	park7	544	1131	1,03	4,41E-02
ENSDARG00000011555	spag7	83	171	1,02	2,36E-02
ENSDARG00000003573	dctn5	197	407	1,02	2,81E-02
ENSDARG00000038030	rbx1	377	781	1,02	3,30E-02
ENSDARG00000087456	NDUFB1	345	709	1,01	2,72E-02
ENSDARG00000005738	rpf1	118	245	1,01	4,38E-02
ENSDARG00000115557	cox7b	1002	2068	1,01	4,62E-02
ENSDARG00000029252	ssb	58	119	1,01	4,32E-02
ENSDARG00000015236	pycr3	32	67	1,01	4,25E-02
ENSDARG00000018334	rpl35	1003	2068	1,01	4,69E-02
ENSDARG00000113503	CU459094.2	58	119	1,01	2,57E-02
ENSDARG00000105036	arl6ip4	77	157	1,00	4,24E-02
ENSDARG00000013075	mrpl11	133	273	1,00	4,88E-02
ENSDARG00000078822	ccdc18	124	60	-1,01	4,51E-02
ENSDARG00000087573	hdac11	252	124	-1,01	5,64E-03
ENSDARG00000020759	elf1	1747	842	-1,01	4,64E-02
ENSDARG00000098304	taok1b	290	142	-1,01	3,30E-02
ENSDARG00000061504	kdm4c	836	404	-1,01	4,40E-02
ENSDARG00000010281	xpo4	145	69	-1,02	4,41E-02
ENSDARG00000107385	aars2	179	85	-1,02	4,71E-02
ENSDARG00000030824	esyt2a	304	147	-1,03	4,23E-02
ENSDARG00000098783	polr2b	329	157	-1,03	3,62E-02
ENSDARG00000023694	spon1b	6256	3012	-1,03	1,27E-02
ENSDARG00000023920	llgl2	374	181	-1,03	1,47E-02
ENSDARG00000037593	prickle2b	1070	508	-1,03	4,32E-02
ENSDARG00000006094	igf2r	595	284	-1,04	4,40E-02
ENSDARG00000062055	rnf38	366	175	-1,04	4,41E-02
ENSDARG00000102252	USP34	850	402	-1,04	4,71E-02

ENSDARG00000060835	tecpr2	272	128	-1,04	3,69E-02
ENSDARG00000062020	gse1	282	136	-1,05	2,81E-02
ENSDARG00000000966	ncor2	4415	2082	-1,05	4,32E-02
ENSDARG00000070371	kat7a	435	205	-1,05	4,89E-02
ENSDARG00000026548	cyp2u1	210	98	-1,05	4,69E-02
ENSDARG00000063352	inpp4aa	280	132	-1,05	4,29E-02
ENSDARG00000104435	si:ch73-217b7.1	372	175	-1,06	2,91E-02
ENSDARG00000075670	rereb	549	256	-1,07	4,59E-02
ENSDARG00000113393	CABZ01080601.1	192	89	-1,07	4,38E-02
ENSDARG00000074702	arfgef2	483	224	-1,07	3,54E-02
ENSDARG00000053200	dis3l	88	40	-1,08	4,06E-02
ENSDARG00000013224	phc2b	85	39	-1,08	4,56E-02
ENSDARG00000069970	lingo2b	478	219	-1,08	3,41E-02
ENSDARG00000016830	rimkla	389	178	-1,09	3,88E-02
ENSDARG00000061378	smg8	166	76	-1,09	2,46E-02
ENSDARG00000041702	rfx6	2696	1222	-1,09	4,03E-02
ENSDARG00000069208	stx7l	131	59	-1,10	4,32E-02
ENSDARG00000036442	atp11c	332	151	-1,11	2,21E-02
ENSDARG00000092359	si:dkeyp-113d7.10	94	43	-1,12	2,21E-02
ENSDARG00000018296	rev1	196	88	-1,12	4,41E-02
ENSDARG00000099449	atrxl	344	154	-1,12	2,81E-02
ENSDARG00000037066	lgals8a	77	34	-1,13	4,06E-02
ENSDARG00000079104	mfhas1	365	160	-1,14	4,42E-02
ENSDARG00000062553	bach1a	225	99	-1,15	4,62E-02
ENSDARG00000098592	zgc:92140	344	151	-1,15	3,49E-02
ENSDARG00000061282	glg1a	1704	736	-1,16	4,32E-02
ENSDARG00000004336	exoc4	784	338	-1,16	4,29E-02
ENSDARG00000076895	flrt3	1589	679	-1,17	4,69E-02
ENSDARG00000103361	tshz3b	851	364	-1,18	4,23E-02
ENSDARG00000074169	gpam	260	111	-1,18	3,83E-02
ENSDARG00000063007	apc2	666	284	-1,19	3,58E-02
ENSDARG00000062258	armc8	151	64	-1,19	2,52E-02
ENSDARG00000056165	si:ch211-215a10.4	174	72	-1,20	4,62E-02
ENSDARG00000076943	lhfpl4b	250	104	-1,20	4,50E-02
ENSDARG00000074558	cntnap2b	536	223	-1,21	4,99E-02
ENSDARG00000061021	fam160a1a	136	56	-1,21	4,32E-02
ENSDARG00000098909	SHC3	194	80	-1,22	4,64E-02
ENSDARG00000103390	cacna2d2a	1930	779	-1,22	4,89E-02
ENSDARG00000035655	rad21b	162	67	-1,22	4,62E-02
ENSDARG00000089357	miga2	155	63	-1,23	4,69E-02
ENSDARG00000010023	CU856539.1	616	252	-1,23	4,24E-02
ENSDARG00000087921	plce1	1181	478	-1,24	4,62E-02
ENSDARG00000104101	si:ch73-287m6.1	643	261	-1,24	3,38E-02
ENSDARG00000073905	zgc:92481	175	70	-1,25	4,69E-02
ENSDARG00000060257	zranb1a	504	206	-1,27	3,44E-03
ENSDARG00000063253	hecw2b	77	31	-1,27	2,31E-02
ENSDARG00000069006	tle3b	690	271	-1,28	3,54E-02
ENSDARG00000077656	arfgef3	3627	1404	-1,29	4,59E-02
ENSDARG00000026784	robo1	1453	577	-1,29	2,22E-02
ENSDARG00000061656	ptpdc1a	91	36	-1,29	1,83E-02
ENSDARG00000021405	chd3	425	168	-1,29	3,06E-02
ENSDARG00000090844	CABZ01054965.1	211	81	-1,30	4,98E-02
ENSDARG00000103288	PDPR	201	79	-1,30	2,01E-02
ENSDARG00000034893	rarab	172	67	-1,31	1,47E-02
ENSDARG00000078924	tmco4	68	25	-1,31	4,44E-02
ENSDARG00000059870	clk2b	150	57	-1,31	3,26E-02
ENSDARG00000035452	camkk1a	542	212	-1,31	1,69E-02
ENSDARG00000105056	znf45l	43	16	-1,32	4,64E-02
ENSDARG00000000324	dcaf8	194	73	-1,32	4,20E-02
ENSDARG00000088689	si:ch211-201o1.1	276	108	-1,32	1,91E-02
ENSDARG00000103746	mapkbp1	129	48	-1,34	4,79E-02
ENSDARG00000101270	klhl8	190	71	-1,34	2,81E-02
ENSDARG00000059557	xylt2	123	46	-1,35	4,41E-02

ENSDARG00000008209	myt1la	89	34	-1,35	4,98E-02
ENSDARG00000052658	pptc7b	176	66	-1,36	3,45E-02
ENSDARG00000043332	prkcz	340	128	-1,36	2,48E-02
ENSDARG00000093093	adam17b	458	169	-1,36	3,44E-02
ENSDARG00000054186	ncs1b	158	57	-1,38	3,15E-02
ENSDARG00000098405	map3k1	488	176	-1,38	4,41E-02
ENSDARG00000062812	glsa	1103	396	-1,39	3,69E-02
ENSDARG00000036096	smad3a	255	93	-1,39	3,58E-02
ENSDARG00000018179	acvrl1	87	32	-1,40	2,36E-02
ENSDARG00000089292	adgrl1a	206	72	-1,40	4,72E-02
ENSDARG00000062325	shank2	266	96	-1,41	2,36E-02
ENSDARG00000103792	CABZ01076737.1	143	51	-1,41	3,06E-02
ENSDARG00000035660	si:ch211-193k19.1	703	251	-1,42	2,57E-02
ENSDARG00000052094	notch1b	727	250	-1,43	4,32E-02
ENSDARG00000086990	carmil3	731	259	-1,43	2,01E-02
ENSDARG00000060018	si:ch1073-44g3.1	61	21	-1,44	4,71E-02
ENSDARG00000006272	mpp5a	181	62	-1,44	4,23E-02
ENSDARG00000043858	cdk19	58	20	-1,45	3,26E-02
ENSDARG00000062177	dcbld2	743	259	-1,46	1,91E-02
ENSDARG00000104295	arhgap21a	783	277	-1,46	3,68E-03
ENSDARG00000079616	cramp1	309	106	-1,49	1,73E-02
ENSDARG00000100456	ptk2aa	162	55	-1,49	2,67E-02
ENSDARG00000069014	nphp4	311	104	-1,49	2,52E-02
ENSDARG00000100854	ago4	215	72	-1,49	2,44E-02
ENSDARG00000113064	CR392045.1	186	60	-1,51	4,99E-02
ENSDARG00000100708	jakmip3	85	28	-1,52	2,34E-02
ENSDARG00000052978	mbnl1	653	212	-1,52	3,58E-02
ENSDARG00000037894	ezh1	105	33	-1,53	4,23E-02
ENSDARG00000077839	dhx30	107	34	-1,53	4,23E-02
ENSDARG00000079396	lrfn5b	113	36	-1,53	3,19E-02
ENSDARG00000018726	slco3a1	80	26	-1,54	4,24E-02
ENSDARG00000077364	mapk9	100	31	-1,54	2,81E-02
ENSDARG00000067801	SEMA4D	100	32	-1,55	4,59E-02
ENSDARG00000011222	ccdc28b	88	28	-1,55	2,62E-02
ENSDARG00000004634	osbp	512	157	-1,56	4,40E-02
ENSDARG00000099945	CU467646.4	36	11	-1,57	3,88E-02
ENSDARG00000002607	unm_sa1614	1123	348	-1,58	2,69E-02
ENSDARG00000113111	DGKD	178	55	-1,59	4,32E-02
ENSDARG00000102902	strn4	88	27	-1,59	3,93E-02
ENSDARG00000042128	pacsin1b	76	23	-1,60	3,88E-02
ENSDARG00000063310	oxr1b	638	189	-1,61	3,83E-02
ENSDARG00000036826	ankrd52a	157	48	-1,62	2,81E-02
ENSDARG00000001014	myh9b	394	116	-1,62	4,68E-02
ENSDARG00000059411	impa2	301	93	-1,63	7,94E-03
ENSDARG00000011333	arhgap33	135	40	-1,64	3,88E-02
ENSDARG00000027159	fbxo28	398	114	-1,64	4,32E-02
ENSDARG00000071032	pnkd	42	12	-1,66	3,37E-02
ENSDARG00000077162	nwd2	69	21	-1,67	2,46E-02
ENSDARG00000018553	ifih1	90	26	-1,67	3,45E-02
ENSDARG00000063568	syt7a	571	153	-1,72	4,51E-02
ENSDARG00000077022	fam131a	101	28	-1,73	1,95E-02
ENSDARG00000075173	si:ch1073-322p19.1	80	21	-1,74	4,98E-02
ENSDARG00000058771	nav1b	179	47	-1,76	4,72E-02
ENSDARG00000030289	jag1a	128	33	-1,79	4,12E-02
ENSDARG00000062220	plekha7b	131	36	-1,81	3,62E-03
ENSDARG00000098031	si:ch73-144d13.8	23	6	-1,81	4,32E-02
ENSDARG00000102234	si:ch211-71n6.4	216	55	-1,82	2,83E-02
ENSDARG00000044436	rps6ka4	83	22	-1,85	1,12E-02
ENSDARG00000092067	atn1	66	16	-1,86	4,23E-02
ENSDARG00000100587	si:ch211-235i11.7	41	9	-1,89	2,01E-02
ENSDARG00000003655	tmem59l	331	79	-1,89	4,12E-02
ENSDARG00000053535	lmo7b	41	9	-1,90	4,50E-02
ENSDARG00000056181	ncam1a	494	119	-1,92	2,46E-02

ENSDARG00000079547	zbtb47b	78	17	-1,94	4,64E-02
ENSDARG00000045023	lhfpl5a	57	13	-1,97	1,51E-02
ENSDARG00000020845	tns1b	1300	303	-1,99	4,07E-03
ENSDARG00000063580	ppp1r9ala	70	16	-2,00	1,47E-02
ENSDARG00000055752	npas4a	3702	814	-2,01	2,52E-02
ENSDARG00000074034	si:dkey-91i10.2	30	6	-2,02	2,81E-02
ENSDARG00000079414	sez6b	848	162	-2,02	4,32E-02
ENSDARG00000018382	prkcha	44	8	-2,04	4,74E-02
ENSDARG00000103903	LO017650.1	60	11	-2,04	4,99E-02
ENSDARG00000016749	slco1c1	40	8	-2,05	4,94E-02
ENSDARG00000077799	egr4	12118	2579	-2,07	1,27E-02
ENSDARG00000079251	nlgn2b	207	44	-2,09	1,12E-02
ENSDARG00000089156	egr3	47	10	-2,11	4,40E-02
ENSDARG00000056797	ppp2r2ca	101	20	-2,13	1,60E-02
ENSDARG00000102277	lama1	30	5	-2,16	4,69E-02
ENSDARG00000055459	dlgap3	91	15	-2,17	4,32E-02
ENSDARG00000054323	pparab	68	14	-2,20	5,64E-03
ENSDARG00000014652	ankrd13b	70	13	-2,21	2,22E-02
ENSDARG00000087529	zgc:85936	37	7	-2,22	2,35E-02
ENSDARG00000089860	atxn7l1	149	28	-2,22	8,98E-03
ENSDARG00000035875	tmem54b	523	87	-2,23	2,65E-02
ENSDARG00000059534	si:ch211-146m13.3	72	13	-2,26	4,44E-03
ENSDARG00000102111	zbtb49	27	4	-2,27	3,47E-02
ENSDARG00000079581	nyap2a	105	16	-2,32	2,69E-02
ENSDARG00000051915	pde5ab	82	13	-2,35	2,62E-02
ENSDARG00000079519	CR456624.1	69	11	-2,38	2,21E-02
ENSDARG00000058558	lzts3b	41	5	-2,50	4,30E-02
ENSDARG00000054036	rnf34b	82	10	-2,57	2,44E-02
ENSDARG00000032795	tmem8a	175	20	-2,66	2,91E-02
ENSDARG00000106564	CT027676.1	216	25	-2,66	2,46E-02
ENSDARG00000109783	igsf11	26	3	-2,74	1,61E-02
ENSDARG00000105210	etf1a	39	3	-2,82	4,32E-02
ENSDARG00000076127	gabrb1	88	5	-2,86	4,75E-02
ENSDARG00000044098	egr2a	89	8	-2,94	2,18E-02
ENSDARG00000079252	fam117ab	20	1	-3,02	2,49E-02
ENSDARG00000004586	ntrk1	60	3	-3,25	3,88E-02
ENSDARG00000013838	sulf2b	136	8	-3,47	3,68E-03
ENSDARG00000095891	si:ch73-30l9.1	27	1	-3,49	1,47E-02
ENSDARG00000070919	cpne5b	55	1	-3,84	1,69E-02
