# Supplementary material for: A δ-cell subpopulation with a pro-β-cell identity contributes to efficient age-independent recovery in a zebrafish model of diabetes
Source: eLife. 2022 Jan 21;11:e67576. doi: 10.7554/eLife.67576 (PMC8820734; doi:10.7554/eLife.67576)
Supplement: Figure 6—source data 3. [file elife-67576-fig6-data3.pdf]

Figure 6-Source Data 3. GO analysis of genes overexpressed in bihormonal cells

Overrepresented GO in 20 dpt bihormonal cells (versus sst1.1:GFPhigh δ-cells) (DESeq FC>2x, Padj<0.05)
GO identified by WebGestalt (FDR<0.25)

Biological Process (non redundant)

geneSet	description	enrichmentRatio	pValue	FDR	userId
GO:0006396	RNA processing	3,635759945	2,23E-07	6,32E-05	imp4;srek1ip1;elp6;pop5;lsm4;phf5a;rrs1;tyw3;rp9;rrp15;dus4l;polr3k;arl6ip4;ssb;snrpg;nop10;trmt12;rps28;mettl1;rpl35;fam207a
GO:0034660	ncRNA metabolic process	4,725045167	5,51E-07	7,82E-05	imp4;elp6;pop5;dtd2;rrs1;tyw3;rrp15;dus4l;polr3k;nop10;trmt12;rps28;mettl1;rpl35;fam207a
GO:0022613	ribonucleoprotein complex biogenesis	3,6688586	1,95E-04	0,0185002	imp4;pop5;lsm4;rrs1;rrp15;snrpg;nop10;eif3ja;rps28;rpl35;fam207a
GO:0009451	RNA modification	4,58701014	0,001917786	0,136162841	elp6;tyw3;dus4l;nop10;trmt12;mettl1
GO:0006360	transcription by RNA polymerase I	10,20609756	0,002942021	0,16710682	polr1e;cd3eap;polr2k

KEGG pathways

geneSet	description	enrichmentRatio	pValue	FDR	userId
dre03050	Proteasome	8,323033708	1,73E-05	0,001501437	psmb4;sem1;psmc1a;psmb1;psmb3;psmb7;psma6b
dre04115	p53 signaling pathway	6,742710852	2,06E-05	0,001501437	ccnd1;ccng1;igf1;baxa;sesn2;cdk2;gadd45ga;ccnb1
dre03010	Ribosome	4,968975348	2,78E-05	0,001501437	rps24;mrpl28;mrps14;mrps18a;mrps11;mrps21;rps28;mrpl21;rpl35;mrpl11
dre03020	RNA polymerase	11,48004649	6,10E-05	0,002469881	polr1e;polr3k;polr2j;polr1c;polr2k
dre04110	Cell cycle	4,311211705	2,14E-04	0,006948751	ccnd1;cdkn2c;rbx1;e2f5;cdk2;gadd45ga;ccnb1;rb1;mad2l1
dre00240	Pyrimidine metabolism	4,438951311	9,39E-04	0,025347892	polr1e;uck2a;polr3k;polr2j;polr1c;rrm1;polr2k
dre03440	Homologous recombination	6,658426966	0,002875844	0,066555238	rpa2;sem1;rpa3;rad51
dre03420	Nucleotide excision repair	6,053115424	0,004081581	0,073836301	rpa2;rbx1;rpa3;lig1
dre03430	Mismatch repair	9,079673136	0,004102017	0,073836301	rpa2;rpa3;lig1
dre04114	Oocyte meiosis	3,353164659	0,004666011	0,07558938	ins;igf1;calm3a;rbx1;cdk2;ccnb1;mad2l1
dre03460	Fanconi anemia pathway	5,22229566	0,006939264	0,102196431	fancg;rpa2;rpa3;rad51
dre04068	FoxO signaling pathway	2,790957411	0,012353906	0,166777726	ins;ccnd1;igf1;cdk2;gadd45ga;ccnb1;g6pcb
dre03030	DNA replication	5,256652868	0,018914894	0,235708676	rpa2;rpa3;lig1
dre04218	Cellular senescence	2,505859611	0,02118965	0,245033418	ccnd1;calm3a;e2f5;cdk2;gadd45ga;ccnb1;rb1
dre04914	Progesterone-mediated oocyte maturation	3,082605077	0,022688279	0,245033418	ins;igf1;cdk2;ccnb1;mad2l1
dre04623	Cytosolic DNA-sensing pathway	4,756019262	0,024662355	0,249706349	polr3k;polr1c;polr2k
