# Supplementary material for: A δ-cell subpopulation with a pro-β-cell identity contributes to efficient age-independent recovery in a zebrafish model of diabetes
Source: eLife. 2022 Jan 21;11:e67576. doi: 10.7554/eLife.67576 (PMC8820734; doi:10.7554/eLife.67576)
Supplement: Figure 6—source data 4. [file elife-67576-fig6-data4.pdf]

Figure 6-Source Data 4. GO analysis of genes overexpressed in sst1.1:GFPhigh δ-cells

Overrepresented GO in sst1.1:GFPhigh delta-cells (versus 20dpt bihormonal cells) (DESeq FC>2x, Padj<0.05)
GO identified by WebGestalt (FDR<0.25)

Biological Process (non redundant)

no significant BP in the gene set

KEGG pathways

geneSet	description	enrichmentRatio	pValue	FDR	userId
dre04330	Notch signaling pathway	8,635336976	0,00110464	0,08990274	adam17b;jag1a;ncor2;notch1b
dre04530	Tight junction	4,248028674	0,00110991	0,08990274	llgl2;ppp2r2ca;prkcz;mapk9;mpp5a;map3k1;myh9b
