# Supplementary material for: A δ-cell subpopulation with a pro-β-cell identity contributes to efficient age-independent recovery in a zebrafish model of diabetes
Source: eLife. 2022 Jan 21;11:e67576. doi: 10.7554/eLife.67576 (PMC8820734; doi:10.7554/eLife.67576)
Supplement: Figure 7—source data 1. [file elife-67576-fig7-data1.pdf]

Figure 7-Source Data 1

SST+ mCherry+ bihormonal cells in the ductal domain (tail, larvae)

CTL	NFP
1	0
0	0
1	1
0	5
0	15
1	2
0	8
1	3
0	0
2	4
0	6
0	23
0	7
0	6
0	9
0	6
0	5
0	19
0	5
0	7
	5
	2
	6
	4
	7
	4
